# The Chemical Landscape of Leaf Surfaces and Its Interaction with the Atmosphere

**DOI:** 10.1021/acs.chemrev.3c00763

**Published:** 2024-04-23

**Authors:** Rachele Ossola, Delphine Farmer

**Affiliations:** Department of Chemistry, Colorado State University, 80523 Fort Collins, Colorado (United States)

## Abstract

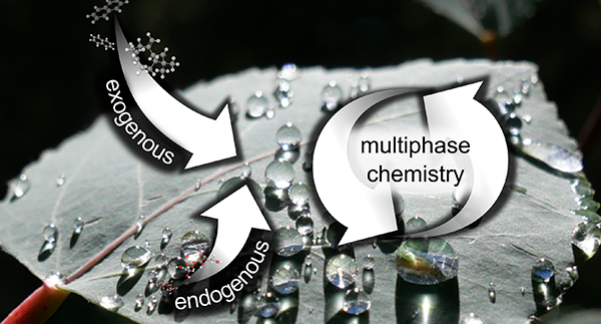

Atmospheric chemists have historically treated leaves as inert surfaces that merely emit volatile hydrocarbons. However, a growing body of evidence suggests that leaves are ubiquitous substrates for multiphase reactions–implying the presence of chemicals on their surfaces. This Review provides an overview of the chemistry and reactivity of the leaf surface’s “chemical landscape”, the dynamic ensemble of compounds covering plant leaves. We classified chemicals as endogenous (originating from the plant and its biome) or exogenous (delivered from the environment), highlighting the biological, geographical, and meteorological factors driving their contributions. Based on available data, we predicted ≫2 μg cm^–2^ of organics on a typical leaf, leading to a global estimate of ≫3 Tg for multiphase reactions. Our work also highlighted three major knowledge gaps: (i) the overlooked role of ambient water in enabling the leaching of endogenous substances and mediating aqueous chemistry; (ii) the importance of phyllosphere biofilms in shaping leaf surface chemistry and reactivity; (iii) the paucity of studies on the multiphase reactivity of atmospheric oxidants with leaf-adsorbed chemicals. Although biased toward available data, we hope this Review will spark a renewed interest in the leaf surface’s chemical landscape and encourage multidisciplinary collaborations to move the field forward.

## 1 Introduction

Vegetation covers much of the Earth’s surface, with plant leaves occupying an area comparable to the total land surface of our planet.^1^ While the atmospheric chemistry community has long recognized the role of plants as sources of biogenic volatile organic compounds, the potential for their surfaces, including leaves, to act as multiphase reaction sites has been underappreciated. Over the past few years, observational studies indicated that chemical reactions on leaves influence atmospheric concentrations of reactive trace gases, but efforts to confirm the occurrence of these processes and understand their mechanisms have been limited. This Review fills this gap by providing an overview of the variety of chemicals present on plant surfaces and their possible role in atmospheric chemistry processes.

Five sets of observations indicate that leaf surfaces may act as sites for reactions of atmospheric relevance. First, a few investigations described a large and ubiquitous source of formic acid in forest canopies^2−5^ and grasslands,^6^ but the origin of this compound has yet to be identified. Recent work showed that aqueous chemistry can mediate formic acid formation in clouds,^7^ and some authors suggested that an analogous process involving wet surfaces may occur in terrestrial ecosystems.^5,8,9^ Formic acid sources are still poorly represented in models, with implications for predicting rainwater acidity, gas-particle partitioning, and aerosol formation.^10,11^ Second, other research groups have observed substantial discrepancies between measured and modeled ozone fluxes on plants at high relative humidity, and posited the presence of a dry deposition mechanism involving ozonation of organic compounds present on leaf surfaces (reviewed by Clifton et al.^12^). Follow-up lab studies generally supported this hypothesis but could identify neither the nature of the compounds involved in this process, nor the factors controlling the large variability among plant species.^13,14^ Third, wet leaf cuticles mediate the bidirectional exchange of ammonia (reviewed by Flechard et al.^15^ and others^16,17^). This process takes place in the presence of leaf wetness and is primarily controlled by its acidity.^15,17^ In current models, only inorganic gas-phase acids (i.e., SO_2_) are accounted for when simulating the pH of leaf wetness; aerosols, soil particles, and other exogenous compounds have been pointed out as additional factors controlling surface wetness acidity but thorough evaluations are lacking.^16,17^ Leaf wetness has also been invoked in the bidirectional exchange of HONO,^18^ isocyanic acid,^4^ and alkanoic acids,^3,4^ and in the uptake of terpene oxidation products, organic peroxides, and other water-soluble oxygenates.^5^ Fourth, a recent modeling study hypothesized leaf surface chemistry to be an overlooked source of isoprene-derived secondary organic aerosols in the Amazon rainforest.^19^ The proposed mechanism involves the partitioning of gas-phase isoprene epoxydiols, main isoprene oxidation products,^20^ into leaf wetness followed by their acid-catalyzed hydrolysis to 2-methyltetrols. These semivolatile products may be released back in the gas phase and condense onto existing particles to yield isoprene-derived aerosols. Fifth, there has been contradictory evidence on the ability of leaf cuticles to act as sinks of peroxyacetyl nitrate, with field investigations reporting a larger contribution of nonstomatal over stomatal sinks as compared to lab studies.^13,16,21,22^ The different leaf surface chemistry of the species investigated is among the proposed causes of this discrepancy.

These lines of evidence challenge the idea of leaves as clean, inert, glass-like surfaces, implying the presence of compounds that can participate in multiphase reactions. (In this work, we follow the definition of Abbatt and Ravishankara^23^ and use “multiphase” to indicate reactions occurring between two phases, either gas–solid, gas–liquid, or liquid–solid.) In principle, these compounds can be produced *in situ* (by the plant and/or its associated biome) and excreted onto the surface of leaves or can be deposited from the atmosphere via wet or dry deposition. The leaf’s surface morphology, the chemical composition of the cuticle, and the presence and type of liquid water (i.e., surface wetness) define which compounds get adsorbed on the leaf and their subsequent reactivity. Given the high number of variables, we use the term “chemical landscape” to indicate the ensemble of chemicals (molecules and particles) present on the surface of a given leaf in a specific moment of time. The complex relationships between surface properties, multiphase chemistry, and surface–air exchange are more established for urban grime^24−27^ and pesticide loss on leaf and soil surfaces^28−31^ but have yet to be thoroughly explored in terms of plant surfaces.

In this Review, we provide an evidence-based overview of the chemical compounds most likely to be found on leaf surfaces under natural conditions and describe their known and expected reactivity with atmospheric oxidants. Due to the large number of plant species on Earth, the broad diversity of metabolites, and the high variability among species and, for the same species, among individual plants, we concentrate on compound classes rather than individual molecules, and on organic rather than inorganic compounds. For simplicity, we also generally limit our discussion on intact, nonsenescing leaves of vascular plants, namely, angiosperms (broadleaves) and gymnosperms (conifers), even though many aspects are applicable to ferns, bryophytes, other aerial plant surfaces (e.g., branches, fruits, flowers, and cork), and dead leaves.

The Review is organized into four parts. We first provide a general overview of basic anatomical aspects of leaves (section 2.1) and discuss morphology and chemical composition of the cuticle (section 2.2). Second, we describe the organic compounds that have been detected on leaf surfaces and discuss their observed or potential reactivity. These molecules can be *endogenous*, i.e., plant-derived (section 3), or *exogenous*, i.e., deposited from the environment via wet, dry, or mixed-type pathways (section 4). Third, we discuss the effect of water films and drops on cuticles and on the fate of leaf-adsorbed compounds (section 5). Fourth, we provide a semiquantitative estimate of the classes of organic compounds most likely to be found on leaf surfaces (section 6.1) and a unified overview of the dynamic leaf surface reactivity (section 6.2). We hope this Review will provide new insights into the role of leaves in multiphase atmospheric processes and spur new research into how this system impacts climate, air quality, and ecosystem health.

## 2 Leaf Surface

### 2.1 General Anatomy of Leaves and Secretory Structures

**Figure 1:**
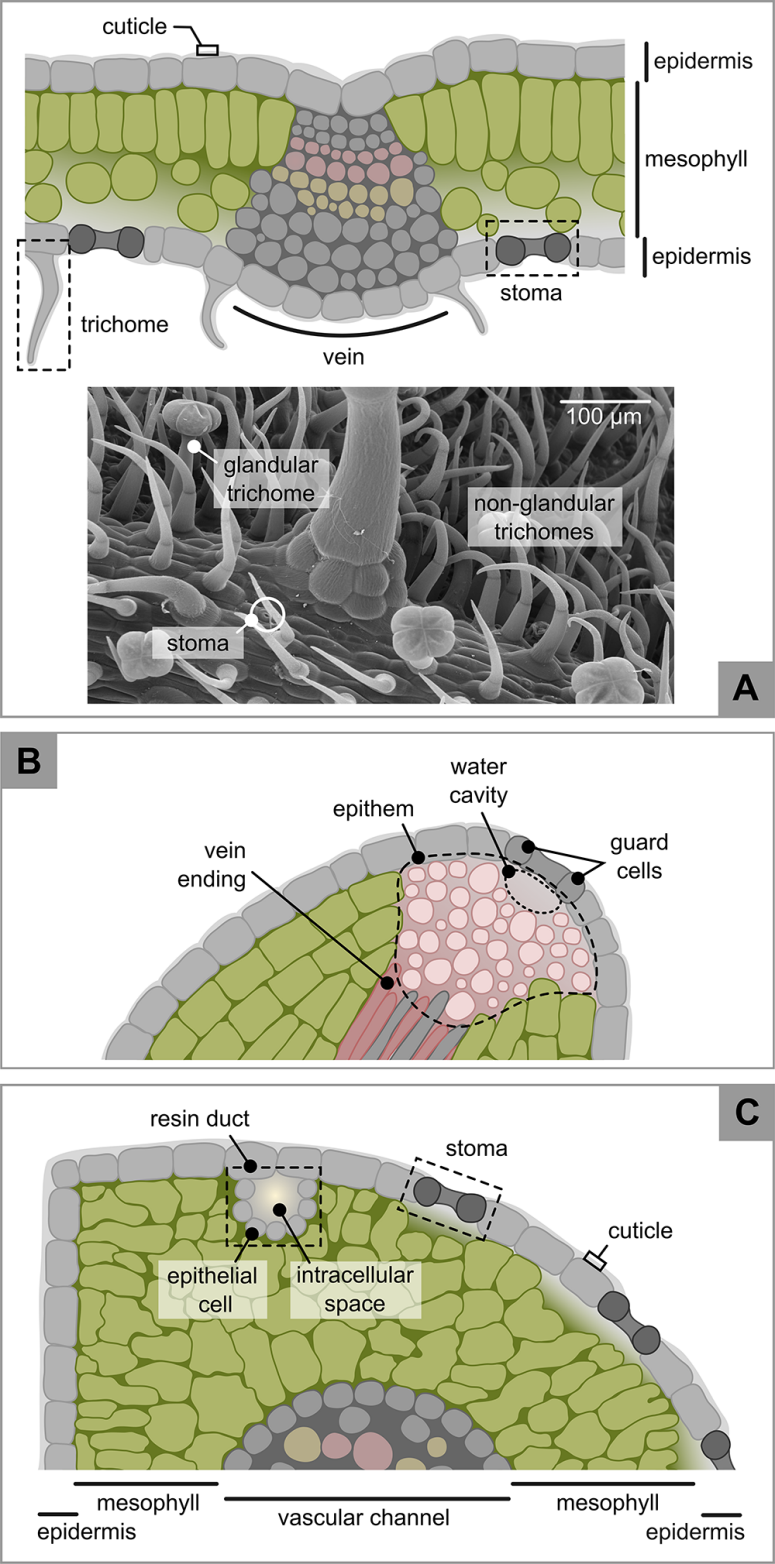
Pictorial representation of angiosperm (A) and gymnosperm (C) leaf sections inspired from photographs of tomato^41,42^ (*Solanum lycopersum*) and mountain pine^43^ (*Pino mugo*) leaves, respectively. Panel A includes a scanning electron microscopy photograph of a tomato leaf with highlighted glandular and nonglandular trichomes (type VI and II, respectively, according to the Luckwill’s classification scheme^44^). Panel B illustrates hydathodes, additional structures present in most vascular plants. Micrograph courtesy of M. J. Guinel de France (Dartmouth Electron Microscope Facility).

The leaves of almost all vascular plants share a similar microscopic structure consisting of an external layer of cells, the epidermis, that encloses the mesophyll, the inner leaf region (Figure 1).^32−34^ Stomata, pores that allow gas exchange between the atmosphere and the mesophyll, are also located within the epidermis, either on one or both sides of the leaf depending on the species. The entire epidermis, including eventual surface structures, is covered by a thin waxy layer (0.03 to 30 μm)^35,36^ called the cuticle. Besides these general features, leaves of angiosperms and gymnosperms host specific structures involved in the production, storage, and excretion of primary and secondary metabolites that include trichomes, hydathodes, and resin ducts (Figure 1).^37^ Other secretory structures not described here are latex ducts, gum ducts, salt glands, nectaries, and resin cavities.^37,38^ Thus, to cite Morris, the leaf surfaces are not smooth and featureless but rather are “comparable to a forested island whose surface is replete by burrows and tunnels”.^39^ The presence of “a forest” of microscopic structures is crucial in defining type and amount of organic compounds that can be found on leaf surfaces and their interaction with the atmosphere–especially if considering that trichomes can increase the effective leaf area up to 4 orders of magnitude.^40^

#### 2.1.1 Trichomes

All aerial organs of angiosperms, including leaves, can be covered with trichomes, external appendices that range from a few μm to several cm and provide a protective barrier toward abiotic and biotic stressors (Figure 1A).^45−48^ Trichomes consist of one or more epidermal cells growing on top of the leaf epidermis and are generally independent from the plant’s vascular system.^32^ Several types of trichomes exist,^49^ but they are typically divided into glandular and nonglandular.^46^ Glandular trichomes have an active secondary metabolism and are often considered the plant’s factory of specialized chemicals. The structure of glandular trichomes is dictated by the volatility of their major metabolite.^46,47^ Volatile metabolites are often produced and stored in peltate or biseriate trichomes, whereas less volatile substances are typically associated with capitate trichomes, which are characterized by one or several secretory cells at the top of a stalk (Figure 1A).^46,47,50^ The specific chemicals produced by glandular trichomes are described in section 3.1. Nonglandular trichomes lack secretory structures and primarily provide mechanical protection to biotic and abiotic stressors.^50^ In broadleaves, the type of trichomes vary from species to species, with densities ranging from 1 to 250 mm^–2^ and 1 to 140 mm^–2^ for nonglandular and glandular trichomes, respectively (Li et al.^50^ and references therein). To our knowledge, trichomes in gymnosperms have only been observed on the stem and needle surfaces of young conifer shoots.^51^

#### 2.1.2 Hydathodes

Most vascular plants host specialized structures on the surface, margins, and tips of leaves called hydathodes.^37,52,53^ Hydathodes consist of an area of loosely packed cells (the epithem) at the end of the leaf’s vein, a water cavity, and two guard cells that always remain open (Figure 1B).^52,54,55^ When root water uptake is favored and leaf evapotranspiration is disfavored—e.g., during cold nights or predawn following warm days, or in tropical wet climates—the plant’s sap may be excreted through the hydathodes, a process known as guttation.^52,53,55,56^ If relative humidity is high, guttation droplets remain visible along leaf margins and surfaces, whereas in dry conditions, water evaporates leaving a solid residue. In a single guttation event, plants can secrete from a few drops to several milliliters of guttation fluids.^53,57^ (The chemical composition of these fluids is discussed in section 3.2.) Although hydathodes are widespread among vascular plants, guttation requires unique meteorological conditions and is rarely observed in woody plants of temperate climates.^52^

#### 2.1.3 Resin Ducts

Gymnosperms have additional structures dedicated to the synthesis and storage of resin that include resin ducts, resin cavities, and resin cells.^37,43,58^ Resin ducts are mostly found in needles, cortex, xylem, and phloem of the *Pinaceae* family, including spruces (*Picea spp*.) and pines (*Pinus spp*.).^43,58^ In needles, resin ducts are situated in the mesophyll and consist of an intracellular space surrounded by epithelial cells (Figure 1C). The latter produce and excrete resin into the duct, where it accumulates. Within the mesophyll, resin ducts can be along the needle margin, in its central portion, or both, and are always at least two per needle.^43^ Details on the chemistry of resins are in section 3.3.

### 2.2 Leaf Cuticles

#### 2.2.1 Chemistry and Morphology

Cuticles cover all aerial parts of plants; their thickness, chemistry, and morphology differ depending on plant species, organ, developmental stage, and climatic factors.^48,59−62^ Additional variables impacting the cuticle’s structure include epidermal cell type, leaf age, light exposure, and the presence of phyllosphere microorganisms, among other things.^61^

**Figure 2:**
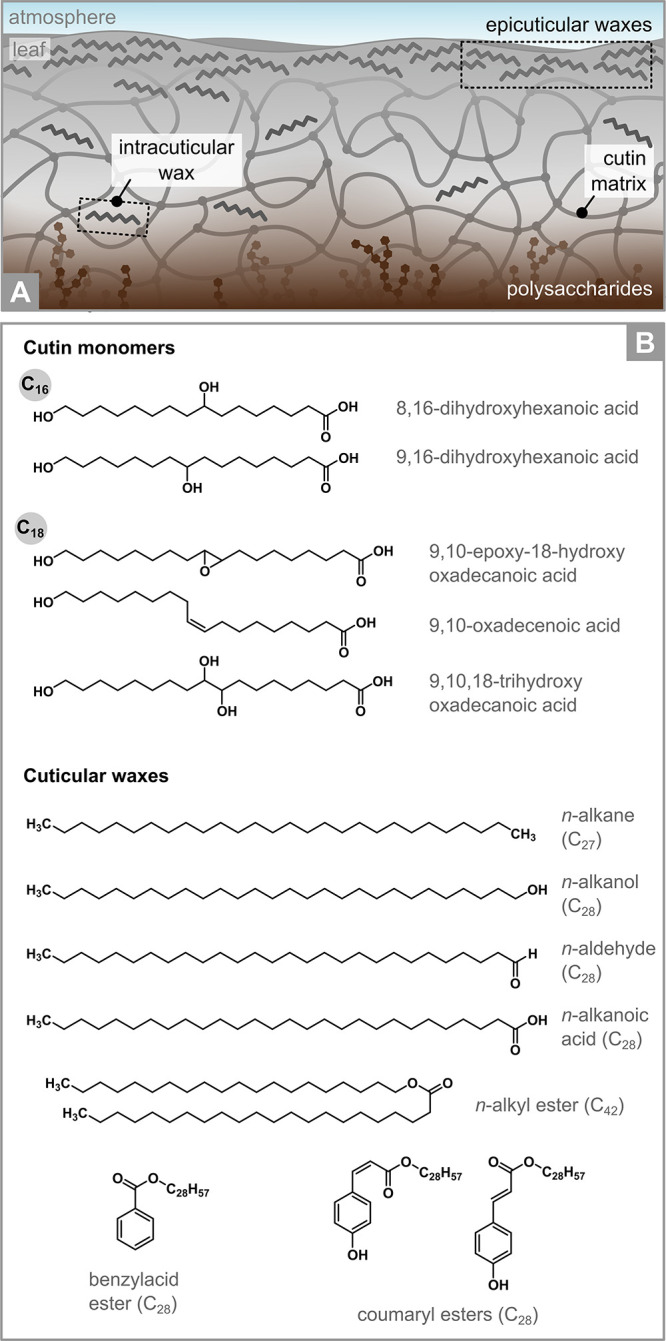
Chemical composition of leaf cuticles. (A) Schematic representation of a cuticle showing the location of its primary components. (B) Major chemicals in the leaf cuticles of *Fagus sylvatica* (European beech).^63−65^ Epi- and intracuticular waxes are reported together, although the distribution of each chemical may vary depending on its location within the cuticle (see Supporting Information for additional details).

Cuticles consist of two key ingredients: cutin and waxes (Figure 2).^35,36,66−68^ Cutin is an amorphous polyester of C_16_ and C_18_ ω-hydroxycarboxylic acids, both of which can have midchain hydroxy, epoxy, and carbonyl functionalities that act as polymer branching points or hydrophilic sites. C_16_ acids are typically present in large amounts in every plant species, while the specific substitution pattern and relative contribution of C_18_ acids varies. In some plants, cutin contains small amounts of glycerol.^66,69,70^ Overall, the amount of cutin ranges from a few μg cm^–2^ to up to 1 mg cm^–2^,^36^ corresponding to 40–80% of the total cuticle’s mass.^32,36^

Waxes are the second most abundant components of leaf cuticles by mass and are mixtures of compounds including long-chain fatty acids, *n-*alcohols, *n*-alkanes, *n*-aldehydes, alkyl esters, and triterpenoids.^67^ The exact blend of molecules and their tridimensional arrangement are characteristic of each plant species and plant organ (see Jetter et al.^67^ for details). Depending on their location within the cuticle, waxes are intra- or epicuticular (Figure 2A). Intracuticular waxes are embedded within the cuticle’s polymer matrix, where they help strengthening its structure and prevent water loss, whereas epicuticular waxes are deposited on the top of cutin, effectively representing the outermost layer of the plant.^68^ Epicuticular waxes display a large variety of tridimensional structures, textures, and patterns that create a heterogeneous surface at the submicron scale.^71−73^ They can be amorphous or crystalline, with the crystal morphology being broadly controlled by their chemical composition.^73−75^ Epicuticular wax morphology is also influenced by leaf age and environmental factors like temperature, pollution, water availability, salinity, solar exposure, and mechanical stress (see also section 2.2.2).^74,75^ Although the mechanisms responsible for the formation of these many epicuticular wax patterns are not yet fully understood, their presence impacts the leaves’ interaction with water, particles, and organic molecules, in addition to their mechanical and optical properties.^71^

Additional compounds can be found in minor quantities in leaf cuticles. Polysaccharides from the epidermal cell wall are present in the lower part of the cuticle, where they play a pivotal role in the absorption and transport of water and hydrophilic molecules (Figure 2A; see also section 5.2).^36,76^ Cutan is the insoluble residue that is found after depolymerization of cutin in some plant species.^76,77^ Cutan is understood to be a polymer of long-chain aliphatic moieties and a small fraction of hydroxylated aromatics cross-linked through ester and ether bonds, although its origin and structure are under debate.^77^ Phenolic compounds like cinnamic acids, flavonoids, flavonols, and, in some gymnosperms, lignin-like moieties are also present either free or copolymerized within the cutin matrix, where they help protect the leaf against ultraviolet radiation.^36,68,78^ In some plant species (e.g., *Fagus sylvatica*; see Figure 2B), alkyl hydroxycinnamates are also present in association with cuticular waxes.^79^ Cuticles also contain water in both “free” (i.e., in equilibrium with the gas-phase) and “embedded” forms (i.e., having two or three hydrogen bonds with polar cuticle components).^80^ The amount of water depends on ambient relative humidity and temperature, among other factors, and impacts the cuticle’s mechanical properties (see also section 5.2).^81^

#### 2.2.2 Reactivity

Given their location at the plant-atmosphere interface, cuticular components—and epicuticular waxes in particular—have the potential to engage in multiphase reactions with gas-phase oxidants. Although this hypothesis has been circulating since the 1980s, empirical evidence for a “pure” chemical reactivity remains scarce.^67,75^ In conifers, this lack of reactivity has been attributed to the peculiar chemistry of their epicuticular waxes—they primarily consist of 10-nonacosanol, a saturated alcohol unreactive toward O_3_.^75^ Indeed, Jetter et al.^82^ showed that ambient levels of NO_2_, SO_2_, and O_3_ do not impact the chemical composition of epicuticular waxes isolated from *Picea pungens*, although oxidation products were detected after unrealistically high NO_2_ exposure (i.e., equivalent to 700–58,000 years at ambient levels). Exposure to pollutants can induce chemical changes in epicuticular wax composition in both broadleaves and conifers;^74,83,84^ however, variation in plant metabolism rather than direct chemical reactivity is so far the most convincing explanation for this phenomenon.^83,85^

Conversely, there is ample evidence that exposure to atmospheric oxidants impacts epicuticular wax *morphology* by accelerating its natural weathering (reviewed by Turunen and Huttunen^86^ and others^74,75,87^). In conifers, the most common symptom is an accelerated fusion of wax tubes around stomata.^86,87^ This process occurs naturally as the needle ages, but it is accelerated after exposure to acid rain or mist, SO_2_, and/or NO_2_.^86,87^ Exposure to O_3_ alone did not consistently induce morphological changes but had an effect when present in combination with other oxidants.^87^ Various explanations have been put forward to justify these morphological variations, with changes in wax biosynthesis being the most likely.^75,87^ More recently, Burkhardt et al.^88,89^ showed that deliquescent aerosol particles on leaf surfaces look visually similar to degraded waxes, suggesting that metabolic changes are not the underlying cause of this phenomenon.

Despite general agreement about the lack of *direct* chemical reactivity, there are a few biases worth highlighting. First, most studies were performed between the beginning of the 1970s and the end of the 1990s, and visually detected changes in wax crystal morphology via microscopy. When chemical analyses were performed, instrument sensitivity might not have been sufficient to detect minor variations in wax chemistry. Second, the literature is heavily biased toward conifers,^75,86,87^ whose epicuticular waxes consist primarily of 10-nonacosanol, a saturated alcohol.^75^ However, other plant species may have more reactive molecules in their leaf cuticles. For example, more than 50% of the fatty acids in the cuticles of some mangrove species are unsaturated,^90^ while *Fagus sylvatica* and other plants have traces of alkyl coumarates and other unsaturated compounds (Figure 2B).^64,79^ Notably, an early study reported significant production of 4-oxopentanal, 6-methyl-5-hepten-2-one, and geranyl acetone upon ozonation of isolated cuticles of various oak species (e.g., *Quercus ilex* and *Quercus suber*) and other common Mediterranean plants.^91^ Although the authors did not investigate the nature and location of the parent molecule(s), some *Quercus* species show a remarkably high proportion of terpenes and terpenoids in their cuticle, in addition to a few aromatic compounds.^92,93^ (However, metabolites excreted from glandular trichomes^94,95^ (section 3.1) and/or sampling artifacts (i.e., skin lipids^91^) may also be responsible for the reactivity observed in Fruekilde et al.^91^) Third, physically damaged leaves may expose chemicals embedded within the cuticle to atmospheric oxidants—e.g., phenolics in the cuticle^36,79^ and in other leaf tissues^79^ may react with gas-phase oxidants.^96−99^ In conclusion, the cuticle’s reactivity may warrant a reassessment, as it may be relevant in damaged leaves and in plant species with traces of unsaturated chemicals in their leaf cuticle.

## 3 Chemicals from the Plant and Its Biome

Most leaf surface structures excrete chemicals—glandular trichomes exude a rich variety of secondary metabolites (section 3.1.1), hydathodes release drops of the plant’s sap (section 3.2.1), and resin ducts excrete resins (section 3.3.1). However, not all plant species display these structures; and when present, environmental conditions influence their role as a source of chemicals. As such, we expect the blend of endogenous compounds to be highly species-specific and, to a certain extent, predictable. Furthermore, endogenous exudates are typically highly concentrated and have thus high potential to be involved in multiphase reactions—though, to our knowledge, this reactivity has only been marginally explored for trichome metabolites of a few plant species (section 3.2.1).

If one considers plants as miniature ecosystems, the definition of “endogenous” can be broadened to include chemicals and particles from organisms that inhabit the canopy. Section 3.4 provides a brief overview of the community of microbes living on leaf surfaces and its direct and indirect influences on the chemical landscape’s chemistry and reactivity. Other organisms that may contribute additional species (but are not discussed) include epiphytes (reviewed by Van Stan and Pypker^100^) as well as insects, little invertebrates, and macrofauna (reviewed by Ponette-González et al.^101^). Depending on their source, pollen and fungal spores may be considered endogenous or exogenous; in this work, we assume the latter and cover them in section 4.

### 3.1 Glandular Trichomes

#### 3.1.1 Chemicals

Glandular trichomes produce and/or accumulate a rich variety of metabolites (reviewed by Schilmiller et al.^102^ and others^103,104^) in amounts that can reach up to 30% of the leaf’s dry weight.^46,103^ Terpenes are among the most common compounds stored in glandular trichomes, including monoterpenes (C_5_H_16_), sesquiterpenes (C_15_H_24_), diterpenes (C_20_H_32_), and terpenoids resulting from oxidation, conjugation, or other structural modification of the former. While monoterpenes are markedly volatile,^105^ members of other classes are less prone to gas-phase partitioning and will (at least partially) remain on the leaf’s surface—e.g., in the form of drops either accumulated onto the head or dripping along the stalk of capitate trichomes.^46,47,103^

Phenylpropanols are other common secondary metabolites produced in glandular trichomes,^102^ whose basic structure consists of a C_3_ chain linked to an aromatic ring.^106^ Some examples include chavicol, methyl chavicol, eugenol, and methyl eugenol, volatile compounds produced by the peltate trichomes of some basil varieties^107^ and other plant species.^108^ Flavonoids and other polyketides are other important metabolites produced in these structures and often found embedded into the cuticle.^109^ Glandular trichomes can also produce and excrete fatty acids and antimicrobial proteins. For example, tobacco trichomes actively secrete T-phylloplanins, small water-soluble glycoproteins that can inhibit the germination of fungal spores causing the blue-mold disease.^110,111^ In general, the specific suite of chemicals in glandular trichomes is unique of each plant taxa and can be used as a classifying tool (see Spring^112^ for details). For a few plant species of high economical or medicinal relevance, reviews exist on the entire suite of secondary metabolites produced by their glands (e.g., tobacco,^111^*Cannabis sativa*,^113^ and *Artemisia annua*(114)).

#### 3.1.2 Reactivity

**Figure 3:**
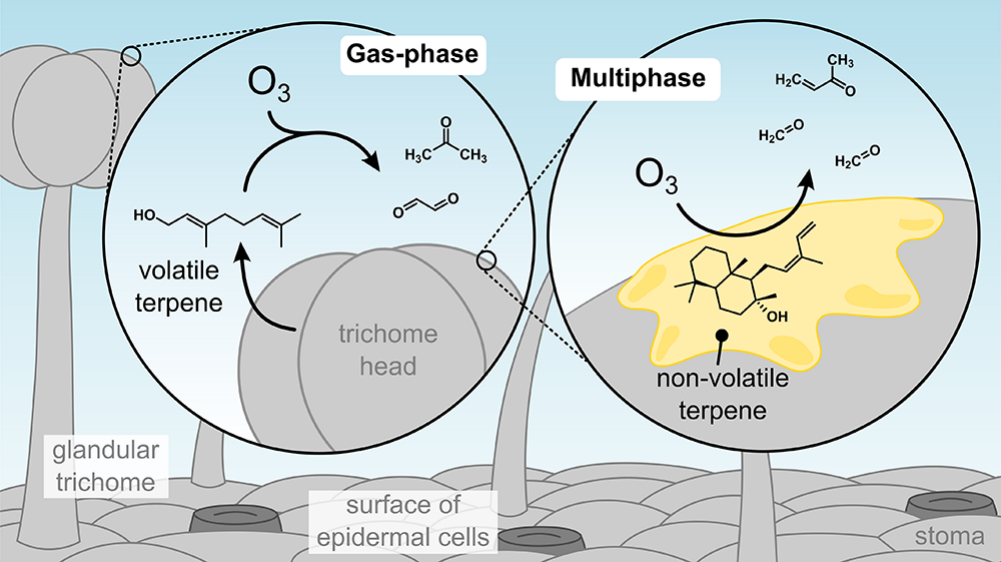
Examples of gas-phase (left) and multiphase (right) reactions involving glandular trichome’s metabolites. On the left, geraniol, a volatile monoterpenoid produced by several plant species, undergoes gas-phase reactivity with ozone to produce acetone and glyoxalate.^125^ On the right, *cis*-abienol, a nonvolatile diterpenoid produced by some tobacco varieties, reacts with gas-phase ozone to yield formaldehyde and methyl vinyl ketone.^120^ Although multiphase reactions are considerably less studied than gas-phase processes, both have been observed experimentally.

Trichome metabolites have a wide range of volatility and reactivity, and consequently have varied impacts on atmospheric chemistry. Monoterpenes are volatile and contribute to gas-phase reactivity in the atmosphere; this chemistry has been extensively studied due to the relative ease of detection and monoterpene’s potential for gas-phase oxidation (e.g., Figure 3, left).^115−119^ In contrast, systematic investigations of multiphase reactions involving non- or semivolatile metabolites are rare. Jud et al.^120^ showed that *cis*-abienol, a diterpenoid produced by the trichomes of some tobacco varieties, readily reacts with ozone to produce gas-phase formaldehyde and methyl vinyl ketone (Figure 3, right), whereas plant varieties that excrete cembradientriol, another diterpenoid, produced 4-oxapentanal. A follow-up study showed that the density of glandular trichomes, capitate trichomes in particular, strongly correlates with nonstomatal O_3_ uptake also in other woody and herbaceous plants.^50^

In addition to ozone, Khaled et al.^121^ showed that myrigalone A, a nonvolatile secondary metabolite of the Mediterranean bush *Myrica gale*, can be degraded by sunlight to yield acetic acid, benzaldehyde, and other gas-phase products. Although these experiments were performed on model surfaces, real-leaf extracts contained myrigalone A’s photodegradation products, suggesting that this process occurs also under natural settings.

Thus, given the substantial reactivity of several low-volatility terpenes and terpenoids toward O_3_ and hydroxyl radicals^122,123^ and the light-absorption properties of some secondary metabolites,^121,124^ multiphase oxidation involving semi- and nonvolatile chemicals from trichomes may be an overlooked source of low-molecular-weight compounds (e.g., formaldehyde^120^ or formic acid^118^) and a sink of reactive trace gases in specific plant species.

### 3.2 Guttation Fluids

#### 3.2.1 Chemical Composition

Guttation droplets contain a wide variety of organic and inorganic compounds, whose identity and concentration depend on plant species, age, physiological activity, and the chemistry of the plant growing medium.^126^ The most common organics in guttation fluids are primary plant metabolites, i.e., sugars, amino acids, proteins, enzymes, nucleotides and nucleic acids, plant hormones, and alkaloids, in addition to secondary metabolites like monoterpenes and sesquiterpenes.^52,126^ Concentrations vary by orders of magnitude but are always substantial. For example, sugars can range from 27 mg L^–1^ to 1500 g L^–1^, while proteins can be between 2.7 mg L^–1^ and 30 g L^–1^ (reviewed by Urbaneja-Bernat et al.^127^). Systemic pesticides such as neonicotinoids have also been found in crops’ guttates in tens of mg L^–1^ (summarized by Thompson^128^). In addition to organic compounds, guttation fluids contain a wide range of inorganic species whose identity and concentration depend on the chemistry of the plant growing medium (i.e., soil or nutrient solution).^52,126^

#### 3.2.2 Reactivity

The extent to which chemicals in guttation fluids take part in multiphase chemistry is currently unconstrained by observations. Dibley et al.^129^ speculated that guttation may contribute to the pool of organics detected in dew droplets and frost collected from grass blades, but did not provide conclusive evidence for their occurrence. This mixture of compounds was further shown to be susceptible to photodegradation (in bulk aqueous solutions),^129^ hinting that components of guttation fluids may take part in photochemical reactions when co-occurring with other water-soluble leaf surface chemicals.

Although solute concentrations can be significant, the unique conditions required for guttation to occur combined with the relatively small volumes of liquid excreted in each event (section 2.1.2) suggests that these chemicals may be relevant to the overall leaf surface reactivity only in selected environments, seasons, plant species, and under specific weather conditions.

### 3.3 Resin Ducts

#### 3.3.1 Chemicals

**Figure 4:**
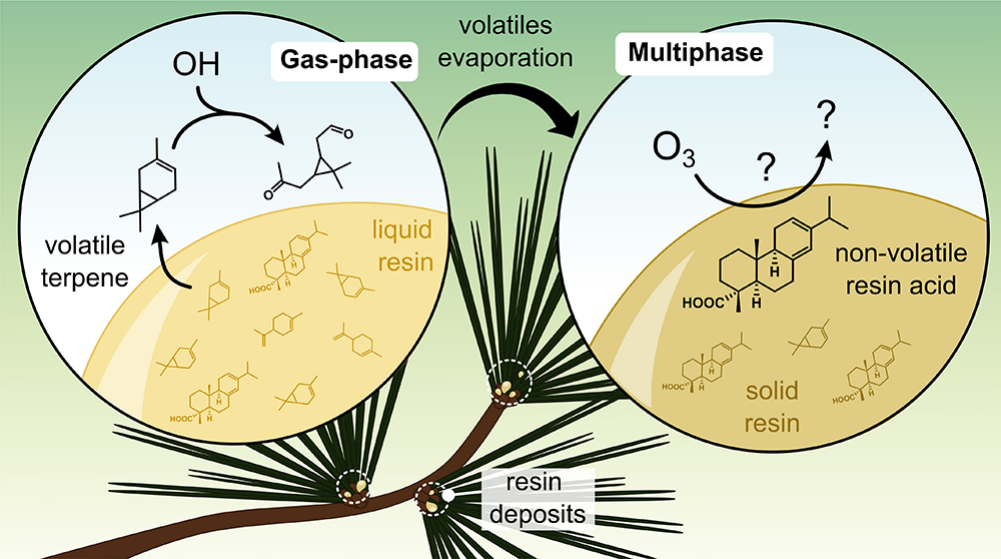
Examples of gas-phase (left) and multiphase (right) reactions involving resin components. On the left, Δ^3^-carene, the most abundant volatile in the sapwood resin of *Pinus ponderosa*,^138^ undergoes hydroxyl radical (OH) oxidation to form caronaldehyde.^116,139^ The right panel depicts the potential ozonation of levopimaric acid, the primary resin acid in *Pinus ponderosa* sapwood resin.^138^ This multiphase reactivity has not yet been reported under natural conditions but is likely to occur based on literature precedents.^137^ This figure also illustrates the increasing contribution of multiphase reactivity as just excreted resins dry to form solid or semisolid deposits.

Resins are mixtures of terpenoids consisting of a volatile and a nonvolatile fraction.^43,130,131^ Volatile chemicals include monoterpenes and some sesquiterpenes, and their relative proportion controls the overall viscosity of the resin. The nonvolatile fraction consists mostly of diterpene and triterpene acids such as abietic acid.^130,131^ Short-chain alkanes are also found in resins of some pine species.^132^ Resins are part of the plant’s defense mechanism and typically exit resin ducts because of needle damage—e.g., following a wound induced by beetles.^133^ Resins can also extrude without damage at the junction between needles and the branch and on seed cones, especially in the spring.^133^ After resin excretion, the volatile fraction evaporates leaving a solid residue on the needle that protects the damaged site both physically and chemically (Figure 4).^58,131^ For the same plant species, the resin’s chemical composition can vary from organ to organ (e.g., needles vs bark).^43^

#### 3.3.2 Reactivity

Even though several authors have shown that volatile resin components can impact atmospheric chemistry in sporadic, local episodes (e.g., Jaakkola et al.^134^ and others;^133,135^Figure 4, left), the interest in the nonvolatile fraction has so far been limited. However, empirical evidence from other fields suggests that also these chemicals react with atmospheric oxidants. For example, a few studies investigating the stability of biomass burning tracers showed that abietic acid undergoes heterogeneous reactions with hydroxyl radicals, nitrate radicals, and ozone (summarized by Arangio et al.^136^). Oxidation of abietic and other diterpenoid acids ubiquitous in resins of the *Pinacea* family occurs also in varnish covering paintings and other artwork,^137^ suggesting the same may take place on the surface of resin residues on needles and other plant surfaces (Figure 4, right).

### 3.4 Phyllosphere Microorganisms

#### 3.4.1 Chemicals

Despite the extreme conditions caused by lack of nutrients, intermittent water availability, intense solar radiation, and fluctuating temperatures, leaf surfaces host microbial communities collectively referred to as “phyllosphere” microorganisms.^39,61,140,141^ This term includes primarily bacteria (mainly *Proteobacteria*) and fungi (mainly *Ascomycota* and *Basidiomycete* yeasts), while archeobacteria, algae, nematodes, and viruses are less common.^1,140^ To increase their chance of success, phyllosphere microorganisms form biofilms and live preferentially in the most protected regions of the downward-facing leaf side (e.g., around trichome bases, in the indentations between epidermal cells, and inside stomata).^61^ Although phyllosphere microorganisms are ubiquitous (with an average surface density of 10^6^–10^7^ cells cm^–2^; see refs in Lindow and Brandl^142^), they are estimated to occupy less than 2% of the available global leaf surface area.^39^ Leaf surface microbiology is a complex and active field of research; readers interested in this topic can find more information in dedicated reviews and book chapters (e.g., Vorholt,^1^ Trivedi et al.,^143^ and others^39,61,140−142^).

Phyllosphere microorganisms release a variety of chemicals onto leaf surfaces to increase their chance of survival. Important classes include extracellular polymeric substances (EPS), surfactants, and plant hormones.^1,61^ EPS are major biofilm components on a per-mass basis (50–90%)^144,145^ and are crucial to keeping bacterial cells hydrated.^1^ This polymeric matrix has a complex and dynamic composition and includes functionalized polysaccharides, proteins, nucleic acids, phospholipids, and traces of humic substances.^145−147^ Surfactants help cells access water and nutrients either by softening the cuticle (see also section 4.2.2), reducing water tension (thus, allowing bacteria to relocate where nutrients are more abundant), or increasing water availability.^61,148,149^ For example, syringafactin, a hygroscopic surfactant produced by *Pseudomonas syringae*, can absorb water up to 250% of its weight at high relative humidity.^149^ Some phyllosphere organisms also produce volatile organic compounds (reviewed by Farré-Armengol et al.^150^) and auxin, a nonvolatile plant hormone that stimulates the release of saccharides from the plant cell wall and helps alleviate nutrient limitations.^1^

#### 3.4.2 Reactivity

To the best of our knowledge, no studies have explicitly investigated the potential for multiphase reactions of phyllosphere-derived compounds with atmospheric oxidants–although results from other fields hint they may occur. For example, aqueous solutions containing EPS isolated from pure microbial cultures are susceptible to photochemical reactions,^151^ hydroxyl radical oxidation,^152^ and ozonation,^153^ as are suspension of bacterial cells and other pathogens.^154−156^

In addition to reactions involving specific biofilm components, phyllosphere microbes can impact the leaf surface’s chemical composition and reactivity in other ways (see also Farré-Armengol et al.^150^). First, by creating and maintaining a layer of microscopic wetness, these microorganisms can facilitate the leaching of organic and inorganic substances to the leaf surface (see also section 5). Microbial surfactants similarly enable the leaching of endogenous compounds. Second, microbes can take up or modify adsorbed pollutants or specific plant metabolites. This process has primarily been described for natural compounds (e.g., methanol^157^ and monoterpenes like geraniol and nerol^158^) but anthropogenic chemicals can undergo a similar fate (e.g., phenol^159^ and polycyclic aromatic hydrocarbons (PAHs); summarized by Terzaghi et al.^160^). In addition to biological processes, abiotic reactions mediated by redox-active EPS moieties (e.g., proteins with a sulfhydryl group) and extracellular enzymes can also occur within the biofilm.^145,146^ Third, pathogenic microorganisms can induce the host plant to produce and release specific chemicals that otherwise would not be present.^150^ Elucidating the full range of reactions enabled by phyllosphere biofilms remains a major topic of future (interdisciplinary) investigations.

## 4 Chemicals from the Environment

**Figure 5:**
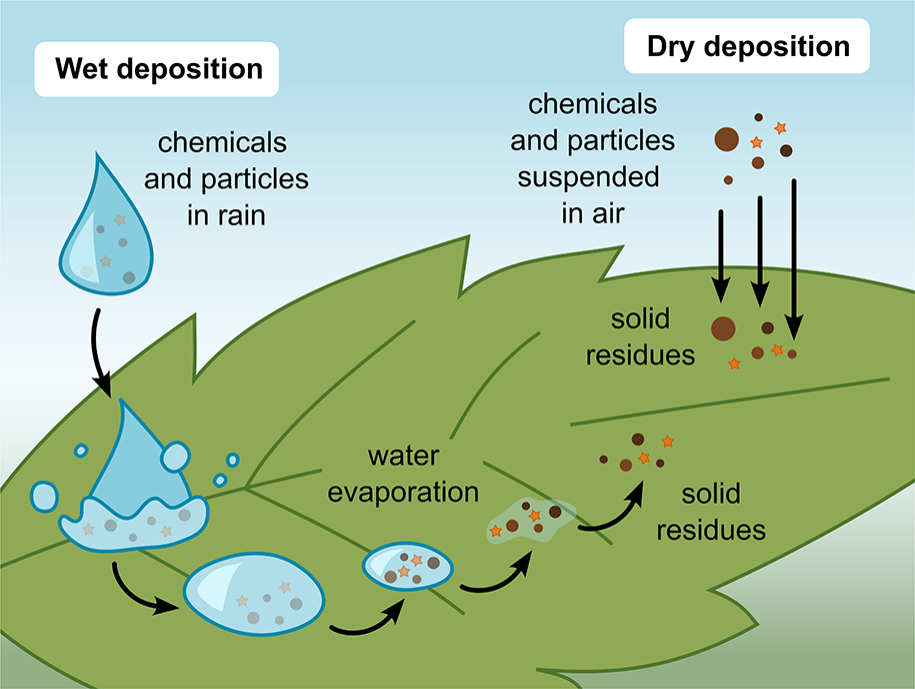
Overview of the main delivery pathways of exogenous substances onto leaf surfaces. For wet deposition to contribute leaf surface material, drops must remain on the leaf—if drops roll off, they are more likely to remove soluble material and particles from the leaf surface. Particles are indicated as brown circles, while water-soluble chemicals and semivolatile compounds are depicted as orange stars.

In addition to metabolites produced by the plant and its associated biome, compounds from the surrounding environment can find their way onto leaves via various pathways (Figure 5). Dry deposition describes the direct delivery of mass via gravitational settling, impaction, interception, diffusion, and adsorption (section 4.1), whereas in wet deposition, chemicals and particles reach plant surfaces through precipitation or other forms of liquid media (section 4.2).^161−163^ For specific types of particles, mixed forms of deposition are also possible (section 4.3).

Based on our critical analysis of the literature, dry deposition appears responsible for delivering most exogenous substances onto leaf surfaces, including particles (sections 4.1.1–4.1.3) and semivolatile compounds (section 4.1.4). The role of wet deposition is more challenging to estimate due to the polyvalent role of water in impacting the leaf’s chemical landscape (see also section 5), the overall dilute character of hydrometeors, and case-by-case differences in meteorological conditions, geographical location, and plant species (section 4.2.1). Radionuclide studies indicate that rain can deliver particles and solutes onto leaf surfaces, but large variations exist depending on the plant development stage, leaf characteristics, rain amount, and chemistry of the deposited material (section 4.2.1.1). Foliar application of pesticides is another wet deposition pathway discussed in the literature relevant primarily to agricultural settings (section 4.2.2). Irrespective of their delivery pathway, exogenous substances are susceptible to multiphase reactions. Although empirical data is available only for a few groups of anthropogenic semivolatile compounds and primarily for photochemical reactions (section 4.4.1), knowledge from the broad environmental chemistry literature strongly suggests that leaf surface reactivity of exogenous chemicals with atmospheric oxidants is widespread (section 4.4.2).

### 4.1 Dry Deposition

#### 4.1.1 Particulate Matter

*A rich body of literature supports the ability of leaves to intercept atmospheric particulate matter (PM) in various size ranges.* A recent meta-analysis indicated that plants growing in urban environments capture 50–550 μg cm^–2^ week^–1^,^164^ resulting in surface concentrations ranging from 1 to 191 μg cm^–2^ (Figure S1).^165,166^ These numbers vary as a function of leaf structure, meteorological, and geographical factors as well as particles’ concentration, size, and morphology.^165^ Overall, leaf roughness and hairiness (i.e., the type and surface concentration of trichomes) are the two most important factors defining leaves’ ability to intercept and retain PM, with rough, trichome-rich leaves being more effective than smooth leaves void of trichomes.^165,167^ Furthermore, conifers have been found to accumulate overall more PM than broadleaf species, likely because their leaves persist throughout the year.^164,165,168^ In addition to leaf traits, PM loads depend on a plant’s proximity to emission sources (e.g., industries or streets) and the presence and frequency of removal processes, which include rain wash-off and resuspension by wind. Rain can remove 30–70% of accumulated PM, with differences based on leaf type (broadleaves, 51–70%; needles, 30–41%) and particulate matter size. In general, the longer the accumulation period (i.e., days after rain and leaf age), the higher the PM mass on leaves.^164^ Furthermore, fine particulate matter (PM_2.5_, particles with diameter <2.5 μm) is generally more effectively retained on leaves than the coarse fraction (PM_10_, particles below 10 μm), as its smaller size allows a more efficient capture by surface grooves, ridges, and scales, and a partial encapsulation in surface waxes.^166,167,169^ Particles larger than 50–100 μm are generally not observed on leaves.^163^

Although it is well-known that aerosols can be deposited onto any type of vegetation (e.g., Petroff et al.^170^) and that this deposition is linked to the plant traits and deposited PM mass, there is limited knowledge on the chemical composition of leaf-deposited PM—primarily because of methodological biases. Most studies are based on gravimetric methods and/or electron microscopy, which only provide bulk mass and elemental composition (i.e., percent of carbon or metals).^166,171^ In addition, the most common gravimetric method quantifies only the insoluble fraction. The few groups analyzing both insoluble and water-soluble components consistently showed that inorganic ions contribute on average to 30% (range = 7–50%) of the total deposited mass,^168,172^ hinting that most studies underestimate total deposited PM. Water-soluble organics from leaf-deposited PM are challenging to quantify via bulk analyses because of the matrix of organic compounds that is naturally present on leaves.^173^ To our knowledge, a thorough chemical characterization of the water-soluble organic fraction has not yet been performed—although traces of aromatics (possibly originating from the partial dissolution of biomass burning aerosols) were detected in dissolved organic matter collected below wet tree crowns (see section 5.3).^174^

Although their chemistry is not thoroughly characterized, microscopy images provided visual evidence for the presence of specific types of particles, including pollen, soil-derived PM, spores, bacteria, combustion products, metallic particles, and aggregates (e.g., Freer-Smith et al.^175^). The occurrence of pollen and soil-derived PM on leaf surfaces has been further investigated in crop or pasture systems and is illustrated more in detail in the following sections. In particular environments, mineral dust (e.g., cement dust) can also accumulate on leaves (reviewed by Burkhardt and Grantz^166^).

#### 4.1.2 Pollen

Pollen is a specific type of particulate matter that has been detected on leaves, representing both an endogenous and exogenous source of chemicals. In general, pollen can be <10 to >100 μm in size,^176^ averaging 15–60 μm in species relying on wind pollination.^177,178^ Pollen size and morphology are unique in every plant species,^176^ which helps distinguish it from other particles and identify its source.^175^ The large size limits the relevance of intact pollen deposition to the local scale (typically within meters to hundreds of kilometers from its emission source),^179^ with variations based on meteorological conditions, as well as grain size and shape.^179,180^

In general, pollen dynamics follow the same trends as PM deposition. First, not all pollen grains suspended in the air stick onto leaves, with percentages that vary depending on the presence and density of trichomes, leaf orientation, area, and position within the plant (e.g., Pleasants et al.^181^). Second, rain and wind are also the main environmental factors impacting pollen’s retention on leaf surfaces. For example, a study on corn pollen retention on milkweed leaves showed that a single rain event can remove 54–86% of deposited grains.^181^ Third, when wetted, pollen releases water-soluble compounds including, among others, sugars (e.g., fructose, used also as a chemical tracer for pollen^182,183^), polyunsaturated fatty acids, and proteins.^178,183−186^ Some of these substances have allergenic properties,^178^ whereas others can nucleate ice crystals.^187^ In the atmosphere, pollen can also release subparticles (0.5–3.0 μm in size)^182^ in a process that is triggered by water and/or high relative humidity and is common during thunderstorms.^182,183,185^ These subparticles are pre-existing bodies that can be found on the external surface (named “Ubisch bodies”)^176^ and/or inside (e.g., starch granules)^186,188^ intact pollen grains, depending on the species. Atmospheric pollutants can also interact with pollen in a variety of ways (reviewed by Sénéchal et al.^185^). For example, in urban areas, PM accumulates on pollen surfaces,^178^ whereas pollen-derived material has been observed on soot (e.g., Namork et al.^189^). Reactivity of pollen grains with air pollutants has also been investigated and is briefly summarized in section 4.4.2.

#### 4.1.3 Soil Particles

Soil particles have been detected on leaves in pastures and crop fields (reviewed by Smith and Jones^163^ and Collins et al.^190^) and sporadically on woody plants (e.g., Freer-Smith et al.^175^). The mechanisms responsible for the displacement of soil particles include wind erosion, mechanical disturbances, and animal grazing^163,190^—in addition to rain-induced dispersal (section 4.3). Overall, soil type has minimal impact on the quantity of particles found on leaves, whereas leaf morphology and distance from the ground play a more significant role.^163,166,190^ Mass loads for various crops and herbaceous plants range from 1.1 to 260 mg of soil per gram of leaf (reviewed by Smith and Jones^163^). These values are expected to vary considerably in the presence of removal agents such as wind and rain,^163^ and as a function of canopy height.^191^ As for generic PM (section 4.1.1), soil particles are more or less strongly bound to the surface and some of them (<5 mg per gram of leaf) may not be easily removed through wash off.^192−194^ Sheppard et al.^193^ suggested these strongly adhering particles to be aluminum silicate (clay) of comparable size of surface roughness features.

The occurrence and amount of soil particles on leaves can be estimated by comparing Al, Fe, Si, Ti, and rare earth metal content of full plant ash to that of the underlying soil.^193−195^ Of all elements, titanium and rare earth metals have less interferences due to their low concentration in plant tissues.^194,195^ This analysis is possible due to the prevalence of inorganic constituents in soil—as organic matter comprises only 1 to 5% of top-soil mass.^196^

#### 4.1.4 Semivolatile Compounds

In addition to particles, individual compounds can partition from the atmosphere onto dry or wet leaves. While many environmental science communities define this entire suite of chemicals as “semi-volatile”, atmospheric aerosol chemists further divide this category into subclasses depending on broad ranges of saturation vapor pressure (see, for instance, Donahue et al.^197,198^). Here, we adopt the World Health Organization’s convention and use the term semivolatile organic compounds (SVOCs) to encompass all chemicals with boiling points between 240/260 °C and 350/400 °C at standard atmospheric pressure.^199,200^ Many persistent organic pollutants and pesticides,^199^ and several sesquiterpenes, diterpenes, terpenoids, and other plant metabolites belong to this category.^201^ In principle, *volatile* organic compounds (i.e., chemicals with boiling point ≤240/260 °C; e.g., benzene, toluene, etc.) can also adsorb onto dry leaf cuticles. However, under outdoor conditions, they easily partition back to the gas-phase,^202,203^ making their relevance for leaf surface processes debatable–though if water-soluble, volatile compounds can interact with surface wetness and participate in multiphase processes (see section 5.4).

##### 4.1.4.1 Variables Impacting Dry Deposition

In general, SVOCs adsorption onto leaves depends on their lipophilicity and/or water solubility (the predominant factor depends on the SVOC’s molecular structure), variations in epicuticular wax chemistry, environmental conditions, and potential reactivity.^5,204^ Modeling studies indicated that dry deposition of POPs and semivolatile pesticides onto leaves is broadly controlled by their octanol-air partition coefficients (*K*_OA_).^28,31,190,205,206^ Variations in cuticular chemistry introduce an additional layer of complexity that led to the definition of plant-air partition coefficients (*K*_plant-air_). Empirical equations exist to calculate *K*_plant-air_ from the corresponding *K*_OA_ value (summarized by Taylor et al.^31^). For a given compound, *K*_plant-air_ can vary orders of magnitude depending on the plant species.^31,207^ For SVOCs with polar functional groups, the partitioning equilibrium is additionally impacted by the presence of leaf wetness, thus ambient relative humidity.^31,206^ This same trend has been observed for hydrophilic SVOCs formed from the atmospheric oxidation of biogenic and anthropogenic gases.^5,198,208^

The fraction of SVOCs deposited onto leaves changes dynamically in response to environmental variables. Under outdoor conditions, SVOCs adsorption occurs preferentially during cold nights, while warmer temperatures and sunlight favor re-emission.^206^ Joensuu et al.^209^ suggested that the adsorption-emission cycle may be particularly relevant for semivolatile chemicals that are poorly reactive toward atmospheric oxidants (e.g., sesquiterpene alcohols), as their atmospheric lifetime is longer. Besides diurnal variations, local climate (thus, season and latitude) can define the importance of vegetation as SVOCs sink,^207,210^ while changes in relative humidity are important for polar compounds. Ambient SVOC concentrations, which are impacted by meteorology and proximity to emission sources, can further affect the partitioning equilibrium.^206^ This fact is well exemplified by the observations that volatile compounds (e.g., benzene and toluene) have been detected on plant leaves primarily in indoor environments, where their gas-phase concentrations remains high due to limited air exchange.^202,203,211,212^

In addition to direct gas-phase partitioning, SVOCs can also reach plant surfaces *indirectly* through PM deposition. This process involves three steps: (1) SVOCs adsorb onto particles in the gas-phase; (2) particles deposit on leaves; and (3) SVOCs migrate from the particle to the leaf surface.^163,190^ The relevance of this additional mechanism depends on a complex interplay of factors including the SVOC’s vapor pressure and the particle surface chemistry, as well as ambient temperature, relative humidity, and the aerosol’s residence time on the leaf.^163,213^ According to Cousins and Mackay,^213^ direct SVOC adsorption onto leaves is predominant for organic compounds with 6 < log(*K*_OA_) ≤ 9, whereas particle-bound transfer is the main delivery route when log(*K*_OA_) > 9. A few empirical studies focusing on PAHs and other persistent organic pollutants supported the existence of this indirect delivery pathway.^169,190,214,215^

##### 4.1.4.2 SVOCs Detected on Leaf Surfaces

Both anthropogenic and biogenic SVOCs have been detected on leaf surfaces. The former group comprises POPs and pesticides. Several studies used plant leaves as passive samplers for persistent organic pollutants including PAHs, polychlorinated biphenyls and other chlorinated hydrocarbons, and dioxins (summarized by Wetzel and Doucette^203^ and others^207,210,216^). Reported surface concentrations range from <1 ng to up to tens of μg per gram of leaf dry weight,^207,210,216^ with PAHs being overall the most abundant contributors (see also Figure S2).^210^ Although generally deposited via wet routes (section 4.1.2), many pesticides are semivolatile^31^ and undergo evaporation/deposition cycles. (This observation is also supported by the fact that volatilization is a major pesticide loss mechanism in the environment.^28,206^) Indeed, pesticides have been found on nontarget plants growing nearby agricultural fields (e.g., Essumang et al.^217^). Considerable levels of pesticides have also been measured in dust and indoor surfaces in households nearby agricultural areas (reviewed by Dereumeaux et al.^218^).

Biogenic SVOCs from *exogenous* sources have also been found on leaves. For instance, some authors observed daily adsorption and re-emission cycles of ledene, ledol, palustrol, and aromadendrene (two sesquiterpene alcohols and two sesquiterpenes, respectively) from birch (*Betula sp*.) leaves.^201,219^ Birch does not produce these compounds–rather, they were emitted by an understory shrub (*Rhododendrum tomentosum*) and picked up by overlying birch leaves. Likewise, Joensuu et al.^209^ posited that sesquiterpenes reach *Pinus sylvestris* needles via dry deposition after being released from a surrounding plant or a different plant organ. This conclusion was based on the observation that sesquiterpenes’ content and speciation differ strikingly in wax extracts and needle emissions from the same plant. Analogous processes were also observed in laboratory settings on other tree species.^220−222^

### 4.2 Wet Deposition

#### 4.2.1 Hydrometeors

Hydrometeors, which include rain, snow, mist, and fog, scavenge compounds and particles present in atmosphere and can deliver them to leaf surfaces (Figure 5). At the same time, hydrometeors can remove exogenous and endogenous compounds and particles through leaf wash-off (section 4.1.1) and aerosolization (section 4.3), induce the leaching of metabolites and nutrients (section 5.3), and modify the chemistry of leaf wetness (section 5.4). The relative importance of these opposite processes depends on the interplay of numerous factors including hydrometeor type and total carbon concentration, intensity of the meteorological event, leaf surface features and location within the canopy, and physicochemical properties of the deposited compound or particle.

##### 4.2.1.1 Behavior of Rain on Leaf Surfaces

In general, only drops and water films that persist on leaves until evaporation contribute to the pool of leaf-adsorbed chemicals (Figure 5).^223,224^ This fact has been well established for particulate and dissolved radionuclides delivered onto pastures and crops by contaminated rain (reviewed by Pröhl^225^ and Anspaugh^226^); with some limitations (discussed at the end of this section), we expect the same principles to be valid also for nonradioactive species and other vegetation types.

The fraction of incoming radionuclides intercepted by and retained on leaf surfaces is called the interception factor (*f*). This parameter is obtained as the radioactivity measured on standing vegetation divided by that of incoming precipitation.^225,227^ Empirical values for *f* span from 0.006 to >0.95,^224,225,227,228^ reflecting the complex dependence of this variable on features of the plant, incoming precipitation, and wet-deposited material.^223,225,227^ For >1 mm of rain, *f* is typically between 0.01 and 0.3–0.5.^227,228^ The interception factor is conceptually related to the fraction of precipitation lost to evaporation at the top of the canopy (*E*_i_/*P*; see, e.g., Lian et al.^229^). Recent modeling^229^ and meta-analysis^230^ studies showed that *E*_i_/*P* averages 0.20–0.25 globally, with large variations (i.e., ≈0 to 1) depending on biome, leaf type, climate, and storm conditions. The similarities between values and drivers for *f* and *E*_i_/*P* strengthen our hypothesis that the knowledge for wet deposition of radioactive elements can be translated to nonradioactive chemicals and particles.

Development stage and water storage capacity are two important plant features influencing the magnitude of the interception factor. In general, *f* increases during plant development because more surface area becomes available for intercepting rain.^223,225^ Plant development can be estimated from the standing plant biomass (i.e., the dry mass of plant per m^2^ of soil) or the leaf area index (i.e., the single-sided total leaf area of the plant per area of soil).^225^ When normalized by the standing plant biomass (0.05–0.35 kg m^–2^), *f* for pasture plants and crops is 0.06–11 m^2^ kg^–1^ (summarized by Gonze and Sy^223^). The canopy storage capacity (*S*) is another key factor impacting the value of *f*. This parameter describes the amount of water that can be held on leaves before rolling off–this happens when the accumulated water mass becomes too heavy to outweigh the leaf’s water surface tension.^223,231^*S* depends on leaf area, orientation, and surface properties, and ranges from 0.1 to 4.3 mm depending on plant species (see also section 5.1).^231,232^ The canopy water storage capacity is also impacted by preexisting surface wetness—*S* is lower for wet than dry leaves—and decreases in the presence of wind and other mechanical disturbances.^225,232^ The important role of the leaf water storage capacity justifies empirical observations that *f* decreases with amount of rainfall.^224,227,228,233^ Leaf area index, canopy storage capacity, and rainfall amount have also been identified as drivers of *E*_i_/*P*, with rainfall characteristic being more influential than vegetation attributes at the global scale.^229^

Last, the chemistry of wet-deposited material strongly influences its fate. In general, radionuclides in particulate forms (>3 to 100 μm) and cations (e.g., ^7^Be^2+^) are retained on leaf surfaces, whereas dissolved anions (e.g., ^131^I^–^ or ^34^SO_4_^2–^) are washed off as efficiently as water.^224,225,227,233^ For example, in the same simulated rain event, Hoffman et al.^224^ measured an average interception fraction of 0.08 for ^131^I^–^, 0.28 for ^7^Be^2+^, and 0.30–0.37 for radionuclides embedded in polystyrene microspheres (3–25 μm in diameter). This trend was found to be independent of plant type.^233^ The different behavior of dissolved radionuclides has been justified in terms of electrical properties—being negatively charged, leaf surfaces attract cations and repel anions.^223,225,227^ Like cations, particles are understood to settle and adsorb on the surface, which partially prevents their subsequent wash off when the water storage capacity is reached. As observed for dry deposition of particulate matter (section 4.1.1), smaller particles adsorb more efficiently than larger ones.^224,225^ In the worst-case scenario, dissolved organic molecules present in rainfall may behave as negatively charged radionuclides–thus, their interception fraction can be estimated directly from *E*_i_/*P* values. Individual molecules with hydrophobic domains or positive charges may interact with the leaf surface, resulting in *f* > *E*_i_/*P*, whereas volatile compounds (e.g., small organic acids) may volatilize during water evaporation, leading to *f* < *E*_i_/*P*.

Despite the clear evidence showing that rain can deliver chemicals onto leaf surfaces, the radionuclide literature has a few biases worth highlighting. First, interception fractions are calculated from the bulk radioactivity of whole leaves and do not distinguish between material *adsorbed onto* the cuticle and taken up by the plant. While particles >1 μm do not enter leaf tissues,^234^ dissolved cations can (see sections 5.2–5.3)—thus, the amount of positively charged chemicals on leaf surfaces may be overestimated if deduced from the interception factor. Second, *f* has primarily been measured for grasses and crops,^223,225^ and only sparsely for saplings of woody plants (e.g., Hoffman et al.^233^). When considering fully grown trees, these values are representative only of leaves at the top or on the outside of the canopy. *E*_i_/*P* values are also referred to top of the canopy conditions.^229^ Hoffman et al.^233^ posits that *f* should increases inside tree crowns because drops have higher chances to be intercepted; we are not aware of studies confirming or disproving this hypothesis. We further note that rain chemistry will change considerably as drops move through the canopy (section 5.3), complicating the assessment of rain’s contribution to the leaf’s chemical landscape.

##### 4.2.1.2 Behavior of Other Hydrometeors on Leaf Surfaces

In the presence of fog, mist or low clouds, tiny water droplets suspended in the atmosphere condense onto leaf surfaces, forming water films that are generally less than 0.5 mm thick.^235^ Compared to rain drops, which have diameters from 0.1 mm (splash throughfall)^236^ to up to 5.5 mm^236,237^ and can easily roll off, water films are more likely to remain on the leaf onto which they first formed–although, if the cumulative amount of water exceeds the water storage capacity, they may grow into droplets and fall to the ground with their load of chemicals.^238,239^ For this reason, contribution of surface chemicals are expected to be rather homogeneous across the canopy. Similar to rain,^229^ evaporation of water films takes a few hours and is favored by wind and sunlight (e.g., Wentworth et al.^240^).

On the other hand, we expect snow and dew to contribute negligibly to the pool of leaf surface chemicals. Although canopies retain up to ten times more snow than rain^232,241^ for up to several weeks,^241^ snow is solid and porous, with only a single layer of flakes in contact with the leaf at any given time. When snow melts, a layer of liquid water forms between the overlying flakes and the leaf surface—however, it is unlikely for chemicals in this melted layer to stick on the leaf, as its presence decreases snow’s adherence and facilitates its sliding from the branch.^242^ A rare study comparing snow and rain supports our hypothesis by showing almost no change in total phenolics (as compared to its control) in snow collected below a spruce, but a considerable increase of these compounds in rain throughfall from the same tree.^243^ Despite the apparent similarity with fog, dew forms when water *vapor* (thus, individual water molecules) condenses on a cold surface.^244^ Although the resulting film can rapidly pick up water-soluble gases and further engage in multiphase processes,^240,245^ dew *per se* is pure water and does not contribute surface chemicals.

##### 4.2.1.3 Chemicals in Hydrometeors

The concentration of chemical compounds and particles in hydrometeors is a key factor defining the relevance of wet deposition. In the 1980s, scientists studying the impact of acid rain on plants concluded that mist, fog, and clouds are more relevant contributors to ecosystem acid deposition than rain because of their higher solute concentrations.^246^ Even though this deduction was based on inorganic species, present-day organic carbon concentrations are also lower in rainwater (typically 0.02–13 mg_C_ L^–1^, averaging ≈2 mg_C_ L^–1^; Figure S3)^247^ as compared to cloudwater and fog (typically 0.10–41 mg_C_ L^–1^, averaging ≈ 15 mg_C_ L^–1^; Figure S4),^248^ hinting that this trend may be valid in general.

Specific organic compounds have also been identified in hydrometeors. Formic and acetic acid have been ubiquitously detected in rain, fog, and cloudwater in concentrations that are relevant for the total organic carbon budget.^248−250^ Less abundant low-molecular-weight compounds include organic acids, such as oxalic, lactic, malonic, and succinic acids; carbonyls like formaldehyde, glyoxal, and methylglyoxal; amino acids; and levoglucosan.^248−250^ Rain also contains POPs like perfluoroalkyl substances (PFAS), organophosphate esters, and PAHs in tens to hundreds of ng L^–1^ (cumulative concentrations for each class, summarized by Casas et al.^251^ and Guo et al.^252^). Likewise, PAHs, nitrosamines, nitrophenols, pesticides, and other anthropogenic chemicals have been detected in fog and cloudwater (reviewed by Herckes et al.^248^), with individual compounds in concentrations ranging from hundreds of ng L^–1^ to tens of mg L^–1^ (e.g., Khoury et al.^253^). Particulate matter is also found in hydrometeors, although its contribution to the total carbon budget is minimal—it ranges from negligible to 35% depending on location, time of the year, and occurrence of specific events.^247,252,254^ These insoluble particles include black carbon,^255,256^ primary biogenic particles (e.g., bacteria, pollen, fungal spores),^257^ soot (including elemental carbon),^257^ soil minerals particles,^257^ and microplastics.^258−260^

#### 4.2.2 Sprays for Agricultural Use

Wet deposition may be particularly relevant in agricultural settings because pesticides are often applied as aqueous sprays. Pesticide formulations contain the active ingredient and one or more surfactants in relatively high amounts (up to 10% by weight).^261^ Surfactants enhance leaf wettability, and thus both droplet retention on leaves and cuticular permeability—two features that increase the plant’s uptake of the active ingredient.^261−263^ Depending on their molecular structures, surfactants can also influence pesticide reactivity (thus, persistence) on leaf surfaces (see also section 4.4.).^264^ The most common surfactants in pesticide formulations include nonionic (e.g., polymerized glycol ether) and anionic compounds (e.g., linear alkylbenzenesulfonates),^261^ with new and more environmentally friendly alternatives being constantly developed.^261,265,266^

Active ingredients persist on crop leaves for a variable amount of time depending on the specific combination of the pesticide’s physicochemical features and reactivity, and on the leaf’s surface chemistry and morphology. For example, Das et al.^267^ detected chlorpyrifos, an organophosphate pesticide, in concentrations of 21.6 μg g^–1^ immediately following spray application on Purple tansy leaves, dropping below 5 μg g^–1^ already the following day. For this pesticide, literature values for DT_50_, the time required to halve the initial active ingredient concentration by 50%, range from 0.4 to 166 h depending on the leaf type (summarized in Das et al.^267^), underscoring the importance of leaf surface properties in controlling pesticides’ uptake and environmental fate (see also sections 4.1.4.1 and 4.1.4.2).

### 4.3 Deposition Facilitated by Hydrometeors

In addition to acting as a wet deposition or leaf cleansing agent, rain falling on environmental surfaces can generate or facilitate the release of particles that are then deposited onto nearby vegetation via wet and dry deposition. This mechanism has been reported for soil particles,^163,190,268,269^ soil bacteria,^270^ plant pathogens,^271,272^ pollen-derived aerosols (see also section 4.1.2),^185^ and spores of some fungal species,^273^ and can be considered a “mixed type” of deposition pathway primarily with local relevance.^268^

The mechanistic details of how hydrometeors affect deposition vary across particle type, leaf type, and environmental circumstance. For example, submicron aerosol containing soil microbes and soil organic matter form through a “bubble bursting” mechanism triggered by the entrapment of air films between fallen droplets and porous surfaces.^268,270^ This process requires unique conditions to take place—namely, light or medium intensity rain falling onto unwetted sandy-clay or clay soils^268^—and is expected to be relevant only in specific ecosystems (e.g., agricultural areas and grasslands).^268,269^ Rain splash is another mechanism that delivers larger soil-derived particles to leaves growing up to 1.5 m from the ground.^163,274^ Fungal spores are dispersed differently. Kim et al.^273^ showed that rain droplets falling onto maize leaves infected with the rust fungus *Puccinia triticina* trigger spore release via wet and dry mechanisms. The wet pathway is a splash-release dispersal that involves the generation of daughter drops after raindrop impact onto an infected area. These smaller spore-containing drops are expected to fall only onto nearby or underlying leaves due to their large size.^272^ Dry spores are also ejected when leaves vibrate following a drop’s impact or as a result of the spreading motion of a fallen drop. The impact further generates an air vortex that drives dry spores away from the surface allowing longer-range dispersal. Different fungal species adopt different spore dispersal mechanism depending on their survival strategy.^272^

### 4.4 Reactivity

#### 4.4.1 Observed Leaf-Surface Reactivity of Anthropogenic SVOCs

Although there is compelling evidence for the *presence* of exogenous substances onto plant leaves, information on their multiphase reactivity is scarce and biased toward anthropogenic compounds. To the best of our knowledge, leaf-surface photodegradation of pesticides is the only process that has been investigated in detail (reviewed by Sleiman et al.^275^ and others^29,264^)—although primarily in laboratory settings using model surfaces or reconstructed cuticles.^29,275,276^ Some work has also been performed on the photodegradation of PAHs and their oxidation products on and within leaf cuticles,^277−279^ while other studies investigated HNO_3_/nitrate photolysis on leaves of various plants.^280,281^ On the contrary, the reactivity of anthropogenic SVOCs adsorbed on leaves with other gas-phase oxidants has received considerably less attention.^264^ An early investigation reported the oxidation of parathion (a pesticide) adsorbed onto lemon tree leaves in the presence of ozone and “foliar dust” (soil organic matter particles).^282^ In more recent years, a handful of studies further described the multiphase oxidation of pesticides deposited onto vegetable leaves by gas-phase hydroxyl radicals and ozone.^283−286^ The interest in this topic has been driven primarily by the potential of ozone (both in the gas-phase and dissolved in water) as a “green” strategy to eliminate pesticide residues from fruits and vegetables (reviewed by Pandiselvam et al.^287^).

**Figure 6:**
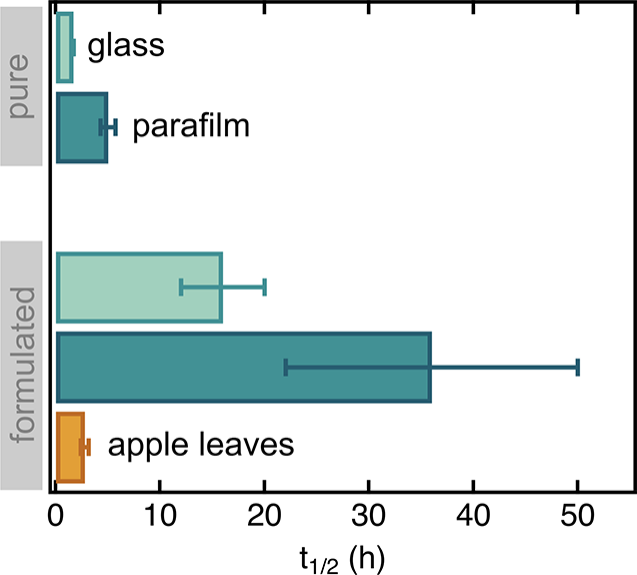
Photolysis half-lives (*t*_1/2_) of the plant protection product acibenzolar-S-methyl deposited on model (green) and natural (orange) substrates, both as a pure ingredient (top) or as component of a commercial formulation (bottom). Higher half-lives mean slower photodegradation. Data replotted from Sleiman et al.^288^

Regardless of the specific compound, the photochemical reactivity of SVOCs on leaves follows similar principles. Overall, photodegradation obeys pseudo-first-order kinetics, with reaction rate constants that change considerably (but not predictably) based on chemistry, surface coverage, and micromorphology of the reaction substrate, as well as co-occurrence of other substances (e.g., Figure 6).^29,264,277−280^ Formulation ingredients (e.g., surfactants) and co-occurring volatile and semivolatile metabolites can further influence surface photolysis by acting as photosensitizers or quenchers of reactive species, stabilizing radical intermediates, screening light, or a combination of these processes.^29,121,264,289^ By modifying the leaf’s wettability, surfactants can also influence shape, density, and crystallinity of the active ingredient’s residue, with impacts on its photochemical stability.^29,288^ Variation in epicuticular wax chemistry, thickness, and morphology impact photodegradation in a similar manner.^275^ Wax components may act as photosensitizers or quenchers of reactive species and actively participate in reactions to form “bond residues”, whereas the presence of specific microstructures modifies light transmission and water spreading.^29,264,275^ SVOC solubility in epicuticular waxes can also impact half-lives, as compounds buried within the cuticle are less susceptible to photodegradation than less lipophilic molecules sitting on its surface.^278^ Despite this comprehensive set of empirical observations, the mechanistic understanding of leaf surface photolysis is still largely speculative.

Environmental parameters additionally impact surface photolysis. Temperature, wind speed, and light have received the most attention so far, whereas ambient relative humidity has been largely overlooked. Both temperature (during irradiation) and wind speed increase the relative importance of volatilization vs photodegradation,^28,284^ with their absolute effect depending on the pesticide’s physicochemical properties. Xi et al.^290^ also evaluated the impact of the plant’s growing temperature on photodegradation rates, observing a reduced loss of leaf-adsorbed pyrethroids as a function of irradiation time for spinach plants grown at 21 vs 15 °C. The change was attributed to variations in cuticular wax chemistry as a function of growing temperature—a phenomenon that has also been described by other authors.^59,60^

Light intensity and spectral composition are two key variables driving photochemical processes, but lab-based investigations rarely represent realistic field conditions. Most studies use horizontal surfaces kept under full irradiation, while, within the canopy, most leaves are shaded and/or not necessarily perpendicular to the incoming radiation.^291^ Furthermore, overlying leaves will selectively absorb certain wavelengths, resulting in spectral variations within the canopy as compared to the top-canopy irradiance.^292,293^ For plants grown indoor, the fraction of available UV–B radiation will also be considerably reduced due to light absorption by glass windows.^264,294^

Last, there have been limited assessments of how ambient relative humidity affects leaf surface photodegradation. When reported, RH typically refers to the plants’ growing conditions, not the irradiation experiment (e.g., Xi et al.^290^). In some cases, pesticides are deposited onto surfaces as aqueous solutions, but water is either allowed to evaporate before irradiation (e.g., ter Halle et al.^295^) or it is used as the reaction solvent (e.g., Anderson et al.^276^). The known impact of relative humidity in other multiphase systems (e.g., for the hydroxyl radical oxidation and ozonation of pesticides adsorbed on silica particles)^296,297^ and the fact that photodegradation kinetics are different in bulk aqueous solutions than on leaf surfaces^276,295^ underscores the need for a deeper assessment of this variable.

#### 4.4.2 Expected Leaf-Surface Reactivity of SVOCs and Particles

Empirical evidence of leaf surface reactivity for chemicals other than pesticides, PAHs, and HNO_3_/nitrate is lacking. However, as Dibley et al.^129^ pointed out, multiphase reactions of leaf-adsorbed compounds may be analogous to those occurring on outdoor and indoor organic surfaces, and on inert substrates. In analogy to anthropogenic SVOCs on leaves, studies on the reactivity of urban grime have focused on photochemical processes, but with an emphasis on nitrous acid (HONO; summarized by Kroptavich et al.^25^) and, recently, sulfur compounds.^95^ Surface ozonation of adsorbed PAHs has also been described, but only on model organic films.^298^ In recent years, an increasing number of investigations characterized the multiphase reactivity of individual compounds and organic films ubiquitously present on indoor surfaces.^299−301^ Most studies focus on ozone and have clearly demonstrated that skin lipids (e.g., squalene) and terpenoids from consumer products undergo multiphase ozonation.^300^ Other well-established multiphase reactions occurring indoor include the formation of HONO by dissolution of gas-phase NO_2_ into adsorbed water or via photochemical processes; acid–base partitioning of ammonia, amines, and organic acids; hydrolysis; and reactions induced by chlorine-based oxidants (reviewed by Ault et al.^301^ and others^299,300^). While some of these processes will only be relevant in human-occupied areas, others may also occur on leaf surfaces (e.g., acid–base equilibria^4^). The multiphase oxidation of pesticides has also been investigated on inert substrates. For example, solid films of neonicotinoids (deposited on silica) have been shown to react with gas-phase ozone,^302^ hydroxyl radicals,^303^ and sunlight,^304^ yielding both volatile (e.g., HONO)^302^ and nonvolatile products.

In addition to individual compounds, there is strong evidence for the multiphase reactivity of particulate matter with gas-phase oxidants and light, both when suspended in air and when deposited onto surfaces (reviewed, e.g., by George et al.^305^). Pollen also interacts with gas-phase O_3_ and NO_*x*_, causing changes in elemental composition and increased tendency to crack and release submicron particles (reviewed by Sénéchal et al.^185^). Atmospheric processing of pollen is of high interest given the established link between air quality and severity of seasonal allergies. Exposure to polluted air has also been linked to nitration of tyrosine residues of specific pollen allergens (e.g., in birch pollen^306^). Thus, it is highly probable that pollen undergoes multiphase chemistry also when deposited on leaf surfaces.

## 5 Leaf Wetness

Water plays a crucial role in impacting the chemical landscape of leaf surfaces. In previous sections, we showed that hydrometeors influence the flux of exogenous chemicals and particles by acting both as a source of material (sections 4.2.1) and as a cleansing agent (section 4.1.1). High relative humidity can also induce plants to release guttation drops from leaf tips (section 3.2). Water availability further impacts fitness and metabolism of phyllosphere microorganisms (section 3.4)—thus, the compounds they excrete and the chemical transformations of those already present—as well as rates and mechanisms of leaf surface reactions (section 4.4).

In addition to the former processes, water allows the establishment of mass transfer pathways across the cuticle and enables surface aqueous chemistry, further contributing to the complexity and dynamicity of the leaf surface’s chemical landscape. In this section, we briefly review the various forms of leaf wetness and their environmental occurrence (section 5.1) and describe mechanisms of cuticular mass transfer in the presence and absence of water (section 5.2). We then illustrate available evidence supporting the bidirectional exchange of water, nutrients, and metabolites through wet cuticles (section 5.3) and of atmospherically relevant species through surface wetness (section 5.4).

### 5.1 Types of Leaf Wetness and Their Occurrence

Leaf wetness can be macroscopic or microscopic. Macroscopic wetness refers to forms of water that are visible to the naked eye, such as rain drops (0.1–5.5 mm in diameter)^233,236,237^ and water films formed in the presence of dew, fog, haze, mist, clouds, or during prolonged rain events (≤0.5 mm in thickness).^235^ Macroscopic wetting occurs on average >100 days per year across all biomes (ranging from 29 days year^–1^ for deserts to 174 days year^–1^ for tropical and subtropical forests), with leaves remaining wet on average (8.7 ± 2.5) hours per day.^307^ Overall, broadleaves and conifers store up to 0.1–2.0 and 0.1–4.3 mm of water, respectively, on their canopies, with variations depending on leaf structure and meteorological variables (reviewed by Klamerus-Iwan et al.;^232^ note that the canopy storage capacity is expressed as “mm” to indicate liters of water per m^2^ of land). Macroscopic wetness responds dynamically to changes in meteorological conditions. For example, night dew evaporates during the day when temperature increases and sunlight reaches leaf surfaces; a decrease in ambient relative humidity and occurrence of winds further accelerates evaporation.^308^

**Figure 7:**
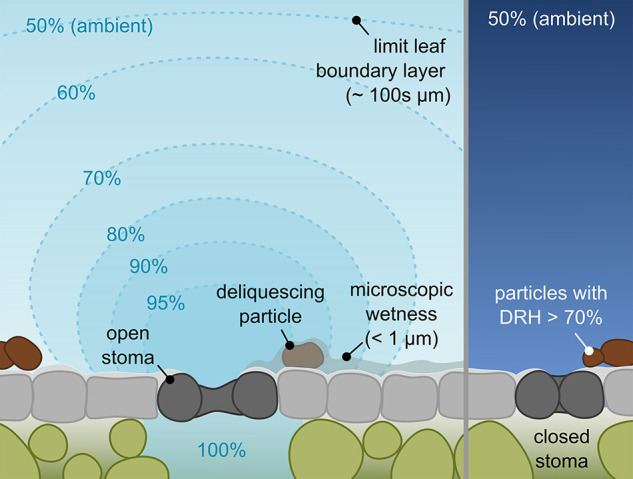
Schematic illustrating the formation of microscopic wetness from deliquescing particles. Ambient conditions have minimal influence when stomata are open (e.g., during the day; left); the situation reverses when stomata close (e.g., during the night; right). The schematic assumes that all particles have a deliquescence relative humidity (DRH) > 70%. Percentages indicate relative humidity values. Figure inspired by Burkhardt and Eiden.^309^

Conversely, microscopic wetness denotes water layers <1 μm, which form from stomatal transpiration and, potentially, condensation of atmospheric water vapor. According to Burkhardt and Hunsche,^235^ microscopic wetness is permanently present on leaves, even when they look dry and ambient relative humidity (RH_amb_) is low. In analogy to the Earth’s atmosphere, leaves are surrounded by a boundary layer that extends micrometers to millimeters from their surface and is characterized by different temperature, relative humidity, and gas transport properties compared to the surrounding air.^39,140,166,235^ In particular, when stomata are open (typically but not uniquely during the day), the relative humidity in the leaf boundary layer (RH_leaf_) > RH_amb_ due to stomatal transpiration, often reaching values above 75% (Figure 7).^235^ The high RH_leaf_ allows hygroscopic salts present on leaves (which may originate from deposited aerosols or guttation) to become deliquescent, resulting in the formation of wet areas on the leaf surface, mostly localized around stomata.^235,310,311^ This mechanism is similar to water uptake by cloud condensation nuclei in the atmosphere.^166^ Cuticular water uptake (see section 5.2), adsorption of gas-phase water molecules, capillary condensation, and evaporation of drops in areas populated by bacterial aggregates have been proposed as additional processes that contribute generating microscopic wetness.^235,312^

When stomata are closed (e.g., during nighttime), ambient RH plays a more significant role in forming and maintaining microscopic leaf wetness (Figure 7).^235^ The factors driving its formation are the same as with open stomata; however, as most salts do not deliquesce <70%,^313^ other mechanisms may become predominant. For example, Hu et al.^314^ observed absorption of individual water molecules on clean mica (a hydrophilic surface) < 5% RH, a continuous monolayer between 20 and 40% RH (0.2 nm thick), and additional layers of mobile, liquid water from 40% to 100% RH (up to 2 nm thick). It is well-known that water absorption depends on surface properties, especially hydrophobicity (reviewed by Xiao et al.^315^). Leaves have a wide range of contact angles, ranging from ≤40° (superhydrophilic) to ≈180° (superhydrophobic),^316,317^ with temperate species showing values >60°.^318^ Thus, depending on plant species and ambient relative humidity, individual water molecules may preferentially adsorb onto bacterial aggregates and deposited aerosol particles (without inducing deliquescence) rather than the cuticle itself. In all cases, the wet layer that may form on leaves when stomata are closed is orders of magnitude thinner (i.e., a few nm) than the one generated by stomatal transpiration.

### 5.2 Mechanisms of Cuticle Permeability in the Presence and Absence of Leaf Wetness

*While cuticles are considered a protective layer, they still allow chemicals to move between the mesophyll and the leaf surface. Transport through the cuticle is a passive, bidirectional process that is driven by gradients in concentration and, for charged molecules, electric potential*.^262^*Compounds of different hydrophilicity move through the cuticle via different pathways. The behavior of lipophilic compounds can be effectively predicted with the “solution-diffusion model”, according to which the penetration rate of a given molecule is proportional to its partition coefficient between the external solution and the cuticle (i.e., its solubility) and its diffusion coefficient through the cuticle (i.e., its mobility)*.^262,319^*A compound’s mobility is negatively correlated with molecular weight, with larger molecules being less mobile than small ones, and is strongly enhanced by increasing temperature (see Riederer and Friedmann*^319^*for more details)*.

**Figure 8:**
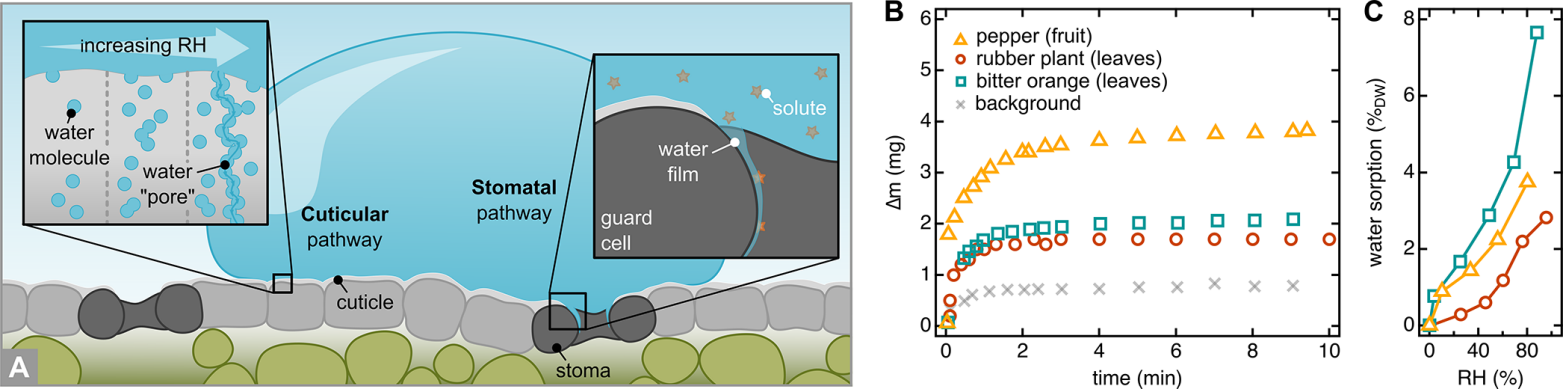
(A) Schematic illustrations of cuticular (right) and stomatal (left) pathways for the uptake of water and solutes (redrawn from Fernández et al.^76^). (B) Water absorption kinetics in isolated cuticles of pepper (*Capsicum annuum*; fruit cuticle), rubber plant (*Ficus elastica*; leaf cuticle), and bitter orange (*Citrus aurantium*; leaf cuticle) at 48–50% RH. The *y*-axis is the mass increase measured via magnetic suspension balance. The background trace is obtained with an empty measurement chamber and is shown for reference (experimental traces are *not* corrected for background). (C) Relative increase in the cuticle’s mass (in percent of dry weight, %_DW_) as a function of RH for the plant species in panel B. Data in B and C are replotted from Chamel et al.^324^

*The situation is different for hydrophilic molecules, whose exchange between the leaf interior and the surrounding environment is mediated by leaf wetness. Two mechanisms have been proposed but are based on indirect empirical evidence rather than direct observations: (i) the cuticular pathway and (ii) the stomatal pathway (reviewed by Fernández et al*.^76,262,308^Figure 8).

The cuticular pathway involves the movement of solutes through “water pores”, dynamic channels that form upon absorption of water molecules by the hydrophilic domains of the cuticle (i.e., polysaccharides and unesterified hydroxyl, carboxylic, and ester groups; Figure 8A, left).^308,320^ (Note that here “water pore” is *not* a synonym for “hydathode”—though these terms are sometimes used interchangeably.^52,55,56^) The major evidence for the existence of water pores is the ability of cuticles to swell in contact with water, with reported increase in mass of 1–20% for isolated cuticles depending on the plant species and ambient relative humidity (Figure 8B–C).^308,321,322^ Like hydrophobic compounds, the molecular weight–thus hydrodynamic size–defines which solutes can traverse the cuticle. Estimated cuticular water pore diameters range from 0.3 to 4.8 nm, dimensions that allow sugars and chelated micronutrients to pass through.^76,262^ Molecular charge is another important feature that controls the extent of cuticular transfer. As the bottom of the cuticle is more negatively charged than its surface, cations, and anions are taken up or released at different rates depending on the solution’s ionic strength (see Fernández and Eichert^262^ for details). Several other parameters influence the cuticular pathway, namely, ambient RH and temperature, pH, and ion composition of the applied solution, leaf age, and plant species.^262^ Furthermore, cuticular water pores are often unevenly distributed across the leaf surface, with the specific location depending on the plant species.^35,62,323^ For instance, the base of trichomes are preferential sites for fructose permeability in isolated cuticles of poplar leaves, whereas other species have high abundance of cuticular water pores close to stomata guard cells.^323^

The stomatal pathway involves the diffusion of hydrophilic solutes through water films covering the surface of stomata guard cells (Figure 8A, right).^76^ The existence of this pathway is based on the observation that the uptake of aqueous nutrient solutions deposited onto leaves is positively correlated with presence, density, and degree of aperture of leaf stomata even though direct water infiltration is prevented by their architecture.^76,308^ (In general, direct infiltration of aqueous solutions through stomata requires the application of an external pressure or the presence of surfactants.^76,325,326^) Stomata need to be “active” for this pathway to be operative: activation has been observed in the presence of hydroscopic particles, bacteria, or fungal hyphae,^76,234^ which are also involved in the formation of microscopic wetness (section 5.1). Overall, the stomatal pathway is deemed more efficient than the cuticular pathway in mediating the transport of hydrophilic solutes across the cuticle, also because of its higher equivalent pore radius (3.5 to >100 nm).^234,327^ This mechanism also contributes to the transfer of nanoparticles (≪1 μm) to the leaf’s interior.^234^

### 5.3 Bidirectional Exchange of Compounds through the Cuticle

After the establishments of pathways for mass transport, compounds can move across the cuticle in two directions, with the prevalence of one over the other being controlled by their concentration and ionic gradients.^262^ Overall, leaching of nutrients and metabolites following rain or other natural wetting phenomena is ubiquitous,^238^ whereas the uptake of nutrients requires highly concentrated solutions (10^–3^ to >1 mol L^–1^).^76,262^ Thus, under natural setting, release of water-soluble metabolites likely outweighs uptake due to the overall diluted character of hydrometeors (section 4.2.1.3). The situation is different for hydrophobic compounds. Secondary metabolites can be present in high concentrations in glandular trichomes and other specialized epidermal cells, which creates a driving force for their excretion,^104^ while pollutants and pesticides are less concentrated inside the leaf as compared to their surface and are thus more prone to be taken up (e.g., Wang et al.^328^).

Whereas evidence for foliar uptake is limited, there is ample data hinting that leaf wetness induces the release of plant metabolites. Most evidence for this process was gathered between the 1950s and 1980s and has been summarized by Tukey in a series of reviews.^238,329,330^ Briefly, both organic and inorganic substances are leached. Organic metabolites include free sugars and sugar alcohols, pectic substances, amino acids, organic acids, growth-regulating chemicals, vitamins, alkaloids, and phenolic compounds, with carbohydrates being the most easily leached compound class. Inorganic species comprise, among others, K^+^, Ca^2+^, Mg^2+^, and Mn^2+^;^330^ K^+^ and Mn^2+^ have also be employed as tracers for leaf leaching (e.g., Burkhardt et al.^88,331^). Leaching rates vary across not only plant species, but also individual plants of the same species and even leaves of the same plant; ambient temperature, surface wetting properties, leaf age, and the chemistry of surface wetness further influence this process. An overarching finding is that leaves just need to be wet to leach–thus, dew, fog, and light, prolonged rain induce more efficient leaching than short and intense rain. This early literature identified guttation and excretion from glandular trichomes and nectaries as possible mechanisms underpinning this process, whereas leaching through stomata was deemed negligible.^330^ Remarkably, the observations presented by Tukey align well with those that led to the identification of cuticular and stomatal pathways in the foliar fertilization literature (section 5.2). However, to our knowledge, the hypothesis that “leached” chemicals reach leaf surfaces through cuticular water pores remains untested.

A second line of evidence for water-induced leaching is the observation that rain becomes enriched in organic chemicals as it travels through the canopy (reviewed by Van Stan and Stubbins^332^ and others^101,333^). Over the past 30 years, several studies reported significantly higher dissolved organic carbon concentrations in rain samples collected below trees, either as throughfall (5–57 mg_C_ L^–1^) or stemflow (7–332 mg_C_ L^–1^), compared to controls collected in an open canopy space (0.3–2 mg_C_ L^–1^).^332^ This organic carbon is commonly referred to as tree dissolved organic matter or tree-DOM and is a complex mixture of organics that encompasses aromatic (16–30%) and aliphatic (24–31%) compounds, carbohydrates (14–25%), and a small amount of black carbon.^174,334^ Some of these constituents absorb sunlight, making tree-DOM potentially susceptible to photodegradation.^332^ According to the current understanding, tree-DOM is produced *in situ* through the erosion of epicuticular waxes during rain,^335^ lignin degradation,^336^ and excretion of compounds by the plant, its epiphytes,^100^ and its phyllosphere microorganisms.^336^ To our knowledge, also this field does not consider leaching through cuticular water pores as a potential source of tree-DOM constituents—although the high bioavailability of this mixture^332^ fits with this view. Some inorganic ions (e.g., K^+^ and Mn^2+^) are also present in higher concentrations in throughfall compared to the incoming precipitation (see Ponette-Gonzáles et al.^101^ and refs therein). A few authors (e.g., Lequy et al.^337^) quantified particulate matter content (>0.45 μm) in throughfall, observing an enhancement as rain travels through the canopy–however, as we discussed in section 5.2, particles larger than 1 μm cannot cross the cuticle, implying that they originate from the wash-off of material deposited *onto* leaf surfaces.

### 5.4 Bidirectional Exchange of Atmospheric Gases through Leaf Wetness

In addition to establishing mass transfer pathways across the cuticle, surface wetness also mediates the leaf’s interaction with the atmosphere. Historically, this process has been investigated for water-soluble inorganic gases (i.e., NH_3_ and SO_2_) and ozone, but an increasing body of knowledge indicates that other organic and inorganic species undergo similar processes.^338^

In general, gas-phase species contribute surface mass if they undergo reactions with leaf wetness or chemicals dissolved therein. Sulfur dioxide (SO_2_) and ozone are two gases that show this behavior.^12,338−340^ SO_2_ is highly water-soluble and has long been shown to be taken up by leaf wetness via reversible acid–base chemistry (reviewed by Erisman and Baldocchi^339^). However, in the presence of oxidants like ozone, hydrogen peroxide, and O_2_ + trace metals (e.g., Mn^2+^), a fraction of this dissolved SO_2_ is irreversibly converted to sulfate and remains on the leaf after evaporation.^331,339,340^ Ozone is sparsely soluble in water and its uptake by leaf wetness involves irreversible chemical reactions.^12,338^ The identity of the compounds participating in these reactions is still unknown and may include organic and inorganic species^13,341^—in agreement with known principles of aqueous-phase ozonation^342^—of both endogenous and exogenous origin.^13^ In addition to SO_2_ and ozone, a few lines of evidence hint that aqueous ammonia may be reacting with organic compounds dissolved in leaf wetness to form new organonitrogen species. This yet untested hypothesis may help explaining why throughfall is enriched in dissolved organic nitrogen compared to incoming precipitation and justifies the ability of canopies to retain ammonia and other inorganic nitrogen species.^343−345^

Acid–base chemistry is another way through which water-soluble gases interact with leaf wetness—however, in the absence of subsequent reactions, their uptake is only temporary: gases are released back to the atmosphere when wetness evaporates.^340^ This process is well-known for ammonia^15,17,338,340^ and SO_2_^338,339^ and, more recently, has also been observed for organic acids (e.g., formic, propionic, butyric, and isocyanic acids),^4,346^ HONO,^18,281^ and, potentially, other nitrogen-containing compounds.^338^ Wetness pH and presence of neutralizing species are two key variables controlling gas uptake.^15,17,339^

## 6 Overview of Leaf Surfaces’ Chemical Landscape and Its Reactivity

This final section summarizes the Review’s main findings and contextualizes them in the broader environmental science literature. First, we discuss semiquantitative estimates of surface mass coverage for each compound class (section 6.1.1) and describe examples that better contextualize these numbers and highlight their environmental drivers (section 6.1.2). Second, we summarize known and expected surface reactivity of exogenous and endogenous chemicals with atmospheric oxidants (section 6.2.1) and provide a unified view of the dynamic multiphase reactivity we anticipate on leaf surfaces (section 6.2.2). Across the text, we highlight new frontiers for research in this evolving topic.

### 6.1 Relative Contributions of Exogenous and Endogenous Chemicals

#### 6.1.1 Expected Contributions from Different Compound Classes

This Review highlights the large number of factors influencing the leaf’s surface chemical landscape–plant species, physiology, location, and meteorological conditions, just to name a few. Despite the anticipated variability, we used available literature data to estimate order-of-magnitude surface mass contributions for each compound class and better contextualize the qualitative evidence presented above. As this analysis emphasizes observational data, our results are inherently biased—either by selected analytes (e.g., PAHs are the most commonly measured SVOCs on leaves but not necessarily the most abundant on a per-mass basis) or environmental context (e.g., pesticides are dominantly studied in agricultural systems). To overcome these limitations and provide broader context to our conclusions, we also include top-down estimates based on alternative approaches (e.g., ecosystem-scale flux measurements for total SVOCs).

Table 1 summarizes the outcome of these back-of-the-envelope calculations for the 12 classes of endogenous and exogenous species described in sections 3 and 4, respectively. Results are expressed as surface mass coverage, i.e., the mass of organic species (individual molecules or particles) per unit of leaf area (Γ_*i*_, in μg cm^–2^). To aid comparison, we report this data both as a range (Γ_*i*_^min^–Γ_*i*_^max^) and log_10_-based average (Γ̂_*i*_, where log_10_ Γ̂_*i*_ = (log_10_ Γ_*i*_^min^ + log_10_ Γ_*i*_^max^)/2). For endogenous chemicals, we further specify if results are applicable for any or selected plant species, whereas for exogenous species we clarify if they are valid in general or for particular environments. The need for this elucidation depends on input data and assumptions underpinning each estimate. The full description of equations, assumptions, limitations, and alternative approaches is presented in the Supporting Information.

**Table 1:** Estimated Contribution of Each Compound Class to the Total Organic Mass on Leaf Surfaces^a^

				Γ_*i*_ (μg cm^–2^)		
	plant type	environment	distribution	range	log_10_-based average (Γ̂_*i*_)	equation
endogenous compounds						
trichomes	B^b^		O	0.027–30	0.90	S1
guttation	B^c^		E	0.0000049–10	0.0070	S2
resins	C		E	0.00056–0.034	0.0044	S3
phyllosphere	All		E	2.0–100	14	S4
exogenous compounds, dry deposition						
PM^d^		All	O	0.20–115	4.8	S5
pollen		A^e^	O	1.5–210	18	S6
soil particles		All^f^	O/E	0.015–126	1.4	S7
SVOCs		All	O	0.28–1.7^g^	0.68	S8
PAHs^h^		All	O	0.000012–0.064	0.00088	S9
exogenous compounds, wet deposition						
rain		All	O/E	0.00000015–0.26	0.00020	S10
fog		R	O	0.000020–0.35	0.0027	S11
		U		0.00040–4.1	0.040	
pesticides		A	O	0.000014–0.35^i^	0.0022	S12

a Endogenous compounds are considered only in broadleaves (B), conifers (C), or all plant types (All), while exogenous species are found in rural/pristine areas (R), urban/polluted areas (U), in agricultural settings (A), or all environments (All). For each entry, we further specified if chemicals are spread homogeneously (O) or heterogeneously (E) across the surface; if the latter, Γ_*i*_ should be considered an *average* surface mass load. To facilitate comparison, we also report the log_10_-based average (Γ̂_*i*_) of each range. Text S2 provides a detailed description of equations, input data, derivations, and limitations of these estimates.

b Only plant species with glandular trichomes.

c Based on data for crops but in principle applicable to any broadleaf.

d Only water-insoluble components.

e Only plants inside the crop field at pollen maturity.

f Only leaves close to the soil (up to ≈50 cm).

g To be considered a reasonable order of magnitude rather than a range.

h Referred to a class of 5–15 individual PAH analogues.

i Values up to 10–20 times higher right after application.

Despite the assumptions and inherent limitations of our analysis, this exercise highlights three key findings: (1) phyllosphere and PM contributions are always predominant; (2) wet deposition is never competitive with other sources; (3) individual compounds are minor contributors to the total deposited mass of organics.

##### Biofilms and Particles Are Key to the Surface Mass Budget

Based on our estimates, phyllosphere biofilms and dry-deposited particles are the most significant contributors to the surface mass of organics, with log-based averages always >1 μg cm^–2^ and Γ_*i*_^min^ generally >0.1 μg cm^–2^. Although Table 1’s data for pollen and soil particles apply only to specific contexts (see Supporting Information), numbers for phyllosphere biofilms and generic PM have more general validity; furthermore, they are based on direct observational evidence, and we therefore deem them robust. Indeed, the main factor defining Γ_phyllosphere_ is the average bacterial cell coverage of 10^6^–10^7^ cell cm^–2^,^142^ a number that appears well-established in the literature. Likewise, Γ_PM_, Γ_pollen_, and Γ_soil_ rely on direct measurements of particles’ mass on leaves. A bias of the three latter estimates is the disproportional effect that a few large particles may have on the total deposited mass. This fact is highlighted by estimating Γ_*i*_ for the lowest PM size fraction (PM_2.5_), which yields Γ_PM2.5_ = 0.014–16 μg cm^–2^ (see Supporting Information), at least an order of magnitude lower than Γ_PM_. Still, the fact that Γ_PM_, Γ_pollen_, and Γ_soil_ fall in the same range strengthens our conclusions on the predominant contribution of particles to the total mass of organics expected on leaf surfaces.

In addition to particles, a few classes of individual compounds are quantitatively important to the total surface mass. Specifically, we expect 50–90% of the biofilm’s mass to consist of extracellular polymeric substances,^144,145^ yielding Γ_EPS_ = 1.0–90 μg cm^–2^. Thus, the ubiquitous presence of phyllosphere bacteria across environments and plant species makes EPS potentially responsible for most nonparticle mass on leaves. For selected plant species, trichome metabolites may also contribute substantially to the leaf’s chemical landscape (Γ_trichomes_ = 0.027–30 μg cm^–2^); if not in terms of mass, they will most likely dominate its surface reactivity (e.g., as observed in tobacco^120^).

##### Wet Deposition Has a Minor Role in the Direct Delivery of Chemicals

The second key finding is that wet deposition has a consistently negligible contribution to the total deposited mass, even at elevated organic carbon concentrations. Indeed, our “best-case” scenario (i.e., highest possible level of deposited mass) of fog deposition in urban areas yields Γ̂_fog_ = 0.040 μg cm^–2^, 2 orders of magnitude lower than particles and biofilms. Fog becomes noteworthy only if concentrations reach several hundreds of mg_C_ L^–1^, a situation encountered only in extremely polluted environments.^248^ Rain’s contribution also increases with pollution (e.g., in the presence of wildfires^347^) but it never becomes competitive with other sources. (Details on extreme-case estimates are in the Supporting Information.)

Despite the minor role of fog and rain in the direct delivering of chemicals, their *indirect* contributions may be substantial. A considerable limitation of our analysis is the missing account of leaching through cuticular water pores, a process that requires surface wetness to take place. Although several indirect lines of evidence point to its occurrence (sections 5.2–5.4), this mechanism is not yet explicitly recognized as a source of surface chemicals—thus, at the current state of knowledge, any Γ_*i*_ estimate would be entirely speculative. From a qualitative standpoint, we expect this process to be ubiquitous, occur in the presence of surface wetness, and release hydrophilic, low-molecular-weight compounds (e.g., carbohydrates) on the leaf surfaces. Water-soluble chemicals released from the partial dissolution of PM in surface wetness may also bring sizable contributions to the total surface mass (see Supporting Information). Confirming the occurrence of leaching through cuticular water pores and dissolution of leaf-adsorbed PM and assessing their role in shaping the leaf surface’s chemicals landscape is a main research priority.

##### Individual Compounds’ Contributions to the Total Mass Are Negligible

The third overarching observation is that *individual* compounds are quantitatively unimportant in the overall mass balance. This fact is particularly striking for pesticides applied as aqueous sprays as these products are designed to be highly concentrated. Estimated surface concentrations range from 0.000014 to 0.35 μg cm^–2^, with log-based averages of 0.0022 μg cm^–2^—more than 3 orders of magnitude lower than PM and phyllosphere biofilms. Pesticide residues can be up to 20 times higher shortly after application,^267^ but not even in this scenario log-based averages exceed 0.05 μg cm^–2^. PAHs, the semivolatile substances most often studied and detected on leaf surfaces (section 4.1.4.2), are an order of magnitude less abundant than wet-deposited pesticides. Based on typical concentrations reported in the literature, we expect negligible contributions from other SVOCs (section 4.1.4.2) and individual chemicals in fog and rain (section 4.2.1.3), whereas individual metabolites excreted by phyllosphere bacteria may contribute similarly to pesticides in aqueous sprays (Γ̂_phyllo,met_ = 0.030 μg cm^–2^; details in the Supporting Information). Overall, the limited role of individual molecules to the total deposited mass agrees with similar estimates for urban grime^24^ and common knowledge that, once in the environment, organic compounds are continuously processed to form mixtures of up to thousands of individual molecules.^332,348−350^ Of course, this estimate is mass-based and ignores toxicity or bioactivity–individual molecules may be low in mass but highly toxic, with important consequences for environmental health.

#### 6.1.2 Case Studies

The previous section provides a limited account of how biological, geographical, and meteorological variables influence the leaf surface’s chemical landscape. Indeed, most compound classes are found only in specific environments or/and selected plant species, with dynamic contributions also in the absence of surface reactivity. Here, we present two case-studies to clarify how these variables may shape the leaf surface’s chemical landscape. Although fictional, these examples illustrate how one can use available information to make educated guesses on expected chemical composition and reactivity.

**Figure 9:**
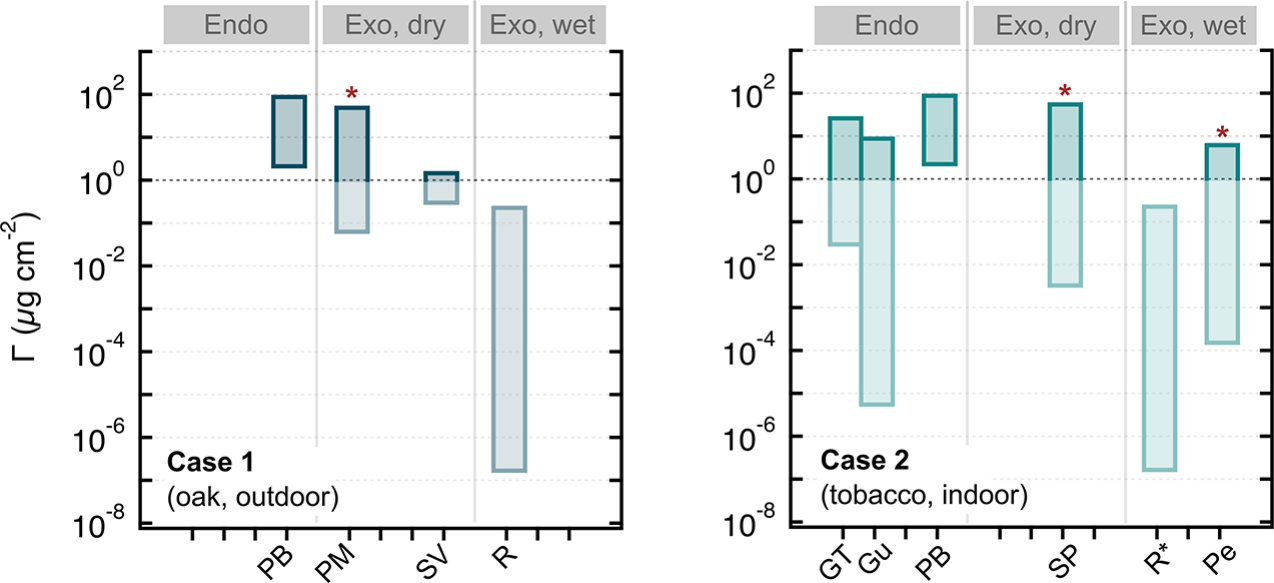
Case-studies elucidating the impact of plant species, location, and meteorological conditions on the leaf surface’s chemical landscape. The first example (left panel) considers a holm oak close to a traffic-busy road during a rainy winter day; the second (right panel) illustrates a tobacco plant treated with aqueous pesticides inside a greenhouse. For each relevant contribution, we report the estimated concentration range from to Table 1; in three cases (clarified in the text and indicated with an asterisk), these contributions are scaled to correct for specific conditions. As in Table 1, Γ_*i*_ refers uniquely to the leaf surface mass of *organic* species. Acronyms on the *x*-axis indicate glandular trichomes (GT), guttation (Gu), phyllosphere biofilm (PB), particulate matter (PM), soil particles (SP), semivolatile compounds (SV), rain (R or R*, indicating natural rain or “rain-like” contributions), and pesticides applied through aqueous sprays (Pe). Numeric data are in Table S1.

First, we consider a mature holm oak tree (*Quercus ilex*) close to a heavy-traffic road during a rainy winter day (Figure 9, left; this plant is an evergreen, so leaves are present throughout the year). In terms of endogenous substances, we expect only phyllosphere contributions to be relevant. *Q. ilex* leaves are densely covered with stellate nonglandular trichomes but lack glandular ones^94^—thus, trichome contributions can be excluded *a priori*. Likewise, Γ_resin_ = 0 because oaks are broadleaves, not conifers. Guttation is in principle possible but will not occur on a cold, rainy day (section 2.1.3). Endogenous substances leached through cuticular water pores are also likely present as rain drops maintain the leaves wet (not shown in Figure 9); for this specific example, this still unconstrained pathway likely determines how meteorological variables shape the leaf surface’s chemical landscape (see below). For exogenous substances, we anticipate potential contributions only from PM, SVOCs, and organics in rain. Our estimates for Γ_pollen_ and Γ_pesticide_ are only applicable to agricultural environments (see Supporting Information for details), whereas Γ_soil_ can be disregarded because leaves of a mature oak tree lay ≫50 cm; likewise, Γ_fog_ = 0 due to our selected meteorological conditions. Given the tree’s location and high density of nonglandular trichomes, we expect PM to be the most abundant contributor to the surface mass, even given removal by precipitation (Γ_PM_^*^ = Γ_PM_·(1 – *f*_rain_), with *f*_rain_ = 0.51–0.7;^164^ see also section 4.1.1). Overall, we anticipate Γ_tot_ = Γ_PM_^*^ + Γ_phyllosphere_ + Γ_SVOCs_ + Γ_rain_ = 2.3–158 μg cm^–2^, with predominant contributions from the phyllosphere and PM (Table S1). Notably, meteorological variables appear secondary for this combination of plant species and location–in a sunny day, Γ_tot_ = Γ_PM_ + Γ_phyllosphere_ + Γ_SVOCs_ = 2.5–217 μg cm^–2^ (Table S1). This conclusion stems directly from the missing quantification of leaching though cuticular water pores: if substantial, this contribution will drive differences in both surface mass and overall chemical composition between wet and dry days.

In a second case study, we consider a fully developed tobacco plant (*Nicotiana tabacum*) growing inside a greenhouse (Figure 9, right). We additionally suppose that the greenhouse has an efficient air filtration system (i.e., Γ_PM_ = Γ_SVOCs_ = 0), a drip irrigation system (i.e., leaves are never in contact with irrigation water), and that pesticides have been recently applied as aqueous sprays. Under these conditions, we anticipate a similar cumulative surface mass (driven by phyllosphere biofilms) but different chemical composition than the previous case-study. Trichome metabolites, phyllosphere biofilms, and, potentially, guttates and cuticular water pores leachates contribute endogenous compounds. Three trichome types have been documented on *N. tabacum* leaves, two of which are glandular; of these, only “tall” glandular trichomes excrete resinous material (including cembratrienediol and other diterpenes), whereas “short” ones function as hydathodes, releasing aqueous secretions rich in nicotine, toxic metals, and sometimes antimicrobial proteins (reviewed by Uzelac et al.^111^). As of exogenous substances, we anticipate soil particles to dominate dry deposition on leaves close to the ground. As fully grown *N*. *tobacco* plants are 1–3 m tall with leaves distributed across the whole height,^351^ we scaled Γ_soil_ to account for the fact that only 16–50% of the leaves hang less than 50 cm from the soil. Last, wet exogenous contributions include the applied pesticide, other formulation components (e.g., surfactants), and other organics present in the aqueous solvent (whose contribution we assume comparable to natural rain, and thus negligible). As we consider the situation of a *just applied* spray, we multiplied the pesticide’s contributions by 10–20,^267^ driving Γ_pesticide_^max^ > 1 μg cm^–2^. Formulations also contains surfactants, and we thus predict more leaching through cuticular water pores than with natural wetness. By summing all relevant contributions, we obtain Γ_tot_ = Γ_trichomes_ + Γ_guttation_ + Γ_phyllosphere_ + Γ_soil_^*^ + Γ_pesticide_^*^ = 2.0–210 μg cm^–2^, with major influences from the phyllosphere, soil particles (most relevant for leaves close to the soil), and trichome metabolites (Table S1). As the previous case-study, endogenous substances leached through cuticular water pores may be major drivers of the leaf surface’s chemical landscape, but their contribution remains unconstrained in our estimate. Notably, trichome metabolites are unlikely to comprise more than 15% of the total surface mass of organics but will most likely drive the leaf surface’s reactivity (at least concerning O_3_; see also section 3.2.1).

### 6.2 Observed and Expected Multiphase Reactions on Leaf Surfaces

#### 6.2.1 General Overview of Literature Findings

Beyond surface mass, the specific reactivity of leaf-deposited chemicals defines their contribution to multiphase atmospheric processes. Table 2 provides a cohesive summary of the literature presented in previous sections divided by compound class and atmospheric oxidant. This overview includes also the cuticle, as its interfacial location makes it an ideal site for multiphase chemistry. Combinations of chemicals and oxidants are marked with at least one full dot if studies showed evidence of reactivity (●, <5 studies; ●●, ≥5 studies) and with a cross (×) when reactivity was tested but not found. Asterisks highlight combinations that we anticipate based on observed reactivity in other natural or multiphase systems; together with empty spaces, they identify knowledge gaps and opportunities for future research.

**Table 2:** Summary of Multiphase Reactions for the 12 Categories of Organic Compounds and Particles Identified in This Review^a^

	atmospheric oxidant				
	O_3_	OH	*hv*	other	comments
cuticle	×			× (NO_*x*_, SO_2_)	based primarily on conifers
endogenous compounds					
trichomes	●		●		
guttation			(*)		proposed by Dibley et al.^129^
resins	*	*		* (NO_*x*_)	proposed based on reactivity of individual components in other media (section 3.3.2)
phyllosphere	*	*	*		proposed based on reactivity in other media (Sect. 3.4.2)
exogenous compounds, dry deposition					
PM	*	*	*	* (NO_*x*_)	proposed based on reactivity in other media (section 4.4.2)
pollen	*			* (NO_*x*_)	proposed based on reactivity in air (section 4.4.2)
soil particles					
SVOCs					
PAHs	*		●		proposed based on reactivity on other surfaces (section 4.4.2)
exogenous compounds, wet deposition					
rain					
fog					
pesticides	●	●	●●	● (soil dust)	

a Leaf surface reactions reported in the literature are indicated with one (●, <5 studies) or two (●●, ≥5 studies) filled dots if they take place, or with a cross (×) if the reactivity is negligible. Asterisks (*) indicate reactions that can be anticipated based on observed reactivity in the gas-phase and/or on other surfaces (“other media” refers to both), for specific chemicals of each category (e.g., abietic acid) or the whole category (e.g., PM). Both asterisks and empty spaces highlight knowledge gaps. As atmospheric oxidants, we consider ozone (O_3_), hydroxyl radicals (OH), and light (hv).

Four general trends emerge from Table 2. First, all tested chemicals *deposited onto the cuticle* show multiphase reactivity, whereas the cuticle itself is consistently unreactive. Even if this conclusion may not be valid in general (we note that the cuticle degradation literature is biased toward conifers, whose epicuticular waxes consist primarily of a saturated alcohol; see section 2.2.2), it clearly underlines the disparity between “living” and “non-living” matter. The cuticle itself is not a living structure, but its composition responds dynamically to environmental stressors.^352^ Thus, in living plants, the cuticle’s lack of multiphase reactivity is likely the result of evolutionary pressure, which resulted in the establishments of mechanisms that replace degraded components—as a matter of fact, solar radiation and microbes can degrade the cuticles of *dead* leaves (e.g., Logan et al.^353^).

Second, available information on leaf surface reactivity reflects research interests rather than environmental relevance. According to Table 2, pesticide photochemistry is the most popular topic in the leaf surface reactivity literature—in our opinion, this fact mirrors an interest in predicting pesticides loss in agricultural contexts rather than a predominance of leaf surface photochemistry in general. As light availability is a key driver for photochemistry, this reaction can be relevant for grasses, bushes, crops, and leaves in external canopy layers but less *within* the canopy (section 4.4.1). Ozone is the second most relevant multiphase oxidant, both in terms observed and expected reactivity. Unlike sunlight, ozone’s potential to undergo multiphase chemistry depends only on its background concentration, and we therefore expect leaf surface ozonolysis to be relevant in most terrestrial ecosystems. Future research on this process may also benefit from the large body of literature on wastewater ozonation^342,354^ and the growing number of investigations on multiphase indoor chemistry.^299,301^ Hydroxyl radicals have been largely overlooked despite their central role as outdoor atmospheric oxidants and broad reactivity spectrum; NO_*x*_ may also play a role, but direct empirical evidence of its involvement in leaf surface chemistry is still lacking.

Third, the role of surface wetness in mediating and/or modifying leaf surface reactivity is another significant knowledge gap that emerged from our synthesis. As leaves are wet most of their time (section 5.1) and water films impact the leaf’s chemical landscape (sections 5.2–5.4), one cannot neglect ambient relative humidity (or RH in the leaf’s boundary layer, depending on environmental conditions) when investigating leaf surface reactions. In our view, water is likely to drive differences in multiphase reactivity between leaf cuticles and other ambient indoor and outdoor surfaces (see section 6.2.2.).

Fourth, we note a lack of studies on phyllosphere biofilms and particulate matter, two compound classes we expect to dominate the leaf surface’s mass (section 6.1). Whereas PM reactivity can be inferred from the rich aerosol literature (section 4.4.2), we are not aware of multiphase studies on extracellular polymeric substances and bacterial surfaces–although we know they can react with ozone, hydroxyl radicals, and light when dissolved or suspended in water (section 3.4.2). Microbes can also act as ice nucleating particles,^355^ indicating the potential for complex surface interactions with water and, potentially, oxidants and other compounds. This ice nucleating ability is thought to contribute to frost injury in sensitive plants,^356^ but has yet to be considered in terms of multiphase reactivity.

#### 6.2.2 Toward a Unified View of Leaf Surface Reactivity

**Figure 10:**
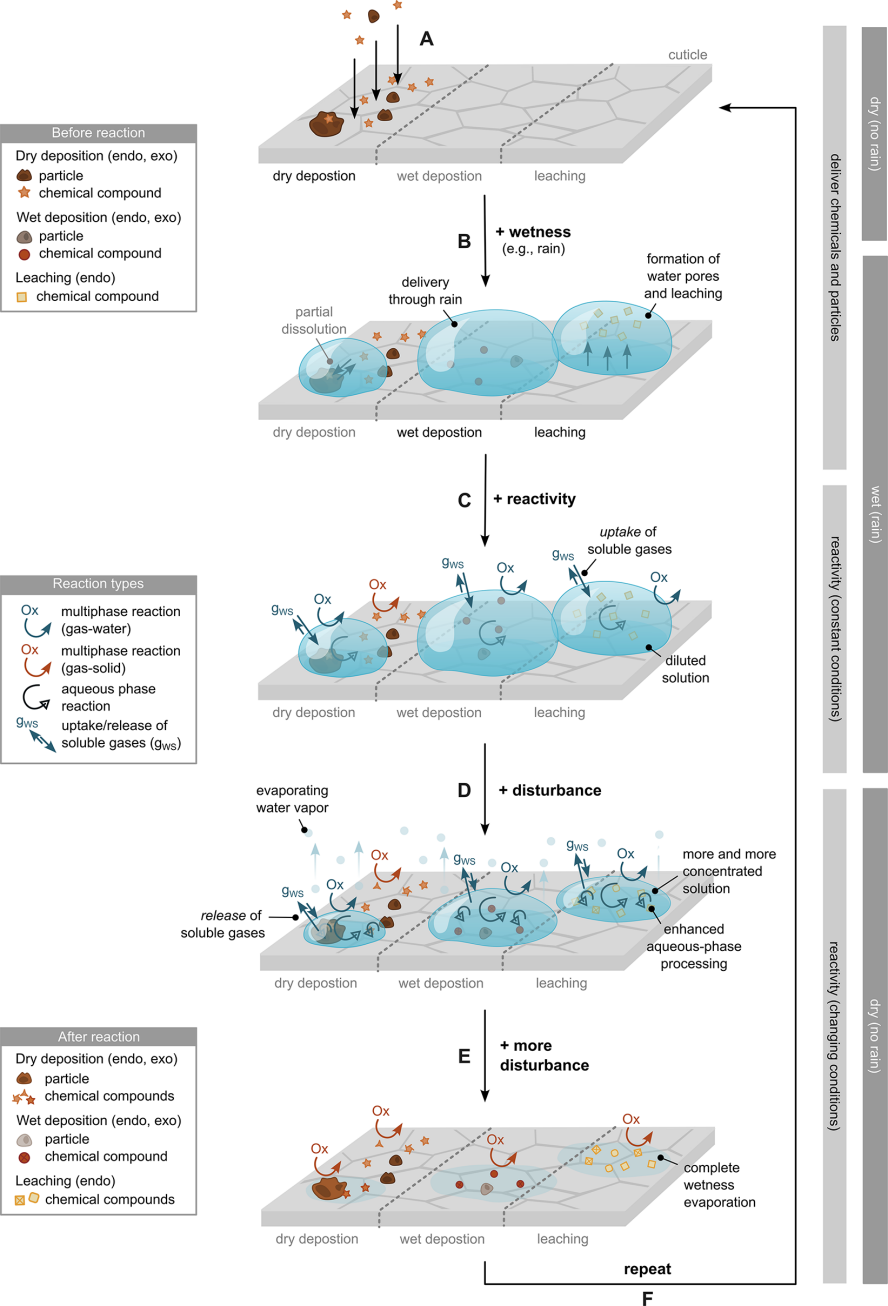
Simplified overview of the dynamic chemistry occurring on leaf surfaces. This example considers only some of the compound classes and reactions described in previous sections, namely particulate matter and SVOCs delivered through dry deposition, chemicals in rain, and endogenous chemicals leached through cuticular water pores. Reactions mediated by phyllosphere biofilms are also excluded. The gray bars on the right-hand side indicate processes (light gray) and prevailing meteorological conditions (dark gray). See section 6.2.2 for a comprehensive description of this figure.

This Review emphasizes the multifaceted and dynamic nature of the leaf surface’s chemical landscape—if biological, geographical, and meteorological factors define its overall composition (section 6.1.2), oxidant exposure and changes in surface wetness trigger multiphase reactions and partitioning of water-soluble gases. These processes further modify the chemical landscape, introducing additional layers of chemical, spatial, and temporal variability. Figure 10 illustrates a simplified overview of these processes for selected species interacting with rain and generic gas-phase oxidants. For the sake of simplicity, we start from a “pristine” cuticle with no surface wetness and no adsorbed chemicals; complexity is added stepwise by considering contributions from abiotic reactions and the effect of changing environmental conditions. Although not included in Figure 10, we expect these principles to remain valid for other forms of surface wetness, reactions (including biotic processes mediated by phyllosphere microbes; see section 3.4.2), and chemical species described in this Review. For selected combinations of plant species, chemical classes (e.g., trichome metabolites), and oxidants (e.g., sunlight), surface reactivity will most likely differ across leaf sides—however, we do not anticipate this fact to modify significantly the general cycle described below.

Particles and SVOCs are first brought onto the cuticle via dry deposition (Figure 10A; trichomes exudates and resins can also be included in this category). Then, rain adds surface wetness and supplies new species via two mechanisms (Figure 10B): (i) direct delivery of exogenous chemicals and particles and (ii) leaching of endogenous low-molecular-weight compounds via cuticular water pore formation. Dry-deposited particles may also undergo partial dissolution and release additional chemicals. We anticipate analogous processes in the presence of fog or low clouds and, potentially, when hydathodes excrete guttation drops.

After chemicals are delivered onto the leaf, surface reactivity may occur (Figure 10C)—including gas–solid (multiphase, red arrows), gas–liquid (multiphase, blue arrows), and aqueous (bulk; blue arrows with empty head) reactions. Water-soluble gases (e.g., NH_3_) will also undergo partitioning, further contributing reactants and/or modifying the solvent’s chemistry. Partitioning is favored by the dilute character of hydrometeors; in guttation drops, gas uptake may be hindered by high solute concentrations. Environmental disturbance will further impact the leaf surface chemistry (Figure 10D). In this example, we consider factors inducing surface wetness evaporation, e.g., sunlight, wind, and decreasing ambient RH. Evaporation decreases the volume of liquid water, concentrating nonvolatile chemicals and releasing water-soluble gases. The resulting changes in concentration gradient between the leaf’s interior and its surface may start favoring metabolite uptake through cuticular water pores rather than their release (not shown in Figure 10). As surface wetness dissipates, aqueous-phase reactivity (blue arrows) may speed up (e.g., due to increased concentration of reactants in second-order reactions) or change altogether–shrinking volumes modify ionic strength and pH and may foster acid-catalyzed hydrolysis and condensation reactions. We anticipate this chemistry to act on a rapid time scale (minutes to hours), reflecting typical evaporation rates observed in the environment.^229,240^

As wetness continues to evaporate, aqueous-phase reactivity becomes more and more intense until coming to a full stop; without more (macroscopic) wetness, only gas–solid reactions remain active (Figure 10E). (Under the right conditions, aqueous-phase reactivity may still be active in patches of microscopic surface wetness; not shown in Figure 10.) At the end of this first cycle, the leaf surface’s chemical landscape appears different from the beginning; and as a new cycle takes place (Figure 10F), more substances are added, processed, and removed. Conceptually, this process is analogous to the growth and evolution of organic films on the surface of inert substrates indoor^300,301^ and outdoor,^24,25^ with differences related to specific features of the substrate (cuticles vs inert materials), oxidant, and substrates availability, and prevailing reaction conditions.

Although simplified, this conceptual overview underscores the potential of leaf surface chemistry to explain some of the observations that motivated our work. For instance, interfacial ozonation of chemicals dissolved in leaf wetness may explain the nonstomatal O_3_ uptake observed when leaves are wet.^12−14^ These chemicals may originate from the dissolution of deposited aerosols or surface biofilm components and/or may leach through cuticular water pores, justifying why this nonstomatal uptake is observed also in species lacking glandular trichomes. A better understanding of leaf surface chemistry may also help refine the description of organic gases’ interaction with wet canopies. Wetness evaporation increases acidity and ionic strength, with consequent impacts for the release of water-soluble species due to shifting acid–base equilibria and salting effects. The co-occurrence of organics will also impact effective Henry’s law coefficients, in addition to providing substrates that can supply the same gases through multiphase chemistry. For example, sunlight is a driver of wetness evaporation and may trigger the photodegradation of leaf surface organics, with the consequent release of gaseous products. Wetness evaporation may also speed up acid-catalyzed reactions (e.g., hydrolysis of isoprene epoxydiols^19,20^) and enable condensation reactions—a process that may help explain the presence of chromophores and organonitrogen species in tree-DOM.^332,343,345^ Last, given its ubiquitous presence and substantial contribution to the total leaf surface mass (section 6.1), phyllosphere biofilms may be overlooked contributors to the leaf surface reactivity, promoting both biotic and abiotic (redox) chemistry (section 4.4.2). Collectively, these yet untested hypotheses highlight the potential of leaf surface chemistry as an emerging topic in several environmental science disciplines.

## 7 Conclusions

Drawing from the broad natural science literature, this Review provides unequivocal evidence for the presence of a rich blend of organics on leaf surfaces. Although the specific amount and chemical composition are shaped by a complex interplay of biological, geographical, and meteorological factors, our back-of-the-envelope calculations indicate that cumulative mass loads are substantial (≫2 μg cm^–2^) and likely driven by phyllosphere biofilms, dry-deposited particles, and, potentially, leaching of endogenous chemicals in the presence of surface wetness. Globally, these concentrations scale to ≫3 Tg of organic material available for multiphase chemistry (details in the Supporting Information), underscoring the potential of the leaf surface’s chemical landscape for impacting key atmospheric processes–including dry ozone deposition and the formation of secondary organic aerosols. We hope our work will spark a renewed interest in multidisciplinary research leveraging experimental, analytical, and conceptual expertise across natural science disciplines—from environmental and analytical chemistry to plant sciences, biochemistry, microbiology, and ecology.

## Supporting Information Available

Details on the chemical composition of *Fagus sylvatica* leaf cuticles (Text S1), estimates of individual classes’ contribution to the total organic mass deposited on leaf surfaces (Text S2), global estimate of the mass of organics on plant leaves (Text S3), and numeric data used for Figure 9 (Table S1) (PDF)

## Abbreviations list

DRH: deliquescence relative humidity
EPS: extracellular polymeric substances
*E*_i_/*P*: fraction of precipitation lost to evaporation at the top of the canopy
*f*: interception factor
HONO: nitrous acid
HNO_3_: nitric acid
*K*_OA_: octanol–air partition coefficient
*K*_plant-air_: plant–air partition coefficient
NH_3_: ammonia
NO_*x*_: nitrogen oxides
NO_2_: nitrogen dioxide
O_3_: ozone
OH: hydroxyl radical
PAHs: polycyclic aromatic hydrocarbons
PFAS: perfluoroalkyl substances
PM: particulate matter
PM_2.5_: particulate matter (<2.5 μm)
POPs: persistent organic pollutants
RH: relative humidity
RH_amb_: ambient relative humidity
RH_leaf_: relative humidity in the leaf boundary layer
*S*: canopy water storage capacity
SO_2_: sulfur dioxide
SVOCs: semivolatile organic compounds
tree-DOM: tree dissolved organic matter
Γ_*i*_: mass of organic per unit of leaf surface area (range)
Γ̂_*i*_: mass of organic per unit of leaf surface area (log_10_-based average)
