# Supplementary material for: The Chemical Landscape of Leaf Surfaces and Its Interaction with the Atmosphere
Source: Chem Rev. 2024 Apr 23;124(9):5764–94. doi: 10.1021/acs.chemrev.3c00763 (PMC11082906; doi:10.1021/acs.chemrev.3c00763)
Supplement: Supplementary file 1 — cr3c00763_si_001.pdf [file cr3c00763_si_001.pdf]

Supplementary Materials for

The chemical landscape of leaf surfaces and its interaction with the atmosphere

Rachele Ossola* and Delphine Farmer*

Department of Chemistry, Colorado State University, 80523 Fort Collins, Colorado (USA)

* Correspondence to: rachele.ossola@colostate.edu and delphine.farmer@colostate.edu

Table of Contents

Text S1: Details on the chemical composition of *Fagus sylvatica* leaf cuticles (Figure 2).......... S2

Text S2: Details on estimates of organic mass deposited on leaf surfaces.................................. S3

Table S1: Estimated contribution of individual compound classes to Γ_{tot} for the case-studies in Section 6.1.2 and Figure 9. .... S18

Text S3: Details on global extrapolation of organic mass on leaf surfaces S19

References... S20

Text S1: Details on the chemical composition of *Fagus sylvatica* leaf cuticles (Figure 2)

Cutin monomers Depolymerization of *Fagus sylvatica* leaf cuticles yields various substituted C_{16} and C_{18} ω-hydroxycarboxylic acids and trace amounts of 1-alkanols, for a total of 33.3 μg cm^{-2} of material.[1] 8,16- and 9,16-dihydroxy-hexadecanoic acid are the major C_{16} analogues (11.0 μg cm^{-2}), while 16-hydroxy-hexadecanoid acid is present only in trace amounts (0.9 μg cm^{-2}). Among C_{18} hydroxy acids, 9,10-epoxy-18-hydroxy-oxadecanoic acid is the most abundant component (7.7 μg cm^{-2}), followed by trace amounts of 18-hydroxy-oxadecenoic acid (2.0 μg cm^{-2}) and 9,10,18-trihydroxy-oxadecanoic acid (1.3 μg cm^{-2}). The authors also report substantial amounts (29.6 μg cm^{-2}) of unsubstituted monobasic alkanoic acids (e.g., octadecenoic acid), but they attribute their presence to an extraction artifact.

Cuticular waxes Cuticular waxes are typically isolated by dipping plant leaves into dichloromethane. A rapid dip (1 – 3 min; e.g., Gülz et al.[2,3]) yields the epicuticular component, while a longer one (20 – 30 min, sometimes accompanied to a gentle heating; e.g., Naß et al.[4]) extracts both epi- and intracuticular waxes. Both methods have been applied to investigate the wax composition of *F. sylvatica* cuticles, showing good *qualitative* agreement but differences in the amounts of some classes. For example, Gülz et al.[2] isolated 15 μg cm^{-2} of epicuticular waxes, including *n*-alkanols (34.8%), alkyl esters (17.4%), *n*-alkanes (17.0%), *n*-aldehydes (10.3%), *n*-alkanoic acids (8.1%), and trace amounts of benzyl acid esters (0.9%). C_{28} and C_{27} analogues were generally the most abundant. Specifically, C_{27} was predominant among *n*-alkanes (89.5% of all detected *n*-alkanes), while C_{28} was the main isomer among *n*-aldehydes (73.4%), benzyl acid esters (64%), and *n*-alkanoic acids (39.7%). Authors using the longer extraction protocol also found a predominance of C_{28} or C_{27} analogues but variations in the relative contributions of each class.[4,5] For instance, Reynhardt and Riederer[5] found a predominance of *n*-alkanols (23.2%) followed by alkyl esters (20.6%), *n*-alkanoic acids (19.7%), *n*-aldehydes (18.6%), and *n*-alkanes (17.9%) using a 30 min extraction procedure. Another study reported minor quantities of benzyl acid esters and coumaryl esters; in both cases, the C_{28} chain was the most common.[4]

Text S2: Details on estimates of organic mass deposited on leaf surfaces

General remarks

For each class of chemical or particle described in the main text, we report the equation for the mass surface coverage of organics (Γ_i in μg cm^{-2}), the ranges of values used as numerical inputs, and a summary of the main approximations underpinning our estimate. When the equation is not straightforward, we also provide its derivation; in some cases, we present additional or alternative estimates ("Additional considerations").

As we aimed for order-of-magnitude estimates applicable to diverse environments, plant types, and atmospheric conditions, we typically report Γ_i as a broad range of values ($\Gamma_i^{\min}$ to $\Gamma_i^{\max}$). The limits of this range are defined by the lowest and highest values for all variables (for variables at the denominator, we use the minimum value for the upper limit and the maximum for the lower limit), while conversion factors, physical constants, and variables for which we report typical or average values are kept constants. For chemicals heterogeneously distributed across the surface (e.g., phyllosphere biofilms), we report only the *average* mass surface coverage ($\overline{\Gamma}_i$). Our estimates ignore inorganic chemicals. For all chemical classes, we additionally report the $\log_{10}$-based average ($\hat{\Gamma}_i$), with $\log_{10}\hat{\Gamma}_i = \left(\log_{10}\Gamma_i^{\min} + \log_{10}\Gamma_i^{\max}\right)/2$.

Endogenous compounds

Metabolites from glandular trichomes

$$\Gamma_{\text{trichome}} = f_{\text{metabolite mass}} \cdot \text{LMA} \cdot f_{\text{surface metabolite}} \cdot 10^2 \qquad \text{(S1)}$$

The surface concentration of trichome metabolites (Γ_{trichome}, in μg cm^{-2}) is obtained from the product of the mass fraction of trichome metabolites to the total leaf's dry weight ($f_{\text{metabolite mass}} = 0.003 - 0.3$, in $\text{g}_{\text{metabolite}}\ \text{g}_{\text{leaf,DW}}^{-1}$),[6] the leaf dry mass per unit of surface area ($\text{LMA} = 30 - 330\ \text{g}_{\text{leaf,DW}}\ \text{m}_{\text{leaf}}^{-2}$),[7] and the estimated fraction of metabolite exposed on the trichome's surface ($f_{\text{surface metabolite}} \approx 3 \cdot 10^{-3}$; see Derivation). The multiplier accounts for units' conversion ($10^2 = 10^6\ \mu\text{g g}^{-1} \cdot 10^{-4}\ \text{m}^2\ \text{cm}^{-2}$).

Equation (S1) is based on the assumption that trichome heads are spheres of radius $r_{\text{trichome}} \approx 10\ \mu\text{m}$, that their surface is covered completely by a monolayer of metabolite of thickness $h_{\text{monolayer}} \approx 10\ \text{nm}$, and that secondary metabolites make 50% the trichome head's mass.

<u>Derivation</u> We assume that the leaf surface is covered by glandular trichomes with a spheric head of radius r_{trichome}. Each head is covered by a homogeneous monolayer of molecules of thickness $h_{\text{monolayer}}$ and total volume $V_{\text{monolayer}}$.

$$V_{\text{monolayer}} = \frac{4}{3}\pi r_{\text{trichome}}^3 - \frac{4}{3}\pi\left(r_{\text{trichome}} - 10^{-3} \cdot h_{\text{monolayer}}\right)^3, \quad \text{(S1.1)}$$

where $10^{-3} = \mu\text{m nm}^{-1}$ accounts for the different units of r_{trichome} (µm) and $h_{\text{monolayer}}$ (nm). We can assume that the mass of the surface monolayer is proportional to the ratio between the total volume of the trichome head ($V_{\text{trichome}} = \frac{3}{4}\pi r_{\text{trichome}}^3$) and $V_{\text{monolayer}}$.

$$\frac{m_{\text{monolayer}}}{m_{\text{trichome}}} \approx \frac{r_{\text{trichome}}^3 - \left(r_{\text{trichome}} - 10^{-3} \cdot h_{\text{monolayer}}\right)^3}{r_{\text{trichome}}^3} \approx 6 \times 10^{-3} \quad \text{(S1.2)}$$

If we hypothesize that only 50% of $m_{\text{monolayer}}$ consists of excreted metabolites, $f_{\text{surface metabolite}} = 6 \cdot 10^{-3} \cdot 0.5 = 3 \cdot 10^{-3}$.

Metabolites from guttation fluids

$$\overline{\Gamma_{\text{guttation}}} = \frac{\pi}{6} \cdot \frac{d_{\text{drop}}^3 \cdot N_{\text{drop}} \cdot (C_{\text{drop}}^{\text{sugar}} + C_{\text{drop}}^{\text{protein}})}{A_{\text{leaf}}} \cdot f_{\text{drop}} \quad \text{(S2)}$$

The average leaf surface concentration of guttation metabolites ($\overline{\Gamma_{\text{guttation}}}$, in µg cm^{-2}) is calculated from the average diameter of guttation drops ($d_{\text{drop}} \approx 0.5$ mm),[8] the number of guttation drops per leaf ($N_{\text{drop}} = 1 - 4$),[8] the concentration of sugars and proteins in guttation drops ($C_{\text{drop}}^{\text{sugar}} = 0.0271 - 1500$ g L^{-1} and $C_{\text{drop}}^{\text{protein}} = 0.0027 - 30$ g L^{-1}),[8] the fraction of leaves with drops ($f_{\text{drop}} = 0.05 - 0.5$),[8] and a typical leaf surface area ($A_{\text{leaf}} \approx 20$ cm^2)[9,10].

This calculation is based on experimental and literature data for crops and small bushes summarized in Urbaneja-Bernat et al.[8]; additionally, it considers only organic compounds and

assumes that 100% of guttation drops remain on the surface until evaporation. We further acknowledge that guttation metabolites are heterogeneously distributed across the leaf surface (as drops don't spread their content across the whole leaf) and the plant (as not all leaves have drops). Last, this calculation hints that primary metabolites (i.e., sugars and proteins) constitute most of the excreted mass; of these, sugars account for 90.5 – 98.0% of the total mass (based on data in Urbaneja-Bernat et al.[8]).

Derivation The mass of metabolites in a single guttation drop ($m_{\text{guttation}}$) is estimated from the drop's concentration ($C_{\text{drop}} = C_{\text{drop}}^{\text{sugar}} + C_{\text{drop}}^{\text{protein}}$, in g L^{-1}) and volume ($V_{\text{drop}} = \frac{\pi}{6} d_{drop}^3$, assuming that guttation drops are spherical).

$$m_{\text{guttation}} = \frac{\pi}{6} d_{drop}^3 C_{\text{drop}} \tag{S2.1}$$

If each leaf has N_{drop} drops, the total mass of metabolites per unit area is $m_{\text{guttation}} N_{\text{drop}} / A_{\text{leaf}}$. As not all leaves have drops, we additionally multiply this expression by f_{drop}, arriving to equation (S2).

Resins

$$\overline{\Gamma_{\text{resin}}} = \frac{\text{MW}_{\text{AA}}}{A_{\text{AA}}} \cdot \frac{d_{\text{bead}}^2}{A_{\text{leaf}}} \cdot \frac{2\pi \cdot 10^{20}}{N_A} \tag{S3}$$

The average resin mass surface concentration ($\overline{\Gamma_{\text{resin}}}$, in µg cm^{-2}) is estimated from the molecular weight ($\text{MW}_{\text{AA}} = 302.5$ g mol^{-1}) and topologic polar surface area ($A_{\text{AA}} = 37.3$ Å^2 molecule^{-1})[11] of abietic acid, resin bead diameters reported by Eller et al.[12] for ponderosa pine needles ($d_{\text{bead}} \approx 1 - 2$ mm), typical leaf surface areas for conifers ($A_{\text{leaf}} \approx 1 - 15$ cm^2),[13] the Avogadro number ($N_A = 6.023 \times 10^{23}$ molecule mol^{-1}), and a unit conversion factor ($10^{20} = 10^6\ \mu\text{g g}^{-1} \cdot 10^{16}\ \text{Å}^2\ \text{cm}^{-2} \cdot 10^{-2}\ \text{cm}^2\ \text{mm}^{-2}$).

This estimate is fully theoretical and assumes that resin beads are composed entirely of abietic acid, a common non-volatile resin component,[14,15] and that each needle has one bead (based on Eller et al.[12]). To estimate mass, we consider only the monolayer of abietic acid present on the

surface of each bead; then, we divide this number by the *total* area of a needle, yielding an *average* surface cover. Details are provided below.

Derivation The mass of a monolayer of abietic acid (m_{AA}) on the surface of a sphere of diameter d_{bead} is calculated as

$$m_{\mathrm{AA}} = \frac{N_{\mathrm{AA}} \mathrm{MW}_{\mathrm{AA}}}{N_A}, \tag{S3.1}$$

where N_A is the Avogadro number, N_{AA} is the number of abietic acid molecules on the surface of the sphere, and $\mathrm{MW}_{\mathrm{AA}}$ abietic acid's molecular weight. The maximum N_{AA} value is constrained by the ratio of the total surface area of the resin bead (A_{bead}) to that of an individual molecule (A_{AA}). (As abietic acid is rather flat, we count only one side; thus, we consider $A_{\mathrm{AA}}/2$).

$$N_{\mathrm{AA}} = \frac{A_{\mathrm{bead}}}{A_{\mathrm{AA}}/2} = \frac{2\pi \cdot d_{\mathrm{bead}}^2}{A_{\mathrm{AA}}} \tag{S3.2}$$

By combining equations (S3.1) – (S3.2) and accounting for units' conversion, one gets to the following expression.

$$m_{\mathrm{AA}} = \frac{\mathrm{MW}_{\mathrm{AA}}}{A_{\mathrm{AA}}} \cdot d_{\mathrm{bead}}^2 \cdot \frac{2\pi \cdot 10^{20}}{N_A} \tag{S3.3}$$

The input of equation (S3.3) in $\overline{\Gamma_{\mathrm{resin}}} = m_{\mathrm{AA}}/A_{\mathrm{leaf}}$ yields equation (S3).

Phyllosphere

$$\overline{\Gamma_{\mathrm{phyllo,biofilm}}} = \frac{\overline{\Gamma_{\mathrm{cell,b}}} m_{\mathrm{b}}}{1 - f_{\mathrm{C,EPS}}} \cdot 10^{-6} \tag{S4}$$

The average surface concentration of phyllosphere biofilms ($\overline{\Gamma_{\mathrm{phyllo,biofilm}}}$, in μg cm^{-2}) is obtained from the average cell surface coverage of phyllosphere bacteria ($\overline{\Gamma_{\mathrm{cell,b}}} = 10^6 - 10^7$ cell cm^{-2}),[16] the standard mass of an individual bacterium ($m_{\mathrm{b}} \approx 1$ pg $cell^{-1}$),[17,18] and typical fractions of extracellular polymeric substances (EPS) in living biofilms ($f_{\mathrm{C,EPS}} = 0.5 - 0.9$, based on carbon content)[19]. The multiplier accounts for units' conversion ($10^{-6} = \mu\mathrm{g\,pg}^{-1}$).

Our main assumption is that bacteria are the most relevant microorganisms to the mass budget. This statement is justified by a back-of-the-envelope calculation showing that yeasts, the second most abundant group,[20,21] contribute to only a minimal extent to the total phyllosphere mass. Indeed, taking $\overline{\gamma_{\mathrm{cell,y}}} = 10^3 - 10^5$ cell $\mathrm{g_{leaf,DW}^{-1}}$, an average yeast mass (m_y) of ≈ 50 pg cell^{-1},[22–24] and a leaf mass per surface area (LMA) of $30 - 330\ \mathrm{g_{leaf,DW}\ m^{-2}}$,[7] one gets $\overline{\gamma_{\mathrm{cell,y}}} m_y \cdot \mathrm{LMA} \cdot 10^{-10} = 0.00015 - 0.165\ \mu\mathrm{g\ cm^{-2}}$, which is larger than contributions from bacterial cells ($\overline{\Gamma_{\mathrm{cell,b}}} m_{\mathrm{b}} \cdot 10^{-6} = 1 - 10\ \mu\mathrm{g\ cm^{-2}}$). Additionally, we suppose that bacteria and EPS have similar elemental compositions, that EPS are more abundant than any other compound phyllosphere bacteria may excrete (further justified in "Additional considerations"), and that phyllosphere biofilms have the same EPS-to-bacteria ratio of biofilm growing on other surfaces. The derivation of equation (S4) is provided below.

Derivation The biofilm mass on a leaf is the sum of the masses of bacteria (m_{bacteria}) and extracellular polymeric substances (m_{EPS}).

$$m_{\mathrm{biofilm}} \approx m_{\mathrm{bacteria}} + m_{\mathrm{EPS}}\,, \tag{S4.1}$$

where the total mass of bacteria on a leaf (in µg) is obtained as the product of the average cell surface coverage ($\overline{\Gamma_{\mathrm{cell,b}}}$), the mass of an individual bacterium (m_{b}), the leaf area (A_{leaf}), and a unit conversion factor ($10^{-6} = \mu\mathrm{g\ pg^{-1}}$).

$$m_{\mathrm{bacteria}} = \overline{\Gamma_{\mathrm{cell,b}}} m_{\mathrm{b}} A_{\mathrm{leaf}} \cdot 10^{-6} \tag{S4.2}$$

If we assume that bacteria and EPS have a similar carbon content (i.e., $m_{\mathrm{C,bacteria}}/m_{\mathrm{bacteria}} \approx m_{\mathrm{C,EPS}}/m_{\mathrm{EPS}}$), then

$$m_{\mathrm{C,biofilm}} \approx m_{\mathrm{C,bacteria}} + m_{\mathrm{C,EPS}}\,. \tag{S4.3}$$

The contribution of extracellular polymeric substances to the total biofilm carbon content ($f_{\mathrm{C,EPS}} = m_{\mathrm{C,EPS}}/m_{\mathrm{C,biofilm}}$) is known from the literature.[19] We can thus substitute $m_{\mathrm{C,EPS}} = f_{\mathrm{C,EPS}} m_{\mathrm{C,biofilm}}$ in equation (S4.3) and solve for $m_{\mathrm{C,biofilm}}$, obtaining equation (S4.4).

$$m_{\mathrm{C,biofilm}} \approx \frac{m_{\mathrm{C,b}}}{1 - f_{\mathrm{C,EPS}}} \tag{S4.4}$$

Since m_{C}/m is constant, the same expression remains valid for the total mass. By substituting equation (S4.2) into the resulting expression, one obtains

$$m_{\mathrm{biofilm}} \approx \frac{\overline{\Gamma_{\mathrm{cell,b}}} m_{\mathrm{b}} A_{\mathrm{leaf}} \cdot 10^{-6}}{1 - f_{\mathrm{C,EPS}}}, \tag{S4.5}$$

which leads to equation (S4) after rearranging $m_{\mathrm{biofilm}}/A_{\mathrm{leaf}} = \overline{\Gamma_{\mathrm{phyllo,biofilm}}}$.

<u>Additional considerations.</u> In Table 1, we report only $\overline{\Gamma_{\mathrm{phyllo,biofilm}}}$ because bacteria and EPS contribute to a similar extent to the total deposited mass. This fact stems directly from $f_{\mathrm{C,EPS}} = 0.5 - 0.9$[19] and hints that EPS are the most relevant chemicals phyllosphere bacteria contribute to in terms of leaf surface mass. This observation is further supported by an alternative estimate for the average surface concentration of *other* metabolites excreted by bacteria cells ($\overline{\Gamma_{\mathrm{phyllo,met}}}$), which we obtained via equation (S4.6).

$$\overline{\Gamma_{\mathrm{phyllo,met}}} = \underbrace{\overline{\Gamma_{\mathrm{phyllo,biofilm}}} \cdot \left(1 - f_{\mathrm{C,EPS}}\right)}_{\Gamma_{\mathrm{phyllo,bacteria}}} \cdot f_{\mathrm{b,dry}} \cdot f_{\mathrm{b,excreted}} \tag{S4.6}$$

This calculation assumes that each bacterial cell is made $\approx 70\%$ of water ($f_{\mathrm{b,dry}} \approx 0.30$),[17,18] and that bacteria excrete only a small fraction of this mass on the leaf surface ($f_{\mathrm{b,excreted}} \approx 0.01 - 0.1$). Equation (S4.6) yields $\overline{\Gamma_{\mathrm{phyllo,met}}} = 0.0006 - 1.5\ \mathrm{\mu g\ cm^{-2}}$.

Exogenous compounds, dry deposition

Particulate matter

$$\Gamma_{\mathrm{PM}}^{\mathrm{org,NWS}} = \Gamma_{\mathrm{PM}}^{\mathrm{NWS}} \cdot f_{\mathrm{org}} \tag{S5}$$

The surface concentration of non-water-soluble organics (NWS) in particulate matter ($\Gamma_{\mathrm{PM}}^{\mathrm{org,NWS}}$, in µg cm^{-2}) is calculated from the empirical PM concentrations on leaf surfaces ($\Gamma_{\mathrm{PM}}^{\mathrm{NWS}} = 1 - 128\ \mathrm{\mu g\ cm^{-2}}$; Figure S1, left panel)[25] and the fraction of organics in submicron aerosols ($f_{\mathrm{org}} = 0.2 - 0.9$)[26].

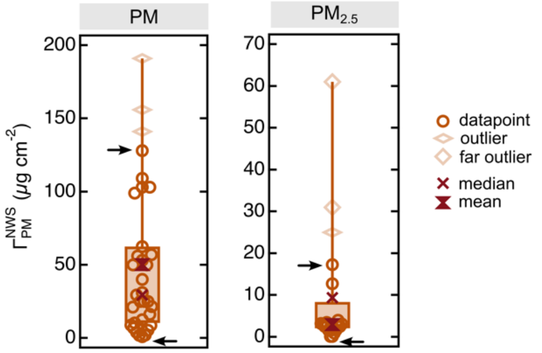

Figure S1 Overview of data used for selecting the ranges for Γ_{PM}^{NWS} (left panel) and $\Gamma_{PM2.5}^{NWS}$ (right panel). Values are replotted from Corada et al.[25] (data from Table 4; for PM, we considered only data classified as PM, sPM, PM*, and other data in units of mass per surface area; for $PM_{2.5}$, we use only data reported as $PM_{2.5}$ or $PM_{\leq 3}$ and arbitrarily set all data indicated as < 5 µg cm^{-2} to 2.5 µg cm^{-2}). The range is taken as minimum and maximum excluding outliers (black arrows; N = 34 and 19 for PM and $PM_{2.5}$, respectively). The range reported in the main text for PM includes the upper outlier (191 µg cm^{-2}). Data was plotted with Igor Pro 8 (version 8.04).

Our main assumption is that f_{org} for submicron (total) aerosols is the same for other size fractions and is valid also for the water-*insoluble* fraction. Furthermore, our estimate does not consider potential variations in Γ_{PM}^{NWS} as a function of plant type and background pollution. A recent meta-analysis showed that average deposition rates are significantly different but within a factor of 1.5 – 2.5 for grass vs. broadleaves vs. conifers and for rural vs. residential vs. industrial areas.[27] In contrast, Γ_{PM}^{NWS} increased almost 10-fold as a function of time after rain,[27] hinting than meteorological conditions may be more influential than location or plant type.

Additional considerations As the Γ_{PM}^{NWS} values used in our estimates were obtained gravimetrically (i.e., by immersing leaves in water, filtering the resulting suspension, and weighting the dried filter), they represent only organic and inorganic components that are insoluble in water. We can estimate the proportion of water soluble (WS) organics associated with these insoluble particles as $\Gamma_{PM}^{org,WS} = \Gamma_{PM}^{NWS} \cdot f_{WSOC}(1 - f_{WSOC})^{-1}$, where $f_{WSOC} \approx 0.6$ is the average ratio between water soluble organic carbon and total organic carbon in aerosols.[28] This calculation yields $\Gamma_{PM}^{org,WS} = 0.30 - 173\ \mu g\ cm^{-2}$.

An additional bias is the disproportionate large effect that large particles have on the total deposited mass. Indeed, we noticed that experimental surface coverages for $PM_{2.5}$ are generally < 5 µg cm^{-2} ($\Gamma_{PM2.5}^{org,NWS} = 0.11 - 17$ µg cm^{-2}; Figure S1, right panel), resulting in $\Gamma_{PM2.5}^{org,NWS} = \Gamma_{PM2.5}^{NWS} \cdot f_{org} = 0.022 - 16$ µg cm^{-2} (via equation (S5)). In support of this result, we

assessed $PM_{2.5}$ mass load using global dry deposition rates on vegetation from Feng et al.[29], yielding $\Gamma_{\mathrm{PM2.5}}^{\mathrm{org}} = 0.014 - 1.4$ µg cm^{-2}. This range was calculated according to equation (S5.1) using yearly global deposition rates on vegetation ($|F_{\mathrm{PM2.5}}| = 0.17 - 0.77$ g m^{-2} yr^{-1}; extracted from Fig. 5 of Fang et al.[29] using WebPlotDigitizer[30]), a range of leaf area index across biomes ($\mathrm{LAI} = 1.31 - 6.34\ \mathrm{m_{leaf}^{2}\ m_{soil}^{-2}}$; values are minimum and maximum of averages for various vegetation types),[31] $f_{\mathrm{org}} = 0.2 - 0.9$,[26] and a unit conversion factor ($10^2 = 10^6 \mu\mathrm{g\ g^{-1}} \cdot 10^{-4}\mathrm{m^2\ cm^{-2}}$). This calculation further assumes an overall $PM_{2.5}$ accumulation time of 10 days (based on Cai et al.[27]).

$$\Gamma_{\mathrm{PM2.5}}^{\mathrm{org}} = \frac{|F_{\mathrm{PM2.5}}| \cdot 10}{\mathrm{LAI} \cdot 365} \cdot f_{\mathrm{org}} \cdot 10^2 \qquad \text{(S5.1)}$$

Last, because of the way it is measured, $\Gamma_{\mathrm{PM}}^{\mathrm{NWS}}$ comprises any type of particle, including pollen and soil. We highlight that our upper estimate for PM is lower than that of either pollen or soil, reflectingly primarily a difference in environmental conditions under which source data were collected (i.e., PM data are primarily referred to woody plants in polluted urban environments, whereas the pollen and soil data are valid uniquely for small plants in agricultural environments or grasslands and specific times of the year).

Pollen

$$\Gamma_{\mathrm{pollen}} = \Gamma_{\#,\mathrm{pollen}}\, m_{\mathrm{pollen}} \qquad \text{(S6)}$$

The surface mass concentration of pollen (Γ_{pollen}, in µg cm^{-2}) is computed from its surface number concentration ($\Gamma_{\#,\mathrm{pollen}} = 9.7 - 426$ grain cm^{-2})[32] and the average mass of a corn pollen grain ($m_{\mathrm{pollen}} = 0.15 - 0.5$ µg grain^{-2})[33].

This calculation is based on the $\Gamma_{\#,\mathrm{pollen}}$ range reported in Table 1 of Pleasants et al.[32] (only corn pollen) and is applicable for plants *within* the corn field during anthesis (i.e., pollen maturity). Thus, it must be considered an upper limit estimate valid only in agricultural environments and specific times of the year.

Additional considerations For a more generic estimate, we combined our $\Gamma_{\mathrm{PM}}^{\mathrm{NWS,org}}$ values with the estimated bioaerosol fraction of in $PM_{2.5}$ and PM_{10} from Hyde and Mahalov[34] ($f_{\mathrm{pollen}} \approx 0.16$), yielding $\Gamma_{\mathrm{pollen}} = \Gamma_{\mathrm{PM}}^{\mathrm{NWS,org}} \cdot f_{\mathrm{pollen}} = 0.032 - 18$ µg cm^{-2}.

Soil particles

$$\Gamma_{\mathrm{soil}} = \gamma_{\mathrm{soil}} \cdot f_{\mathrm{SOC/SM}} \cdot \frac{\mathrm{SPB}}{\mathrm{LAI}} \cdot 10^2 \quad \text{(S7)}$$

The surface mass concentration of organics from soil particles (Γ_{soil}, in µg cm^{-2}) is obtained from the surface concentration of soil per gram of plant ($\gamma_{\mathrm{soil}} = 1.1 - 260\ \mathrm{mg_{soil}\ g_{plant,DW}^{-1}}$),[35] the average soil organic matter content ($f_{\mathrm{SOC/SM}} = 0.01 - 0.05$),[36] typical values for the standing plant biomass ($\mathrm{SPB} = 0.05 - 0.35\ \mathrm{kg_{plant,DW}\ m_{soil}^{-2}}$),[37,38] and the average leaf area index for crops ($\mathrm{LAI} = 3.6\ \mathrm{m_{leaf}^{2}\ m_{soil}^{-2}}$).[31] The multiplier accounts for units' conversion ($10^2 = 10^3\ \mathrm{\mu g_{soil}\ mg_{soil}^{-1}} \cdot 10^3\ \mathrm{g_{plant}\ kg_{plant}^{-1}} \cdot 10^{-4}\ \mathrm{m^2\ cm^{-2}}$).

This calculation is based on γ_{soil} reported for leafy crops and grasses and it is valid only for leaves growing close to the soil (generally up to 50 cm). Thus, to a first approximation, we neglect Γ_{soil} in trees, both broadleaves and conifers.

Semi-volatile compounds

For this compound class, we report two different estimates. The first is a top-down assessment using dry deposition fluxes for oxidized biogenic volatiles (F_{OBVOCs}), which we consider representative of SVOCs as a class (equation (S8)). The second is a bottom-up evaluation using experimental surface coverages for PAHs, the most commonly detected class of SVOCs on leaf surfaces (equation (S9)).[39] Given the paucity of data for F_{OBVOCs}, Γ_{SVOCs} should be taken as a representative order-of-magnitude value rather than a range.

Top-down estimate

$$\Gamma_{\mathrm{SVOCs}} = \frac{|F_{\mathrm{OBVOCs}}|}{\mathrm{LAI}} \cdot \mathcal{C}^{\mathrm{MW}} \cdot \mathcal{C}^{\mathrm{time}} \cdot 10^{-4} \quad \text{(S8)}$$

The total surface concentration of SVOCs (Γ_{SVOCs}, in µg cm^{-2}) is obtained from reported deposition rates of oxidized biogenic volatile compounds ($|F_{\mathrm{OBVOCs}}| = 266 - 1058$ µg$_{\mathrm{C}}$ m^{-2} h^{-1}; extracted from Fig. 2D of Park et al.[40] using WebPlot Digitizer[30]), the leaf area index for this specific orchard ($\mathrm{LAI} = 3.0\ \mathrm{m}^2_{\mathrm{leaf}}\ \mathrm{m}^{-2}_{\mathrm{soil}}$),[41] and conversion factors for time ($\mathcal{C}^{\mathrm{time}} = 24$ h day^{-1}), mass ($\mathcal{C}^{\mathrm{MW}}$, in $\mu\mathrm{g}\ \mu\mathrm{g}_{\mathrm{C}}^{-1}$), and other units ($10^{-4} = \mathrm{m}^2\mathrm{cm}^{-2}$). This estimate uses $\mathcal{C}^{\mathrm{MW}}$ for two representative OBVOCs, namely isoprene hydroxy hydroperoxide (ISOPOOH, $C_5H_{10}O_3$; $\mathcal{C}^{\mathrm{MW}} = 118\ \mu\mathrm{g}\ \mathrm{mol}^{-1}/(5 \cdot 12\ \mu\mathrm{g}_{\mathrm{C}}\ \mathrm{mol}^{-1}) = 1.97\ \mu\mathrm{g}\ \mu\mathrm{g}_{\mathrm{C}}^{-1}$) and pinonic acid ($C_{10}H_{16}O$; $\mathcal{C}^{\mathrm{MW}} = 184\ \mu\mathrm{g}\ \mathrm{mol}^{-1}/(10 \cdot 12\ \mu\mathrm{g}_{\mathrm{C}}\ \mathrm{mol}^{-1}) = 1.53\ \mu\mathrm{g}\ \mu\mathrm{g}_{\mathrm{C}}^{-1}$).

This estimate relies primarily on data from Park et al.[40] for an orange orchard in California; an alternative one based on Vermeuel et al.[42] for a ponderosa pine forest yielded fully comparable numbers ($\Gamma_{\mathrm{SVOCs}} = 0.57 - 0.73$ µg cm^{-2}; $|F_{\mathrm{OBVOCs}}| = 2.6 \cdot 10^{11}\ \mathrm{atom}_{\mathrm{C}}\ \mathrm{cm}^{-2}\mathrm{s}^{-1}$, $\mathrm{LAI} = 1.2\ \mathrm{m}^2_{\mathrm{leaf}}\ \mathrm{m}^{-2}_{\mathrm{soil}}$).

<u>Bottom-up estimate</u>

$$\Gamma_{\mathrm{PAHs}} = N_{\mathrm{PAHs}} \cdot \gamma_{\mathrm{PAH}} \cdot \mathrm{LMA} \cdot 10^{-7} \qquad \text{(S9)}$$

The surface concentration of PAHs on leaves (Γ_{PAHs}, in µg cm^{-2}) is computed from the normalized surface concentration per gram of plant ($\gamma_{\mathrm{PAH}} = 0.8 - 121\ \mathrm{ng}_{\mathrm{PAH}}\ \mathrm{specie}^{-1}\ \mathrm{g}^{-1}_{\mathrm{leaf,DW}}$; Figure S2, left panels), the number of PAHs per measurement ($N_{\mathrm{PAHs}} = 5 - 16$; Figure S2, right panel), and the leaf mass per unit of surface area ($\mathrm{LMA} = 30 - 330\ \mathrm{g}_{\mathrm{leaf,DW}}\ \mathrm{m}^{-2}_{\mathrm{leaf}}$)[7]. The multiplier accounts for units' conversion ($10^{-7} = 10^{-3}\ \mu\mathrm{g}\ \mathrm{ng}^{-1} \cdot 10^{-4}\ \mathrm{m}^2\ \mathrm{cm}^{-2}$).

This equation assumes that 100% of the mass is present on *the surface* of the cuticle. The choice of using *normalized* surface concentrations stems from the fact that different authors detected a different number of individual PAHs (5 – 21; Figure S2, right) but generally reported only the sum of their concentration. We did not distinguish between urban and pristine environments because we found differences to be minimal (e.g., Gong et al.[39] vs. Huang et al.[43]).

<u>Additional considerations</u> As we highlight in the main text, the surface mass coverage for individual compounds is always considerably lower than for compound classes. For the specific

case of SVOCs, we estimated $\hat{\Gamma}_{\mathrm{PAHs}}/\hat{\Gamma}_{\mathrm{SVOCs}} \approx 0.12\%$, comparable to what reported by Lam et al.[45] for PAHs in urban grime (0.02%).

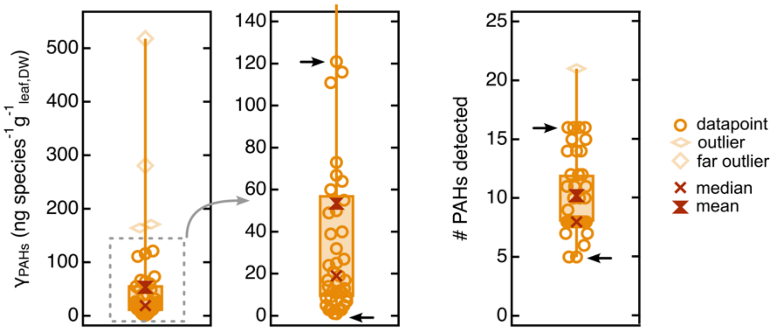

Figure S2 Overview of data used for estimating Γ_{SVOCs}. Numeric values are replotted from Gong et al.[39] (data from Table S3), Huang et al.[43] (data from Tables 3 – 4), and Franzaring and Van der Eerden[44] (data from Figure 1, extracted with WebPlot Digitizer[30]). As various authors reported overall PAHs concentration summing values for a different number of PAHs, we normalize the surface cover per number of detected species (# PAHs detected, or N_{PAHs}; right). Ranges for γ_{PAH} and N_{PAHs} are taken as minimum and maximum excluding outliers (black arrows; N = 43). Boxplots were obtained with Igor Pro 8 (version 8.04).

Exogenous compounds, wet deposition

Rain

$$\Gamma_{\mathrm{rain}} = \frac{2}{3} \cdot \frac{d_{\mathrm{drop}}}{\gamma^2} \cdot \frac{\mathrm{TOC}_{\mathrm{rain}}}{f_{\mathrm{TOC/TM}}} \cdot f_{\mathrm{intercepted}} \cdot f_{\mathrm{retained}} \cdot 10^{-1} \qquad \text{(S10)}$$

The surface concentration of chemicals delivered by rain (Γ_{rain}, in µg cm^{-2}) is calculated from the diameter of rain drops ($d_{\mathrm{drop}} = 0.1 - 5.5$ mm),[46–48] a factor that accounts for the increase in raindrop diameter after their impact on the leaf ($\gamma \approx 3$), the total organic carbon concentration of rain samples ($\mathrm{TOC}_{\mathrm{rain}} = 0.02 - 12.97\ \mathrm{mg_C\ L^{-1}}$; Figure S3),[49] the fraction of carbon mass to the total mass ($f_{\mathrm{TOC/TM}} \approx 0.5$), the faction of rain intercepted by vegetation ($f_{\mathrm{intercepted}} = 0.01 - 0.5$),[38,50] the fraction of drops retained on the leaf ($f_{\mathrm{retained}} \approx 0.5$), and a unit conversion factor (10^{-1} = cm mm^{-1}).

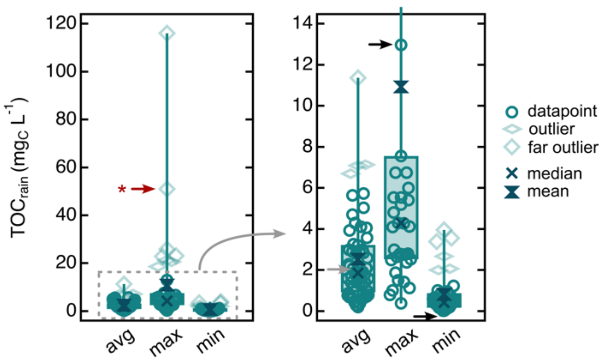

Figure S3 Overview of data used for estimating Γ_{rain}. Numeric values are replotted from Iavorivska et al.[49] (supplementary table) separated into average (avg), maximum (max), and minimum (min) values. As ranges for TOC_{rain}, we took the minimum of minima and the maximum of maxima (excluding outliers) for all reported data (black arrows, $N = 35 - 37$; the analysis excludes hurricanes and rain/snow samples). For our estimate, we also consider the case of a far outlier (marked red arrow in the left panel) and the average of averages (grey arrow in the right panel). Boxplots were obtained with Igor Pro 8 (version 8.04).

This calculation assumes that leaves are completely covered by rain drops of uniform diameter d_{drop} and carbon content TOC_{rain}. When impacting the leaf, drops deform, occupying a circular area of diameter $d'_{\text{drop}} = d_{\text{drop}} \cdot \gamma$, with $\gamma > 1$. To make the estimate more realistic, we additionally assume that only a fraction of leaves gets into contact with drops ($f_{\text{intercepted}}$) and that only a fraction of drops remains on each leaf until evaporation (f_{retained}). The first factor acknowledges that, within the canopy, not all leaves are horizontal and/or get exposed to rain, whereas the second accounts for the fact that drops may roll off before evaporation (which occurs when the amount of rainfall reaches the leaf water holding capacity). Last, we assume $f_{\text{TOC/TM}} \approx 0.5$ based on typical carbon content of aquatic and soil organic matter (46 – 64% of the total mass).[51] The derivation of equation (S10) is provided below.

<u>Derivation</u>. We consider a horizontal leaf of area A_{leaf} and spherical raindrops of diameter d_{drop}. After impacting the leaf, each drop increases its diameter to $d'_{\text{drop}} = \gamma \cdot d_{\text{drop}}$ ($\gamma > 1$). The maximum number of drops on the leaf ($N_{\text{drop}}^{\max}$) can be estimated from the ratio between the area occupied by each splashed drop and A_{leaf}, i.e., $N_{\text{drop}}^{\max} \approx A_{\text{leaf}} / A'_{\text{drop}}$. If 100% of the drops evaporate, the total mass of carbon delivered on the surface ($m_{\text{C,rain}}$) is

$$m_{\text{C,rain}} = N_{\text{drop}}^{\max} \cdot V_{\text{drop}} \cdot \text{TOC}_{\text{rain}}\,, \qquad \text{(S10.1)}$$

where V_{drop} is the volume of each drop. By substituting the definition of $N_{\text{drop}}^{\max}$ and $f_{\text{TOC/TM}} = m_{\text{C}}/m$, one obtains equation (S10.2).

$$m_{\mathrm{rain}} = A_{\mathrm{leaf}} \cdot \frac{V_{\mathrm{drop}}}{A_{\mathrm{drop}}} \cdot \frac{\mathrm{TOC_{rain}}}{f_{\mathrm{TOC/TM}}}, \tag{S10.2}$$

with

$$\frac{V_{\mathrm{drop}}}{A'_{\mathrm{drop}}} = \frac{\frac{\pi}{6} \cdot d_{\mathrm{drop}}^3}{\frac{\pi}{4} \cdot \left(\gamma \cdot d_{\mathrm{drop}}\right)^2} = \frac{2}{3} \cdot \frac{d_{\mathrm{drop}}}{\gamma^2}. \tag{S10.3}$$

To make the approximation more realistic, we multiply equation (S10.2) by $f_{\mathrm{intercepted}}$ and f_{retained}. If we take the resulting formula, substitute the definition $\Gamma_{\mathrm{rain}} = m_{\mathrm{rain}}/A_{\mathrm{leaf}}$, and account for units' conversion ($\mathrm{mg_C\ L^{-1} = \mu g_C\ cm^{-3}}$; $10^{-1}\ \mathrm{cm\ mm^{-1}}$), we arrive to equation (S10).

<u>Additional considerations.</u> The maxima in Figure S3 show far outliers as high as 51 and 116 $\mathrm{mg_C\ L^{-1}}$. In particular, the first value was measured in rain samples collected in an area heavily impacted by sugar cane burning,[52] hinting that such high carbon content can be observed, e.g., in the presence of wildfires. (We could not identify the reason for the extreme value of the uppermost outlier.) Using $\mathrm{TOC_{rain}} = 51\ \mathrm{mg_C\ L^{-1}}$, we obtained $\Gamma_{\mathrm{rain}} = 0.00038 - 1.0\ \mathrm{\mu g\ cm^{-2}}$. In addition to this extreme case, we computed Γ_{rain} using $\mathrm{TOC_{rain}^{avg}} \approx 2\ \mathrm{mg_C\ L^{-1}}$, yielding $\Gamma_{\mathrm{rain}} = 0.000015 - 0.041\ \mathrm{\mu g\ cm^{-2}}$.

Fog

$$\Gamma_{\mathrm{fog}} = \frac{\mathrm{TOC_{fog}}}{f_{\mathrm{TOC/TM}}} \cdot h_{\mathrm{wetness}} \cdot 10^{-1} \tag{S11}$$

The surface concentration of chemicals delivered by fog (Γ_{fog}, in $\mathrm{\mu g\ cm^{-2}}$) is calculated from the total organic carbon concentration of fog samples ($\mathrm{TOC_{fog}}$, in $\mathrm{mg_C\ L^{-1}}$), the fraction of carbon mass to the total mass ($f_{\mathrm{TOC/TM}} \approx 0.5$), and the thickness of the water film on leaves ($h_{\mathrm{wetness}} = 0.001 - 0.5\ \mathrm{mm}$)[53]. The multiplier accounts for units' conversion ($10^{-1} = \mathrm{cm\ mm^{-1}}$). For $\mathrm{TOC_{fog}}$, we consider two different ranges depending on the environment, namely $\mathrm{TOC_{fog}^{rural}} = 0.10 - 3.5\ \mathrm{mg_C\ L^{-1}}$ and $\mathrm{TOC_{fog}^{urban}} = 2.0 - 41\ \mathrm{mg_C\ L^{-1}}$ for rural/pristine and urban/polluted areas, respectively (Figure S4).

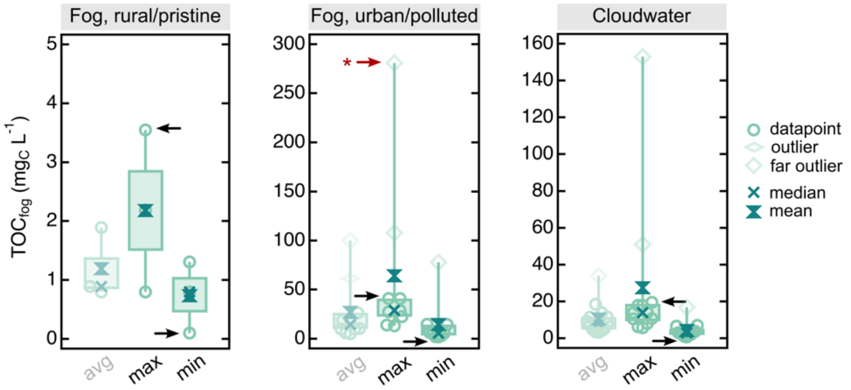

Figure S4 Data used for estimating Γ_{fog}. Numeric values are replotted from Herckes et al.[54] (Figure 2, data extracted with WebPlot Digitizer[30]) divided into average (avg), maximum (max), and minimum (min). As rural/pristine environments, we take the values reported for marine clouds and fog (N = 3), for urban/polluted environments we consider data for radiation fog and polluted urban fog (N = 9 – 10), while for cloud water we take data for hill and mountain intercepted clouds (including heavily marine influenced; N = 10 – 13). The ranges for TOC_{fog} are taken as the minimum of minima and the maximum of maxima excluding outliers (black arrows); for urban fog, we also consider the case of the top far outlier (marked red arrow). Boxplots were obtained with Igor Pro 8 (version 8.04).

This calculation assumes that leaves are covered by a homogeneous water layer of thickness h_{wetness}, and that all liquid remains on the leaf until evaporation. Similar to rain, we suppose $f_{\mathrm{TOC/TM}} \approx 0.5$ based on typical carbon content of aquatic and soil organic matter.[51] The derivation of equation (S11) is given below.

<u>Derivation</u> The mass of carbon dissolved in a uniform film of thickness h_{wetness} and concentration $\mathrm{TOC_{fog}}$ is

$$m_{\mathrm{C,fog}} = \mathrm{TOC_{fog}} \cdot h_{\mathrm{wetness}} \cdot A_{\mathrm{leaf}} \cdot 10^{-1}, \qquad \text{(S11.1)}$$

where $h_{\mathrm{wetness}} \cdot A_{\mathrm{leaf}}$ is the total volume of liquid on the leaf. By definition, $f_{\mathrm{TOC/TM}} = m_{\mathrm{C}}/m$, where m_{C} is the mass of carbon and m is the total mass. Thus,

$$m_{\mathrm{fog}} = \frac{m_{\mathrm{C,fog}}}{f_{\mathrm{TOC/TM}}} = \frac{\mathrm{TOC_{fog}} \cdot h_{\mathrm{wetness}} \cdot A_{\mathrm{leaf}} \cdot 10^{-1}}{f_{\mathrm{TOC/TM}}}. \qquad \text{(S11.2)}$$

By rearranging equation (S11.2) as $m_{\mathrm{fog}}/A_{\mathrm{leaf}} = \Gamma_{\mathrm{fog}}$, one gets equation (S11).

<u>Additional considerations</u> Using the values in Herckes et al.[54] for cloud water, we also computed the surface mass coverage in the presence of this other form of wetness, yielding $\Gamma_{\mathrm{clouds}} = 0.00014 - 1.95\ \mu\mathrm{g\ cm^{-2}}$ ($\mathrm{TOC_{clouds}} = 0.70 - 19\ \mathrm{mg_C\ L^{-1}}$; Figure S4) We further estimated the

maximum possible Γ_{fog} value in the presence of severe pollution using the top far outlier in Figure S4 ($281\ mg_C\ L^{-1}$), yielding $\Gamma_{fog} = 0.056 - 28\ \mu g\ cm^{-2}$.

Pesticides in agricultural sprays

$$\Gamma_{pesticide} = \gamma_{pesticide} \cdot \frac{SPB}{LAI} \cdot 10^{-1} \tag{S12}$$

The surface concentration of pesticides ($\Gamma_{pesticide}$, in µg cm^{-2}) is calculated from typical pesticide residues on leafy vegetables ($\gamma_{pesticide} = 0.01 - 36\ mg_{pesticide}\ kg^{-1}_{plant,DW}$),[55,56] typical values for the standing plant biomass ($SPB = 0.05 - 0.35\ kg_{plant,DW}\ m^{-2}_{soil}$),[37,38] the average leaf area index for crops ($LAI = 3.6\ m^2_{leaf}\ m^{-2}_{soil}$),[31] and a unit conversion factor ($10^{-1} = 10^3\ \mu g\ mg^{-1} \cdot 10^{-4}\ m^2\ cm^{-2}$).

This calculation is based on two contrasting assumptions. The first is that 100% of the residues are on *the surface* of the cuticle – thus, we neglect pesticides are embedded within the cuticle or taken up by the plant. The second assumption is that $\gamma_{pesticide}$ is representative of the amount of material that we can find on crops' leaves at any given time. However, right after application, concentrations are higher. For example, Das et al.[57] reported up to 20-fold higher leaf surface concentrations after application as compared to the value after 21 days. As sprays are actively applied on plants only in agricultural areas, we consider this contribution only for crops.

Table S1: Estimated contribution of individual compound classes to Γ_{tot} for the case-studies in Section 6.1.2 and Figure 9.

	Γ_i (µg cm^{-2})		
	**Case 1 (rain)**	**Case 1 (sun)**	**Case 2**
**Endogenous compounds**			
Trichomes	†	†	0.027 – 30 (1.3 – 14%)
Guttation	§	§	0.0000049 – 10 (0.0002 – 4.8%)
Resins	†	†	†
Phyllosphere	2.0 – 100 (86 – 63%)	2.0 – 100 (81 – 46%)	2.0 – 100 (99 – 48%)
**Exogenous compounds, dry deposition**			
PM	0.06 – 56* (2.6 – 36%)	0.20 – 115 (8.1 – 53%)	§
Pollen	§	§	§
Soil particles	§	§	0.0025 – 63* (0.13 – 30%)
SVOCs	0.28 – 1.7 (12 – 1.1%)	0.28 – 1.7 (11 – 0.8%)	§
**Exogenous compounds, wet deposition**			
Rain	0.00000015 – 0.26 (≈ 0 – 0.17%)	§	0.00000015 – 0.26 (≈ 0 – 0.13%)
Fog	§	§	§
Pesticides	§	§	0.00014 – 7.0* (0.007 – 3.3%)
**Total**	**2.3 – 158**	**2.5 – 217**	**2.0 – 210**

Percentages in grey are ranges of relative contributions given as ($\Gamma_i^{min}/\Gamma_{tot}^{min} \cdot 100$ to $\Gamma_i^{max}/\Gamma_{tot}^{max} \cdot 100$). For case 1, we reported both situations described in the man text. Legend: †, not applicable (based on biological constraints); §, not expected (unsuitable environmental conditions, unlikely location, or other situation-specific reasons); *, scaled as described in the main text.

Text S3: Details on global extrapolation of organic mass on leaf surfaces

$$m_{\mathrm{tot,global}}^{\mathrm{min}} = \Gamma_{\mathrm{tot}}^{\mathrm{min}} \cdot A_{\mathrm{land,global}} \cdot 10^4 \quad \text{(S13)}$$

For a lower limit estimate of the mass of organic present globally on leaves ($m_{\mathrm{tot,global}}^{\mathrm{min}}$, in Tg = 10^{12} g), we multiplied the minimum total surface mass coverage obtained in our case-studies ($\Gamma_{\mathrm{tot}}^{\mathrm{min}} \approx 2$ µg cm^{-2}; see Table S1) by the total land surface on Earth ($A_{\mathrm{land,global}} \approx 1.5 \cdot 10^8$ km^2) and a unit conversion's factor ($10^4 = 10^4\ \mathrm{cm}^2\ \mathrm{m}^{-2} \cdot 10^{-6}\ \mathrm{g}\ \mu\mathrm{g}^{-1} \cdot 10^6\ \mathrm{m}^2\ \mathrm{km}^{-2}$).

We stress that this calculation is highly conservative due to the approximations it relies on. First, as surface mass coverage we use the *minimum* of an interval that spans two orders of magnitude ($\Gamma_{\mathrm{tot}} \approx 2 - 200$ µg cm^{-2}; Table S1). Second, Γ_{tot} includes only known sources of chemicals; as we clarify in the main text, we expect sizeable contributions also from endogenous compounds leached from the leaf interior in the presence of surface wetness – thus, $\Gamma_{\mathrm{tot}}^{\mathrm{min}} > 2$ µg cm^{-2}. Third, we conservatively assume that the total leaf surface area equals $A_{\mathrm{land,global}}$. This choice is based on statements from the phyllosphere literature, where the total leaf surface area has been estimated as $2 \cdot 10^8 - 1 \cdot 10^9$ km^2.[16,21,58–60] Although these papers generally lack to clarify how these numbers are obtained, they agree with alternative estimates. Specifically, the global leaf surface area ($A_{\mathrm{leaf,global}}$) can be calculated as $A_{\mathrm{leaf,global}} = A_{\mathrm{land,global}} \cdot f_{\mathrm{vegetation}} \cdot \mathrm{LAI}$, where $f_{\mathrm{vegetation}}$ is the fraction of land covered by vegetation (≈ 0.74),[61] and $\mathrm{LAI} = 1.31 - 6.34\ \mathrm{m}_{\mathrm{leaf}}^2\ \mathrm{m}_{\mathrm{soil}}^{-2}$ (values are minimum and maximum of averages for various biomes).[31] This calculation yields $A_{\mathrm{leaf,global}} = 1.45 - 7.04 \cdot 10^8$ km^2; when combined with the other two approximations, we obtain $m_{\mathrm{tot,global}} > 2.9 - 1410$ Tg of leaf surface organics.

References

(1) Matzke, K.; Riederer, M. A Comparative Study into the Chemical Constitution of Cutins and Suberins from Picea Abies (L.) Karst., Quercus Robur L., and Fagus Sylvatica L. *Planta* **1991**, *185* (2), 233–245. https://doi.org/10.1007/BF00194066.

(2) Gülz, P.-G.; Müller, E.; Prasad, R. B. N. Organ-Specific Composition of Epicuticular Waxes of Beech (Fagus Sylvatica L.) Leaves and Seeds. *Z. Für Naturforschung C* **1989**, *44* (9–10), 731–734. https://doi.org/10.1515/znc-1989-9-1004.

(3) Gülz, P.-G.; Prasad, R. B. N.; Müller, E. Surface Structures and Chemical Composition of Epicuticular Waxes during Leaf Development of Fagus Sylvatica L. *Z. Für Naturforschung C* **1992**, *47* (3–4), 190–196. https://doi.org/10.1515/znc-1992-3-404.

(4) Naß, R.; Markstädter, C.; Hauke, V.; Riederer, M. Quantitative Gas Chromatographic Analysis of Plant Cuticular Waxes Containing Long-Chain Aldehydes. *Phytochem. Anal.* **1998**, *9* (3), 112–118.

(5) Reynhardt, E. C.; Riederer, M. Structures and Molecular Dynamics of Plant Waxes. *Eur. Biophys. J.* **1994**, *23* (1), 59–70. https://doi.org/10.1007/BF00192206.

(6) Wagner, G. J.; Wang, E.; Shepherd, R. W. New Approaches for Studying and Exploiting an Old Protuberance, the Plant Trichome. *Ann. Bot.* **2004**, *93* (1), 3–11. https://doi.org/10.1093/aob/mch011.

(7) Poorter, H.; Niinemets, Ü.; Poorter, L.; Wright, I. J.; Villar, R. Causes and Consequences of Variation in Leaf Mass per Area (LMA): A Meta-Analysis. *New Phytol.* **2009**, *182* (3), 565–588. https://doi.org/10.1111/j.1469-8137.2009.02830.x.

(8) Urbaneja-Bernat, P.; Tena, A.; González-Cabrera, J.; Rodriguez-Saona, C. Plant Guttation Provides Nutrient-Rich Food for Insects. *Proc. R. Soc. B Biol. Sci.* **2020**, *287* (1935), 20201080. https://doi.org/10.1098/rspb.2020.1080.

(9) He, C.; Qiu, K.; Pott, R. Reduction of Urban Traffic–Related Particulate Matter—Leaf Trait Matters. *Environ. Sci. Pollut. Res.* **2020**, *27* (6), 5825–5844. https://doi.org/10.1007/s11356-019-07160-0.

(10) Liu, Z.; Zhu, Y.; Li, F.; Jin, G. Non-Destructively Predicting Leaf Area, Leaf Mass and Specific Leaf Area Based on a Linear Mixed-Effect Model for Broadleaf Species. *Ecol. Indic.* **2017**, *78*, 340–350. https://doi.org/10.1016/j.ecolind.2017.03.025.

(11) PubChem. *Abietic acid*. https://pubchem.ncbi.nlm.nih.gov/compound/10569 (accessed 2023-08-31).

(12) Eller, A. S. D.; Harley, P.; Monson, R. K. Potential Contribution of Exposed Resin to Ecosystem Emissions of Monoterpenes. *Atmos. Environ.* **2013**, *77*, 440–444. https://doi.org/10.1016/j.atmosenv.2013.05.028.

(13) Mellor, G. E.; Tregunna, E. B. The Relationship Between Leaf Area and Leaf Dry Weight of Three Conifer Species Grown on Three Sources of Nitrogen. *Can. J. For. Res.* **1972**, *2* (3), 377–379. https://doi.org/10.1139/x72-058.

(14) Holopainen, J. K.; Himanen, S. J.; Yuan, J. S.; Chen, F.; Stewart, C. N. Ecological Functions of Terpenoids in Changing Climates. In *Natural Products: Phytochemistry, Botany and Metabolism of Alkaloids, Phenolics and Terpenes*; Ramawat, K. G., Mérillon, J.-M., Eds.; Springer: Berlin, Heidelberg, 2013; pp 2913–2940. https://doi.org/10.1007/978-3-642-22144-6_129.

(15) Langenheim, J. H. Plant Resins. *Am. Sci.* **1990**, *78* (1), 16–24.

(16) Lindow, S. E.; Brandl, M. T. Microbiology of the Phyllosphere. *Appl. Environ. Microbiol.* **2003**, *69* (4), 1875–1883. https://doi.org/10.1128/AEM.69.4.1875-1883.2003.

(17) Cayley, S.; Lewis, B. A.; Guttman, H. J.; Record, M. T. Characterization of the Cytoplasm of Escherichia Coli K-12 as a Function of External Osmolarity: Implications for Protein-DNA Interactions in Vivo. *J. Mol. Biol.* **1991**, *222* (2), 281–300. https://doi.org/10.1016/0022-2836(91)90212-O.

(18) Philips, R. M. & R. *» How big is an E. coli cell and what is its mass?* http://book.bionumbers.org/how-big-is-an-e-coli-cell-and-what-is-its-mass/ (accessed 2023-08-31).

(19) Donlan, R. M. Biofilms: Microbial Life on Surfaces. *Emerg. Infect. Dis.* **2002**, *8* (9), 881–890. https://doi.org/10.3201/eid0809.020063.

(20) Vacher, C.; Hampe, A.; Porté, A. J.; Sauer, U.; Compant, S.; Morris, C. E. The Phyllosphere: Microbial Jungle at the Plant–Climate Interface. *Annu. Rev. Ecol. Evol. Syst.* **2016**, *47* (1), 1–24. https://doi.org/10.1146/annurev-ecolsys-121415-032238.

(21) Vorholt, J. A. Microbial Life in the Phyllosphere. *Nat. Rev. Microbiol.* **2012**, *10* (12), 828–840. https://doi.org/10.1038/nrmicro2910.

(22) Łabędź, B.; Wańczyk, A.; Rajfur, Z. Precise Mass Determination of Single Cell with Cantilever-Based Microbiosensor System. *PLOS ONE* **2017**, *12* (11), e0188388. https://doi.org/10.1371/journal.pone.0188388.

(23) Haddad, S. A.; Lindegren, C. C. A Method for Determining the Weight of an Individual

Yeast Cell. *Appl. Microbiol.* **1953**, *1* (3), 153–156.

(24) Glushakova, A. M.; Chernov, I. Yu. Seasonal Dynamic of the Numbers of Epiphytic Yeasts. *Microbiology* **2007**, *76* (5), 590–595. https://doi.org/10.1134/S0026261707050128.

(25) Corada, K.; Woodward, H.; Alaraj, H.; Collins, C. M.; de Nazelle, A. A Systematic Review of the Leaf Traits Considered to Contribute to Removal of Airborne Particulate Matter Pollution in Urban Areas. *Environ. Pollut.* **2021**, *269*, 116104. https://doi.org/10.1016/j.envpol.2020.116104.

(26) Jimenez, J. L.; Canagaratna, M. R.; Donahue, N. M.; Prevot, A. S. H.; Zhang, Q.; Kroll, J. H.; DeCarlo, P. F.; Allan, J. D.; Coe, H.; Ng, N. L.; Aiken, A. C.; Docherty, K. S.; Ulbrich, I. M.; Grieshop, A. P.; Robinson, A. L.; Duplissy, J.; Smith, J. D.; Wilson, K. R.; Lanz, V. A.; Hueglin, C.; Sun, Y. L.; Tian, J.; Laaksonen, A.; Raatikainen, T.; Rautiainen, J.; Vaattovaara, P.; Ehn, M.; Kulmala, M.; Tomlinson, J. M.; Collins, D. R.; Cubison, M. J.; E; Dunlea, J.; Huffman, J. A.; Onasch, T. B.; Alfarra, M. R.; Williams, P. I.; Bower, K.; Kondo, Y.; Schneider, J.; Drewnick, F.; Borrmann, S.; Weimer, S.; Demerjian, K.; Salcedo, D.; Cottrell, L.; Griffin, R.; Takami, A.; Miyoshi, T.; Hatakeyama, S.; Shimono, A.; Sun, J. Y.; Zhang, Y. M.; Dzepina, K.; Kimmel, J. R.; Sueper, D.; Jayne, J. T.; Herndon, S. C.; Trimborn, A. M.; Williams, L. R.; Wood, E. C.; Middlebrook, A. M.; Kolb, C. E.; Baltensperger, U.; Worsnop, D. R. Evolution of Organic Aerosols in the Atmosphere. *Science* **2009**, *326* (5959), 1525–1529. https://doi.org/10.1126/science.1180353.

(27) Cai, M.; Xin, Z.; Yu, X. Spatio-Temporal Variations in PM Leaf Deposition: A Meta-Analysis. *Environ. Pollut.* **2017**, *231*, 207–218. https://doi.org/10.1016/j.envpol.2017.07.105.

(28) Sullivan, A. P.; Weber, R. J.; Clements, A. L.; Turner, J. R.; Bae, M. S.; Schauer, J. J. A Method for On-Line Measurement of Water-Soluble Organic Carbon in Ambient Aerosol Particles: Results from an Urban Site. *Geophys. Res. Lett.* **2004**, *31* (13). https://doi.org/10.1029/2004GL019681.

(29) Feng, H.; Ding, Y.; Zou, B.; Cohen, J. B.; Ye, S.; Yang, Z.; Qin, K.; Liu, L.; Gu, X. Vegetation-Related Dry Deposition of Global PM2.5 from Satellite Observations. *J. Geogr. Sci.* **2022**, *32* (4), 589–604. https://doi.org/10.1007/s11442-022-1962-0.

(30) *WebPlotDigitizer - Copyright 2010-2019 Ankit Rohatgi*. https://apps.automeris.io/wpd/ (accessed 2020-05-11).

(31) Scurlock, J.; Asner, G.; Gower, S. Worldwide Historical Estimates of Leaf Area Index, 1932-2000. **2002**.

(32) Pleasants, J. M.; Hellmich, R. L.; Dively, G. P.; Sears, M. K.; Stanley-Horn, D. E.; Mattila, H. R.; Foster, J. E.; Clark, P.; Jones, G. D. Corn Pollen Deposition on Milkweeds in and near

Cornfields. *Proc. Natl. Acad. Sci.* **2001**, *98* (21), 11919–11924. https://doi.org/10.1073/pnas.211287498.

(33) Oldenburg, M.; Petersen, A.; Baur, X. Maize Pollen Is an Important Allergen in Occupationally Exposed Workers. *J. Occup. Med. Toxicol. Lond. Engl.* **2011**, *6*, 32. https://doi.org/10.1186/1745-6673-6-32.

(34) Hyde, P.; Mahalov, A. Contribution of Bioaerosols to Airborne Particulate Matter. *J. Air Waste Manag. Assoc.* **2020**, *70* (1), 71–77. https://doi.org/10.1080/10962247.2019.1629360.

(35) Smith, K. E. C.; Jones, K. C. Particles and Vegetation: Implications for the Transfer of Particle-Bound Organic Contaminants to Vegetation. *Sci. Total Environ.* **2000**, *246* (2), 207–236. https://doi.org/10.1016/S0048-9697(99)00459-3.

(36) Stockmann, U.; Padarian, J.; McBratney, A.; Minasny, B.; de Brogniez, D.; Montanarella, L.; Hong, S. Y.; Rawlins, B. G.; Field, D. J. Global Soil Organic Carbon Assessment. *Glob. Food Secur.* **2015**, *6*, 9–16. https://doi.org/10.1016/j.gfs.2015.07.001.

(37) Gonze, M.-A.; Sy, M. M. Interception of Wet Deposited Atmospheric Pollutants by Herbaceous Vegetation: Data Review and Modelling. *Sci. Total Environ.* **2016**, *565*, 49–67. https://doi.org/10.1016/j.scitotenv.2016.04.024.

(38) Hoffman, O. F.; Frank, M. L.; Blaylock, B. G.; von Bernuth, R. D.; Deming, E. J.; Graham, R. V.; Mohrbacher, D. A.; Waters, A. E. *Pasture Grass Interception and Retention of 131l, 7Be, and Insoluble Microspheres Deposited in Rain*; ORNL-6542; 3247; Oak Ridge National Laboratory: Environmental Sciences Division, 1989.

(39) Gong, P.; Xu, H.; Wang, C.; Chen, Y.; Guo, L.; Wang, X. Persistent Organic Pollutant Cycling in Forests. *Nat. Rev. Earth Environ.* **2021**, *2* (3), 182–197. https://doi.org/10.1038/s43017-020-00137-5.

(40) Park, J.-H.; Goldstein, A. H.; Timkovsky, J.; Fares, S.; Weber, R.; Karlik, J.; Holzinger, R. Active Atmosphere-Ecosystem Exchange of the Vast Majority of Detected Volatile Organic Compounds. *Science* **2013**, *341* (6146), 643–647. https://doi.org/10.1126/science.1235053.

(41) Fares, S.; Park, J.-H.; Gentner, D. R.; Weber, R.; Ormeño, E.; Karlik, J.; Goldstein, A. H. Seasonal Cycles of Biogenic Volatile Organic Compound Fluxes and Concentrations in a California Citrus Orchard. *Atmospheric Chem. Phys.* **2012**, *12* (20), 9865–9880. https://doi.org/10.5194/acp-12-9865-2012.

(42) Vermeuel, M. P.; Millet, D. B.; Farmer, D. K.; Pothier, M. A.; Link, M. F.; Riches, M.; Williams, S.; Garofalo, L. A. Closing the Reactive Carbon Flux Budget: Observations From Dual Mass Spectrometers Over a Coniferous Forest. *J. Geophys. Res. Atmospheres* **2023**, *128* (14),

e2023JD038753. https://doi.org/10.1029/2023JD038753.

(43) Huang, S.; Dai, C.; Zhou, Y.; Peng, H.; Yi, K.; Qin, P.; Luo, S.; Zhang, X. Comparisons of Three Plant Species in Accumulating Polycyclic Aromatic Hydrocarbons (PAHs) from the Atmosphere: A Review. *Environ. Sci. Pollut. Res.* **2018**, *25* (17), 16548–16566. https://doi.org/10.1007/s11356-018-2167-z.

(44) Franzaring, J.; van der Eerden, L. J. M. Accumulation of Airborne Persistent Organic Pollutants (POPs) in Plants. *Basic Appl. Ecol.* **2000**, *1* (1), 25–30. https://doi.org/10.1078/1439-1791-00003.

(45) Lam, B.; Diamond, M. L.; Simpson, A. J.; Makar, P. A.; Truong, J.; Hernandez-Martinez, N. A. Chemical Composition of Surface Films on Glass Windows and Implications for Atmospheric Chemistry. *Atmos. Environ.* **2005**, *39* (35), 6578–6586. https://doi.org/10.1016/j.atmosenv.2005.07.057.

(46) Serio, M. A.; Carollo, F. G.; Ferro, V. Raindrop Size Distribution and Terminal Velocity for Rainfall Erosivity Studies. A Review. *J. Hydrol.* **2019**, *576*, 210–228. https://doi.org/10.1016/j.jhydrol.2019.06.040.

(47) Levia, D. F.; Nanko, K.; Amasaki, H.; Giambelluca, T. W.; Hotta, N.; Iida, S.; Mudd, R. G.; Nullet, M. A.; Sakai, N.; Shinohara, Y.; Sun, X.; Suzuki, M.; Tanaka, N.; Tantasirin, C.; Yamada, K. Throughfall Partitioning by Trees. *Hydrol. Process.* **2019**, *33* (12), 1698–1708. https://doi.org/10.1002/hyp.13432.

(48) Hoffman, O. F.; Thiessen, K. M.; Rael, R. M. Comparison of Interception and Initial Retention of Wet-Deposited Contaminants on Leaves of Different Vegetation Types. *Atmos. Environ.* **1995**, *29* (15), 1771–1775. https://doi.org/10.1016/1352-2310(95)00099-K.

(49) Iavorivska, L.; Boyer, E. W.; DeWalle, D. R. Atmospheric Deposition of Organic Carbon via Precipitation. *Atmos. Environ.* **2016**, *146*, 153–163. https://doi.org/10.1016/j.atmosenv.2016.06.006.

(50) Kinnersley, R. P.; Goddard, A. J. H.; Minski, M. J.; Shaw, G. Interception of Caesium-Contaminated Rain by Vegetation. *Atmos. Environ.* **1997**, *31* (8), 1137–1145. https://doi.org/10.1016/S1352-2310(96)00312-3.

(51) *Elemental Compositions and Stable Isotopic Ratios of IHSS Samples | IHSS*. https://humic-substances.org/elemental-compositions-and-stable-isotopic-ratios-of-ihss-samples/ (accessed 2023-09-07).

(52) Coelho, C. H.; Francisco, J. G.; Nogueira, R. F. P.; Campos, M. L. A. M. Dissolved Organic Carbon in Rainwater from Areas Heavily Impacted by Sugar Cane Burning. *Atmos. Environ.* **2008**,

42 (30), 7115–7121. https://doi.org/10.1016/j.atmosenv.2008.05.072.

(53) Burkhardt, J.; Hunsche, M. “Breath Figures” on Leaf Surfaces—Formation and Effects of Microscopic Leaf Wetness. *Front. Plant Sci.* **2013**, *4*.

(54) Herckes, P.; Valsaraj, K. T.; Collett, J. L. A Review of Observations of Organic Matter in Fogs and Clouds: Origin, Processing and Fate. *Atmospheric Res.* **2013**, *132–133*, 434–449. https://doi.org/10.1016/j.atmosres.2013.06.005.

(55) Park, D. W.; Yang, Y. S.; Lee, Y.-U.; Han, S. J.; Kim, H. J.; Kim, S.-H.; Kim, J. P.; Cho, S. J.; Lee, D.; Song, N.; Han, Y.; Hee Kim, H.; Cho, B.-S.; Chung, J. K.; Kim, A. G. Pesticide Residues and Risk Assessment from Monitoring Programs in the Largest Production Area of Leafy Vegetables in South Korea: A 15-Year Study. *Foods* **2021**, *10* (2), 425. https://doi.org/10.3390/foods10020425.

(56) González-Rodríguez, R. M.; Rial-Otero, R.; Cancho-Grande, B.; Simal-Gándara, J. Occurrence of Fungicide and Insecticide Residues in Trade Samples of Leafy Vegetables. *Food Chem.* **2008**, *107* (3), 1342–1347. https://doi.org/10.1016/j.foodchem.2007.09.045.

(57) Das, S.; Hageman, K. J.; Taylor, M.; Michelsen-Heath, S.; Stewart, I. Fate of the Organophosphate Insecticide, Chlorpyrifos, in Leaves, Soil, and Air Following Application. *Chemosphere* **2020**, *243*, 125194. https://doi.org/10.1016/j.chemosphere.2019.125194.

(58) Morris, C. E. Phyllosphere. In *eLS*; John Wiley & Sons, Ltd, 2002. https://doi.org/10.1038/npg.els.0000400.

(59) Coutinho, T. A.; Bophela, K. N. Chapter 7 - Tree Leaves as a Habitat for Phyllobacteria. In *Forest Microbiology*; Asiegbu, F. O., Kovalchuk, A., Eds.; Forest Microbiology; Academic Press, 2021; pp 133–144. https://doi.org/10.1016/B978-0-12-822542-4.00001-2.

(60) Bringel, F.; Couée, I. Pivotal Roles of Phyllosphere Microorganisms at the Interface between Plant Functioning and Atmospheric Trace Gas Dynamics. *Front. Microbiol.* **2015**, *6*.

(61) *FAOSTAT*. https://www.fao.org/faostat/en/#data/LC/visualize (accessed 2022-09-29).
